# Synthesis and Evaluation of GM2-Monophosphoryl Lipid A Conjugate as a Fully Synthetic Self-Adjuvant Cancer Vaccine

**DOI:** 10.1038/s41598-017-11500-w

**Published:** 2017-09-12

**Authors:** Zhifang Zhou, Satadru S. Mandal, Guochao Liao, Jiatong Guo, Zhongwu Guo

**Affiliations:** 1 Department of Chemistry, Wayne State University, 5101 Cass Avenue, Detroit, Michigan 48202 United States; 2 International Institute for Translational Chinese Medicine, Guangzhou University of Chinese Medicine, 232 Waihuan Donglu, Guangdong, 510006 China; 3 Department of Chemistry, University of Florida, 214 Leigh Hall, Gainesville, Florida 32611 United States

## Abstract

An efficient method was developed for the synthesis of a GM2 derivative suitable for the conjugation with various biomolecules. This GM2 derivative was covalently linked to keyhole limpet hemocyanin (KLH) and monophosphoryl lipid A (MPLA) to form novel therapeutic cancer vaccines. Immunological evaluations of the resultant conjugates in mice revealed that they elicited robust GM2-specific overall and IgG antibody responses. Moreover, the GM2-MPLA conjugate was disclosed to elicit strong immune responses without the use of an adjuvant, proving its self-adjuvant property. The antisera of both conjugates showed strong binding and mediated similarly effective complement-dependent cytotoxicity to GM2-expressing cancer cell line MCF-7. Based on these results, it was concluded that both GM2-MPLA and GM2-KLH are promising candidates as therapeutic cancer vaccines, whereas fully synthetic GM2-MPLA, which has homogeneous and well-defined structure and self-adjuvant property, deserves more attention and studies.

## Introduction

Numerous unique or overexpressed glycans have been identified on various cancer cells, which are called tumor-associated carbohydrate antigens (TACAs)^1–3^. Typically, TACAs are exposed on the cell surface, rendering them excellent molecular targets for the development of therapeutic cancer vaccines or cancer immunotherapies^3–8^. However, like other carbohydrates, TACAs are usually poorly immunogenic and T cell independent^9^, thus they alone cannot elicit robust enough immune responses for effective cancer therapy^10^. The conventional strategy to address such problem is to couple TACAs covalently with a carrier protein, such as keyhole limpet hemocyanin (KLH)^11^, to form conjugate vaccines^12–14^, which is anticipated to significantly improve the immunogenicity of TACAs. This strategy has achieved great success in recent years. For example, several TACA-protein conjugates have entered different phases of clinical trials for cancer treatment^14, 15^. However, these conjugate vaccines possess some inherent drawbacks, such as having heterogeneous and ill-defined structures governed by carbohydrate-protein conjugation methods, which makes them to have batch-to-batch difference in physical, chemical, and immunological properties. Additionally, carrier proteins have been demonstrated to sometimes suppress competitively the immune response to carbohydrates^15, 16^. To address such issues, fully synthetic conjugate vaccines, which have well-defined structures and do not cause immunosuppression, are explored^17–34^. Along the line, we have identified monophosphoryl lipid A (MPLA) and its derivatives, which are potent immunological stimulants and adjuvants, as a new class of carrier molecules for the construction of fully synthetic self-adjuvant conjugate vaccines^35–38^. In the present work, this concept was used to develop cancer vaccines based on a branched sialotetrasaccharide TACA, the GM2 antigen.

Among all TACAs identified so far, GM2 is especially attractive for cancer vaccine development^39^ because: (a) GM2 is relatively cancer-specific and expressed by several types of tumors, including melanoma, sarcoma, and renal cancer;^40^ (b) GM2-reactive antibodies have been shown to mediate cytotoxicities against GM2-positive human cancer cell lines *in vitro*;^41–44^ (c) GM2 is comparatively immunogenic, as evidenced by the presence of low titers of natural anti-GM2 IgM antibodies in some cancer patients^44–46^ and by the findings that immunization of melanoma patients with GM2 conjugates could elicit anti-GM2 antibodies;^43, 44^ (d) the presence of GM2 antibodies in melanoma patients appears to be associated with improved survival and longer disease-free interval;^44^ (e) no deleterious side effects associated with immune responses to GM2 have been observed yet^44^. As a result, GM2 synthesis and GM2-based cancer vaccines have been explored extensively by several groups^47–51^. It was disclosed further that the GM2-KLH conjugate combined with QS-21 adjuvant could induce high titers of IgM and IgG antibodies in patients, thus this combination is in clinical trial to treat melanoma^44, 52, 53^.

**Figure 1:**
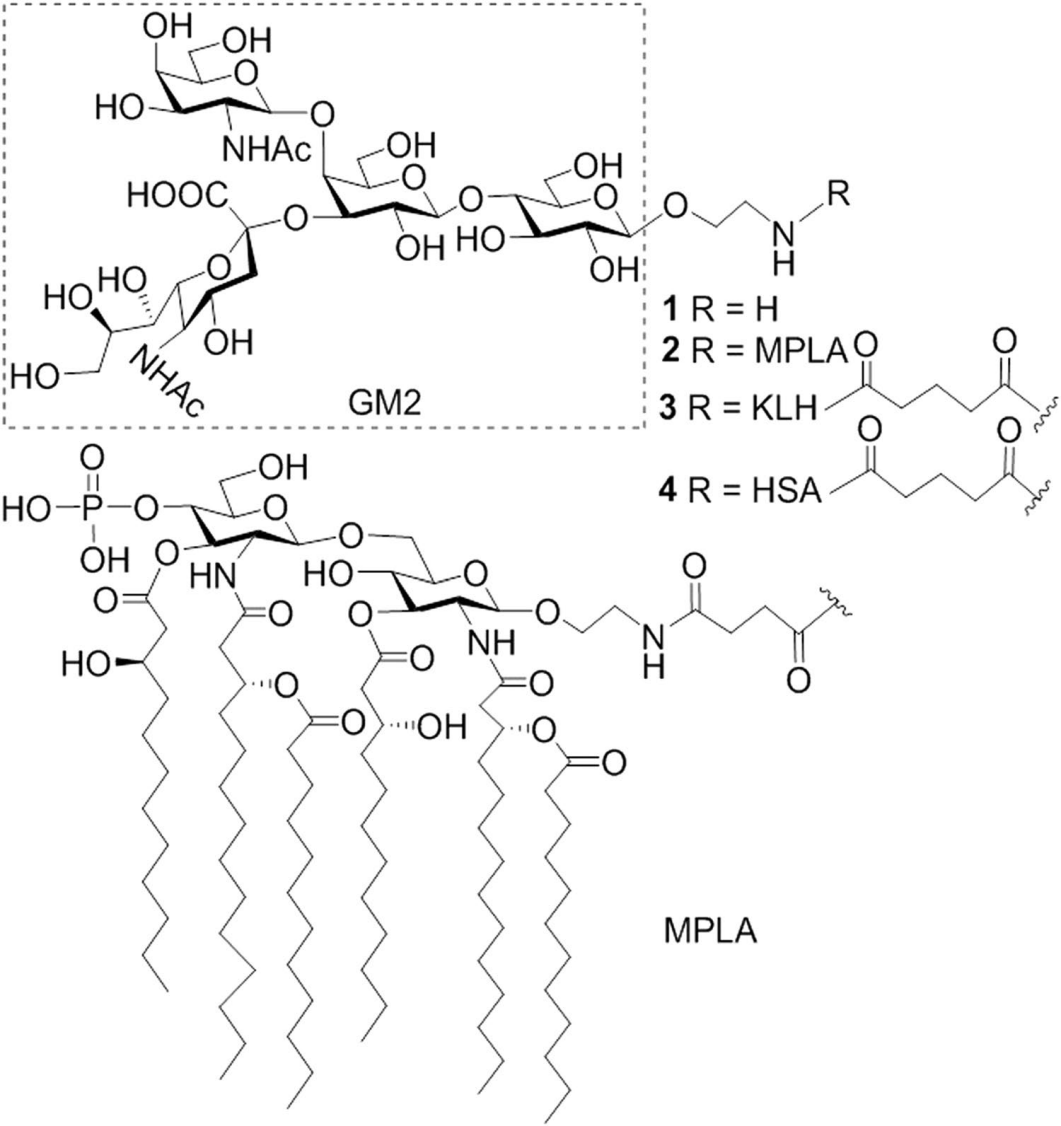
Structures of the synthesized GM2 derivative **1** and its MPLA, KLH, and HSA conjugates **2**-**4**.

Accordingly, we developed an efficient synthesis for a GM2 derivative **1** (Fig. 1) that had a free amino group linked to the glycan reducing end to facilitate its coupling with MPLA or other carrier molecules. *Neisseria meningitidis* MPLA, which was shown to be a functional carrier molecule for glycoconjugate vaccine development^36^, was selected in this study to create fully synthetic GM2-MPLA conjugate vaccine **2**. In the meantime, the KLH- and human serum albumin (HSA)-GM2 conjugates, **3** and **4**, were also synthesized and used as the positive control and capture reagent, respectively, in the immunological studies. Immunological responses of mice to the GM2-MPLA conjugate and GM2-KLH conjugate were evaluated and compared, so were the abilities of their antisera to bind to and kill cancer cells.

## Results and Discussion

### Synthesis of the MPLA and protein conjugates of GM2

**Figure 2:**
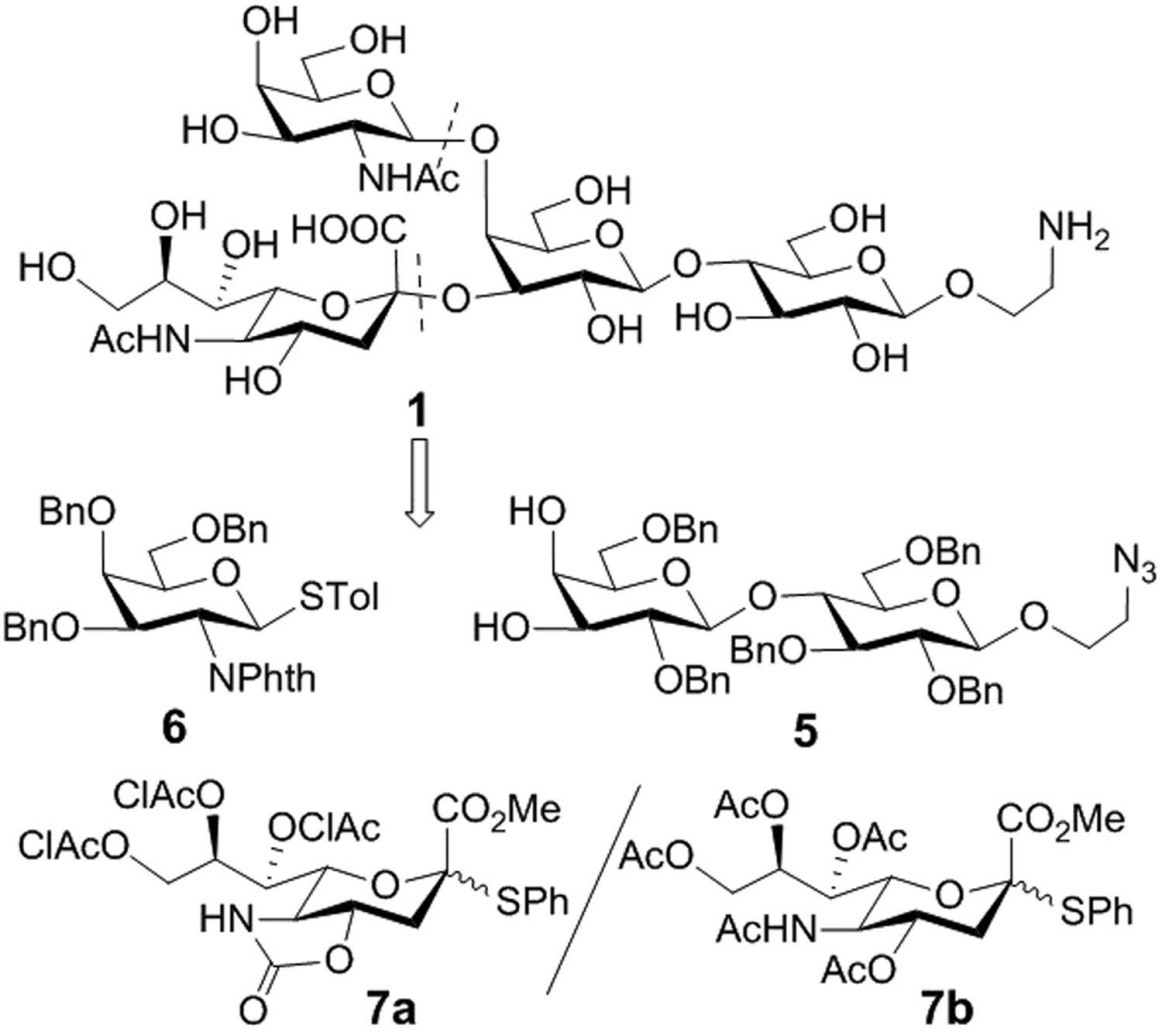
Retrosynthesis of the GM2 derivative **1**.

Retrosynthetic analysis of the target GM2 derivative **1** (Fig. 2) used for preparing GM2 conjugates **2**-**4** led to lactose derivative **5** as the glycosyl acceptor and **6** and **7** as glycosyl donors. Acceptor **5** was designed to be a diol that was relatively easily available while the different reactivities of its two free hydroxyl groups^54, 55^ would enable regioselective glycosylation for branched structure assembly in a highly convergent manner. Its reducing end had an azido group that would be converted into an amino group at the final stage for regioselective coupling of GM2 with carrier molecules. The 2-amino group of galactosaminyl donor **6** was protected with a phthalyl (Phth) group, which was expected to favor stereoselective 1,2-*trans* glycosylation as a result of neighboring group participation. Sialylation is one of the most difficult glycosylation reactions in carbohydrate synthesis^56^. Our initial plan to install the sialic acid residue was to use **7a** because this glycosyl donor gave excellent results in the literature^57^. Alternatively, we can also explore the sialylation reaction using **7b**. Although the stereochemical outcome of the latter reaction is sometimes difficult to predict, excellent results were also obtained with reactive glycosyl acceptors, such as primary alcohols;^54^ in addition, global deprotection of the final product derived from **7b** should be relative straightforward.

**Figure 3:**
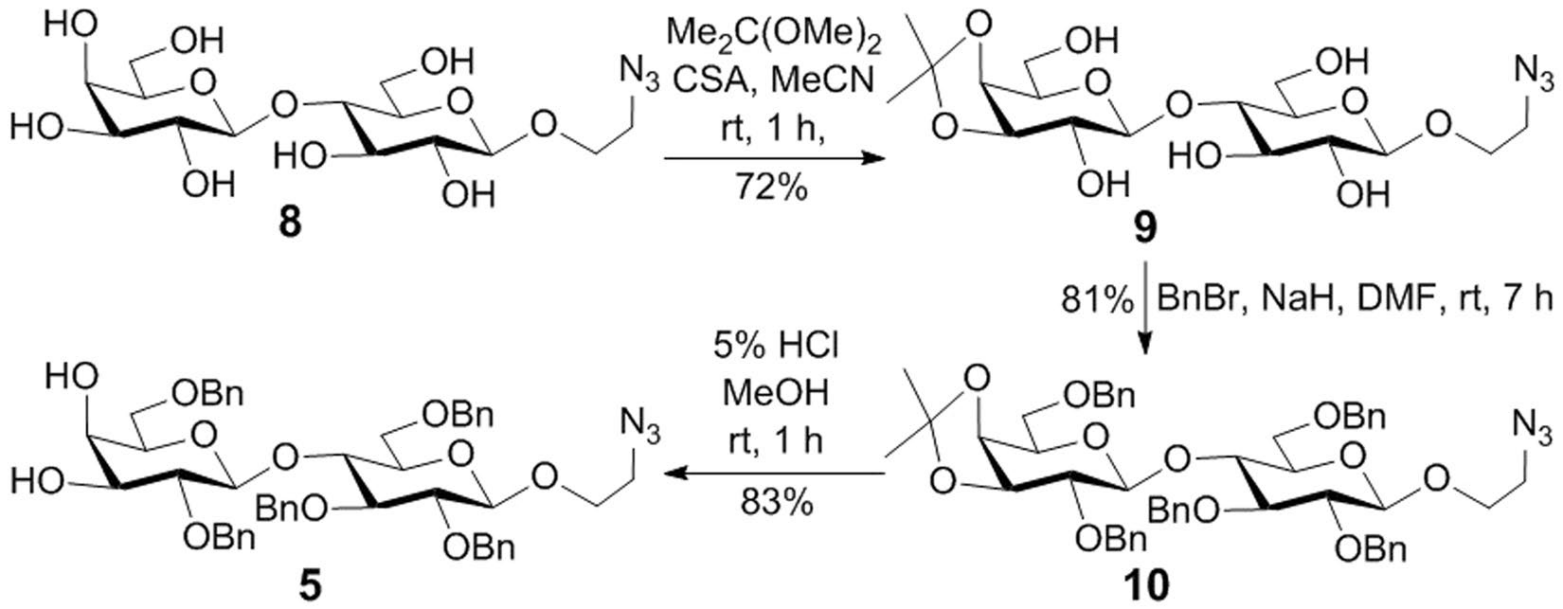
Synthesis of glycosyl acceptor **5**.

The synthesis of **5** (Fig. 3) commenced with preparing **8** from lactose. Selective protection of the 4′,6′-hydroxyl groups in **8** as acetonide was furnished by reacting with 2,2-dimethoxypropane (DMP) and camphorsulphonic acid (CSA), which was followed by benzylation using sodium hydride (NaH) and benzyl bromide (BnBr). Finally, the acetonide in the resultant **10** was removed with 5% HCl in methanol to afford **5**.

**Figure 4:**
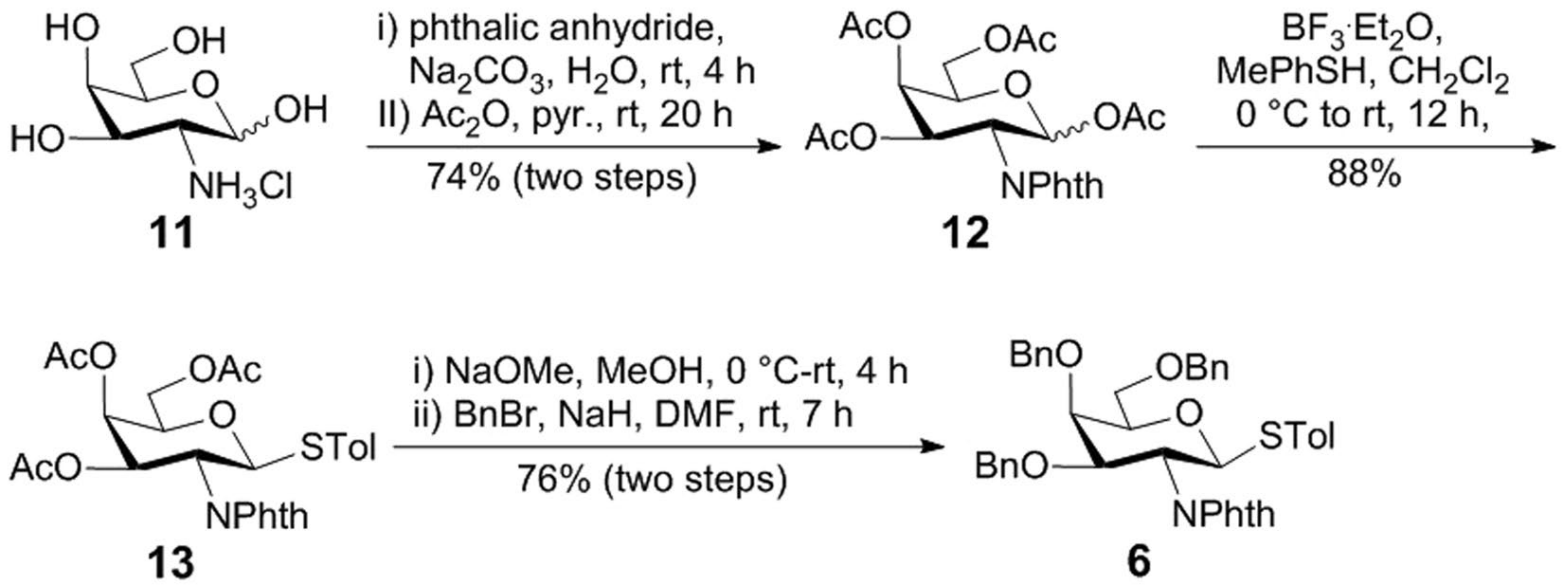
Synthesis of glycosyl donor **6**.

Sialyl donor **7a** was prepared according to a reported procedure^57^, and glucosaminyl donor **6** was readily obtained from D-galactosamine **11** after five established transformations (Fig. 4). Conversion of **11** into **12** was followed by thioglycosylation under the promotion of BF_3_·Et_2_O to afford **13** ^58^. Deacetylation of **13** with sodium methoxide in methanol and subsequent benzylation using NaH and BnBr eventually furnished **6** in a good overall yield.

**Figure 5:**
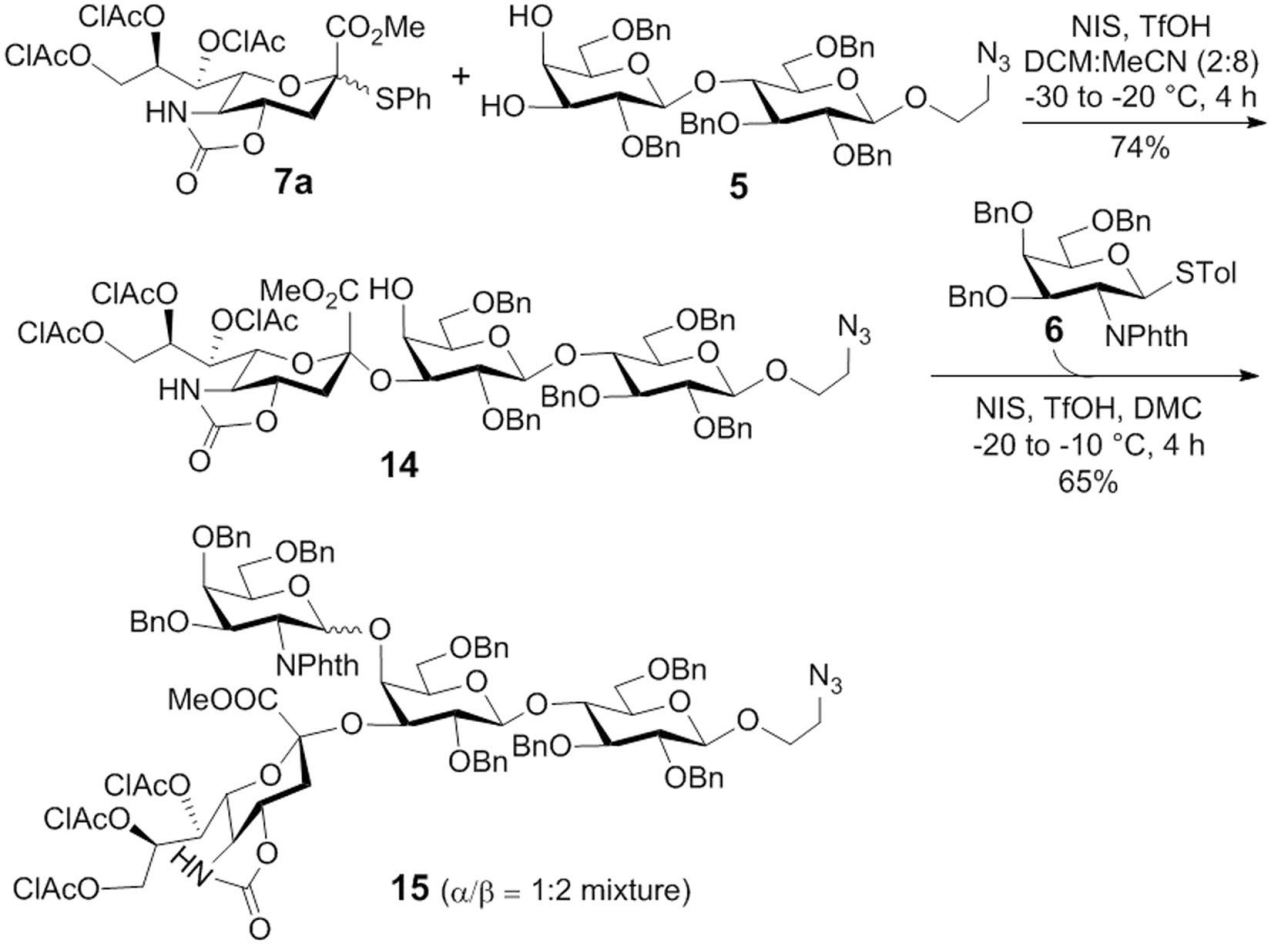
Synthesis of GM2 using glycosyl donors **7a** and **6**.

With all building blocks in hand, we initiated the assembly of GM2 with the 3-*O*-sialylation of **5** (Fig. 5). Because sialylation is typically more difficult than other glycosylation reactions, it was realized early in the synthesis to avoid any potential issue late on. Moreover, since the galactose 3-*O*-position is more reactive than its 4-*O*-position^54, 59, 60^, selective 3-*O*-glycosylation followed by 4-*O*-glycosylation of the 3,4-diol **5** can save at least two steps, i.e., 3-*O*-protection and deprotection. Most importantly, we noticed that a sugar unit at the galactose 4-*O*-position could block successive 3-*O*-glycosylation, thus to successfully assemble the branched structure in GM2, a 3-*O*- followed by 4-*O*-glycosylation sequence should be relatively reliable^61^. As expected, the reaction between **5** and **7a** in a 2:8 mixture of dichloromethane (DCM) and acetronitile under the promotion of *N*-iodosuccinimide (NIS) and triflic acid (TfOH) proceeded smoothly to afford trisaccharide **14** in a good yield (74%) and stereoselectivity ($\alpha :\beta$ 9:1). Its regiochemistry was confirmed by acetylation that resulted in a >1 ppm downfield shift of its H-4′ NMR signal. The α-configuration of the newly formed sialyl linkage was verified by the chemical shift of its H-3eq NMR signal at ${\rm{\delta }}$ 2.75 ppm (α-isomer: δ > 2.50; β-isomer: δ < 2.40)^59, 62^ and was also confirmed after the conversion of **14** to free GM3 that gave the same NMR spectra as that in the literature^63, 64^. Surprisingly, however, despite the presence of a participating Phth group at the 2-*N*-position of **6**, its reaction with **14** promoted by NIS/TfOH gave a poor stereoselectivity (*α:β* 1:2), although the conversion rate (65%) was good. Moreover, the two anomers were not easily separable. To improve the stereoselectivity of this step and the overall synthetic efficiency, we converted **6** to the corresponding trichloroacetimidate as a donor for glycosylation of **14** with trimethyl trifluoromethanesulfonate (TMSOTf) as the promoter, but the reaction gave **15** in a low yield (15%) without obvious improvement of stereoselectivity (α:β 1:2). In the meantime, orthoester was observed as the major side product.

**Figure 6:**
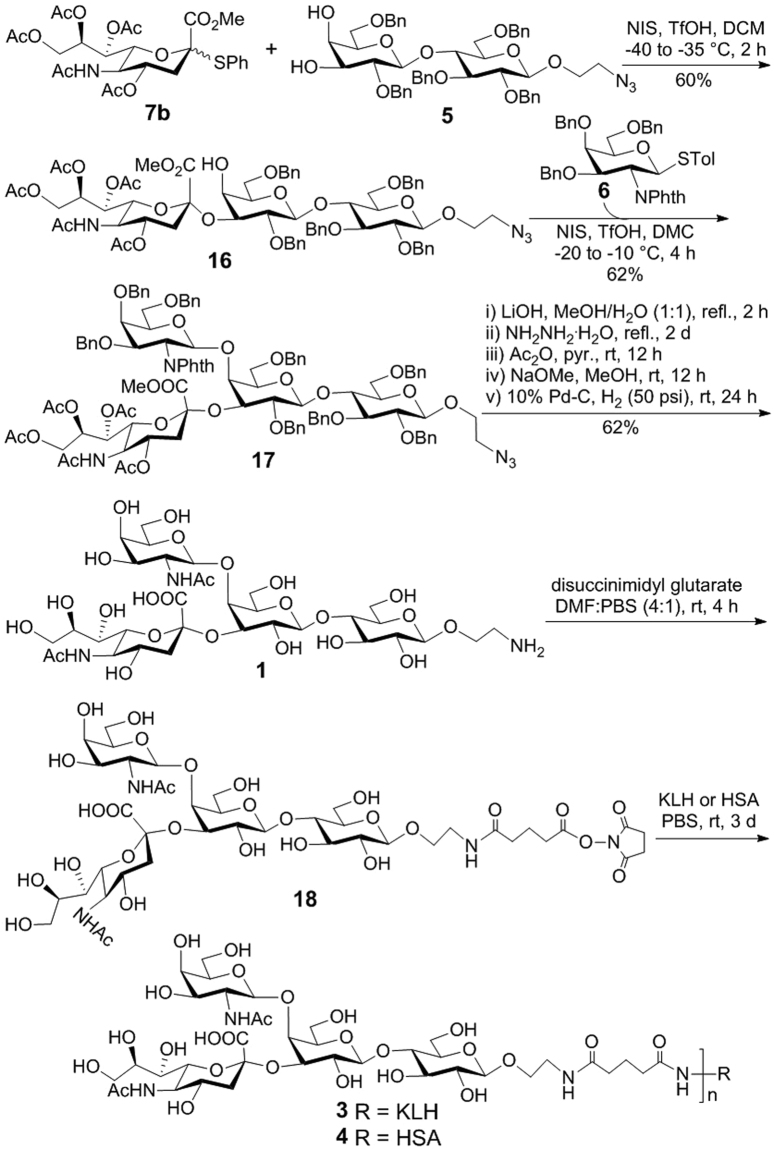
Synthesis of GM2 derivative **1** and its conjugation with KLH and HSA.

At this point, we turned our attention to the sialic acid residue at the galactose 3-*O*-position and wondered whether changing its properties might help improve subsequent 4-*O*-glycosylation and product separation. Fortunately, sialyation of **5** with **7b** ^65, 66^ (Fig. 6) under above conditions gave α-trisaccharide **16** in a good yield (60%) and excellent stereoselectivity (α:β 96:4). Thereafter, NIS-AgOTf mediated glycosylation of **16** with **5** gave significantly improved β-selectivity (α:β 1:6) with the desired $\beta$-tetrasaccharide **17** isolated in a 62% yield. Importantly, the two anomers were easily separated on a silica gel column. The protected GM2 **17** contained several different types of protecting groups such as a methyl carboxylate, a Phth group, an azido group, and a few acetyl and benzyl groups. Therefore, for its successful full deprotection, appropriate reactions in the right order were critical. In this regard, we developed an effective one-pot five-step protocol. First, **17** was treated with LiOH to hydrolyze the methyl carboxylate and remove all acetyl groups. This was followed by the removal of the Phth group upon hydrazine treatment in methanol at reflux. The resultant intermediate was acetylated with acetic anhydride and then selectively de-*O*-acetylated with NaOMe/MeOH. Finally, catalytic debenzylation with concomitant reduction of the azido group was achieved with 10% Pd**-**C under a hydrogen atmosphere. The final product was purified by size exclusion chromatography on a Sephadex G-25 column to afford the target molecule **1** in a good yield (62% for 5 steps).

Compound **1** was then converted into an activated ester for the conjugation with KLH and HSA by an established protocol (Figure 6)^62^. Reaction of **1** with excessive disuccinimidal glutarate (DSG) at room temperature gave **18**. The excessive DSG used in this reaction was removed by washing with ethyl acetate multiple times. The coupling of **18** with KLH and HSA was carried out in 0.1 M PBS buffer, and the resultant glycoconjugates were purified with a Biogel A0.5 column using 0.1 M PBS buffer as the eluent. After dialysis and lyophilization of combined fractions containing the conjugates, **3** and **4** were obtained as white solids. Their carbohydrate loadings were analyzed by a reported method^67^ to be 15 and 12% (w/w), respectively. The carbohydrate loading of the HSA conjugate **4** was also analyzed by MS to give a similar result (13% w/w).

**Figure 7:**
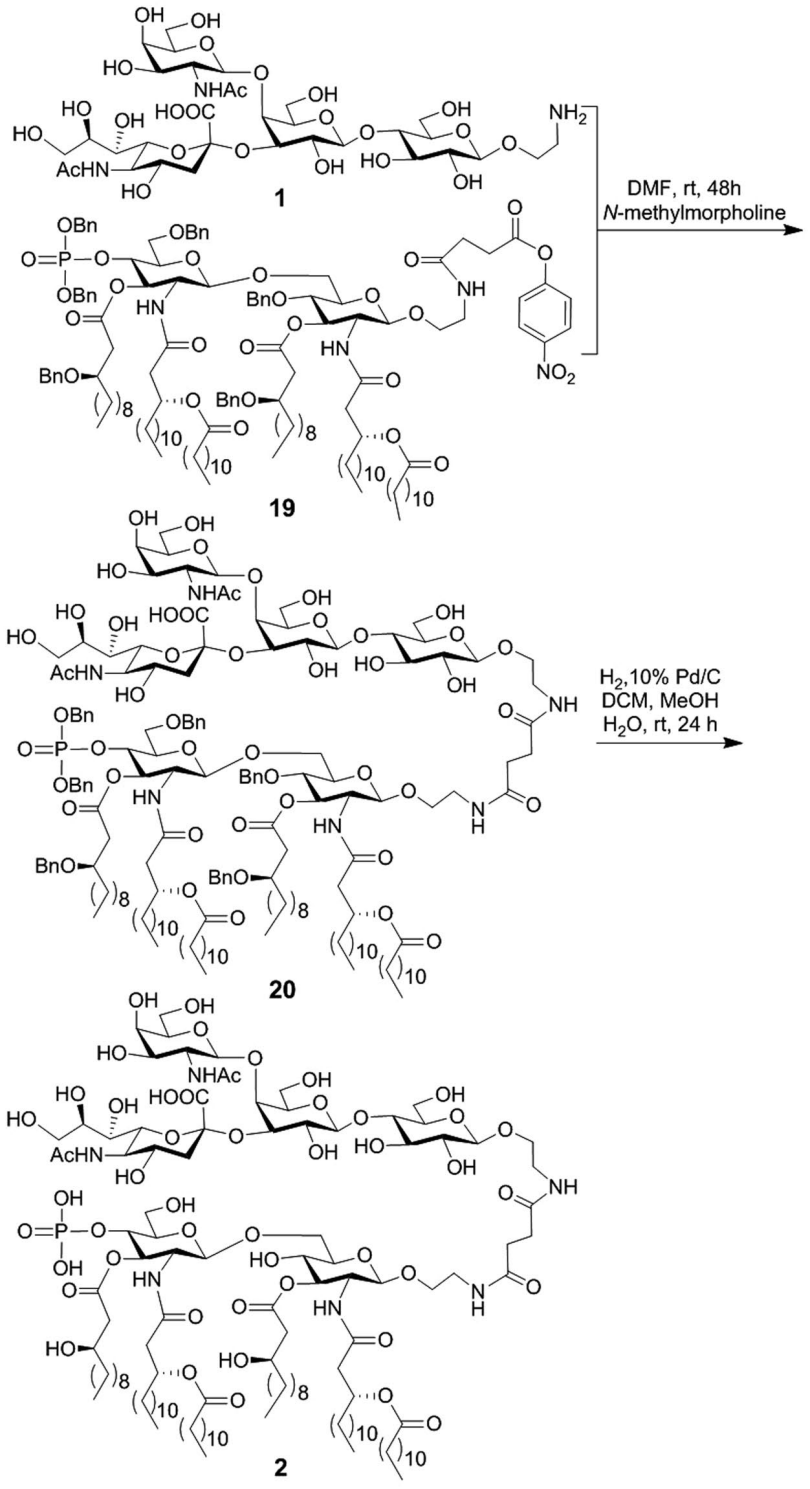
Synthesis of GM2-MPLA conjugate **2**.

Similarly, **1** was conjugated with MPLA via an activated ester of MPLA according to a reported protocol (Fig. 7)^35–38^. The regioselective reaction between **1** and **19** was smooth, yielding **20** that was easily purified. Finally, the product was subjected to catalytic hydrogenolysis to remove the benzyl groups on the MPLA moiety to provide GM2-MPLA conjugate **2**. All synthetic targets and intermediates were fully characterized with NMR and MS.

### Immunological studies of GM2-MPLA and GM2-KLH conjugates

These studies were carried out with female C57BL/6 J mouse. The GM2-MPLA conjugate **2** was administrated in the form of liposomes with 1,2-distearoyl-*sn*-glycero-3-phosphocholine (DSPC) and cholesterol (in molar ratio 10:65:50), prepared by an established protocol^36^. The GM2-KLH conjugate **3** was administrated as emulsions with complete or incomplete Freund’s adjuvant (CFA/IFA). For the initial immunization, a liposomal preparation of **2** or the CFA emulsion of **3** (0.1 mL each, containing GM2 6.0 µg) was injected subcutaneously (s.c.) to a group of six mice. Thereafter, the liposomal preparation of **2** or an IFA emulsion of **3** (0.1 mL) was each injected to the same group of mice on days 14, 21 and 28, respectively, to boost immunization. Blood samples were collected from each mouse on days 21, 28 and 35, and used to prepare antisera by the standard protocol. Finally, the titers of GM2-specific antibodies in each individual antiserum or pooled antiserum were analyzed by enzyme-linked immunosorbent assay (ELISA) with GM2-HSA conjugate **4** as the capture reagent. The optical density (OD) values at 405 nm wavelength were measured and plotted against antiserum dilution numbers to obtain the best-fit line. Based on the equation derived from each curve, antibody titer was calculated and defined as the dilution number that yields an OD value of 0.1.

**Figure 8:**
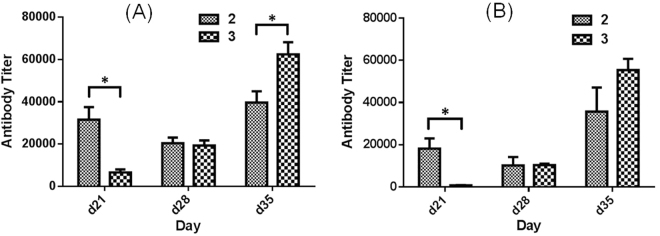
ELISA results of overall total (**A**) and total IgG antibodies (**B**) in days 21, 28, and 35 antisera pooled from mice immunized with GM2-MPLA conjugate **2** or GM2-KLH conjugate **3**, respectively. The average of antibody titers from three parallel experiments for each pooled antiserum is shown, and the error bar represents the standard deviation (SD). *The difference between the results of two conjugates is statistically significant (P < 0.05).

Figure 8 shows the overall total antibody and the total IgG antibody titers of days 21, 28 and 35 antisera pooled from each group of mice immunized with GM2-MPLA conjugate **2** or GM2-KLH conjugate **3**, respectively. Clearly, the titers of the total and the IgG antibodies had a similar trend in each case, but the two conjugates did have different patterns of immune responses. For example, GM2-MPLA conjugate **2** induced a much faster onset of immune responses, which reached a high level after the first boost immunization on day 14 (day 21 antiserum), and the immune response was consistent thereafter. On the other hand, the immune response to GM2-KLH conjugate **3** was low after the first boost immunization but kept growing upon each additional boost immunization. Only after the third boost immunization, GM2-KLH conjugate **3** elicited the similar level of IgG antibodies as GM2-MPLA conjugate **2**, whereas its total antibody titer was slightly higher.

**Figure 9:**
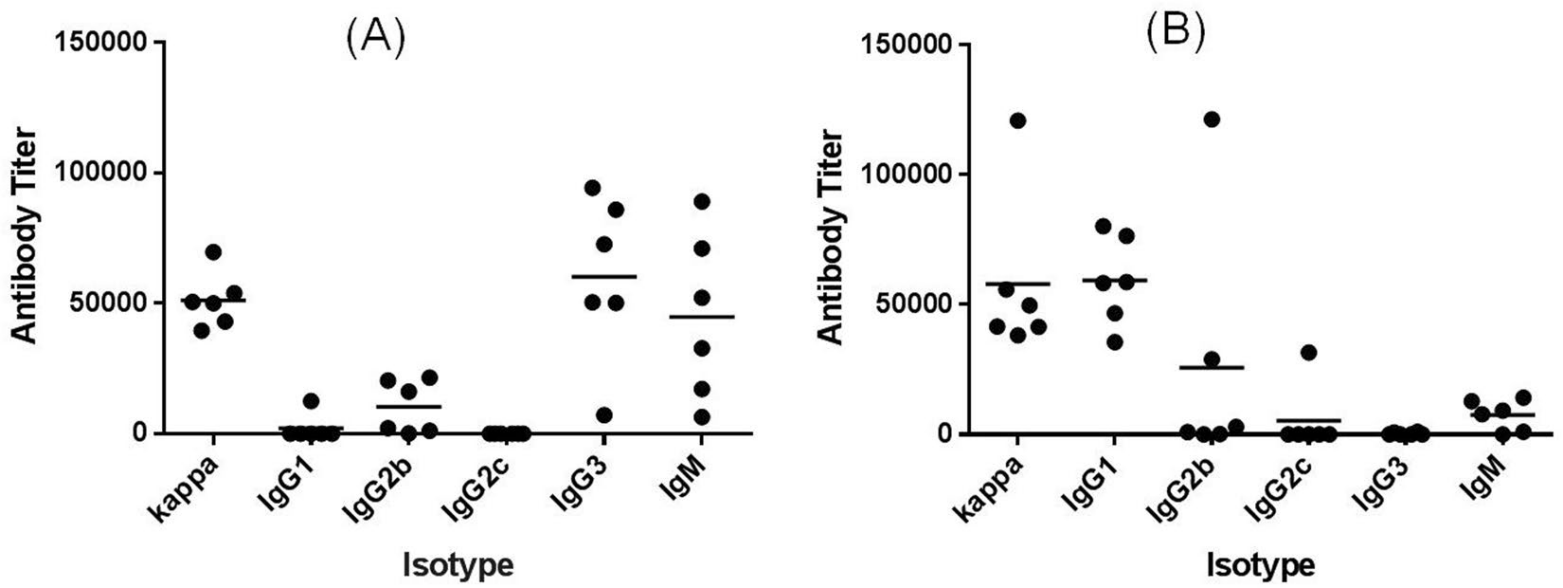
ELISA results of various isotypes of antibodies in the antiserum of individual mouse immunized with (**A**) GM2-MPLA conjugate **2** and (**B**) GM2-KLH conjugate **3**. Each dot represents a mouse, and the horizontal bar represents the average antibody titer of each group of mice.

To gain more insights into the immune responses induced by **2** and **3**, the isotypes of GM2-specific antibodies in the day 35 antiserum of each individual mouse were analyzed by ELISA. As shown in Fig. 9, GM2-MPLA **2** elicited a high level of IgG3 antibody, but GM2-KLH **3** elicited mainly IgG1 antibody. These results were consistent with previous observations^35, 36^ that glycolipids and glycoproteins induced different immunological responses in mice. The two conjugates also elicited low to moderate levels of IgG2b and IgM antibodies. Evidently, the two conjugates might provoke immune responses through different pathways.

### Assessments of antibody binding and antibody-mediated complement-dependent cytotoxicity (CDC) to cancer cells

**Figure 10:**
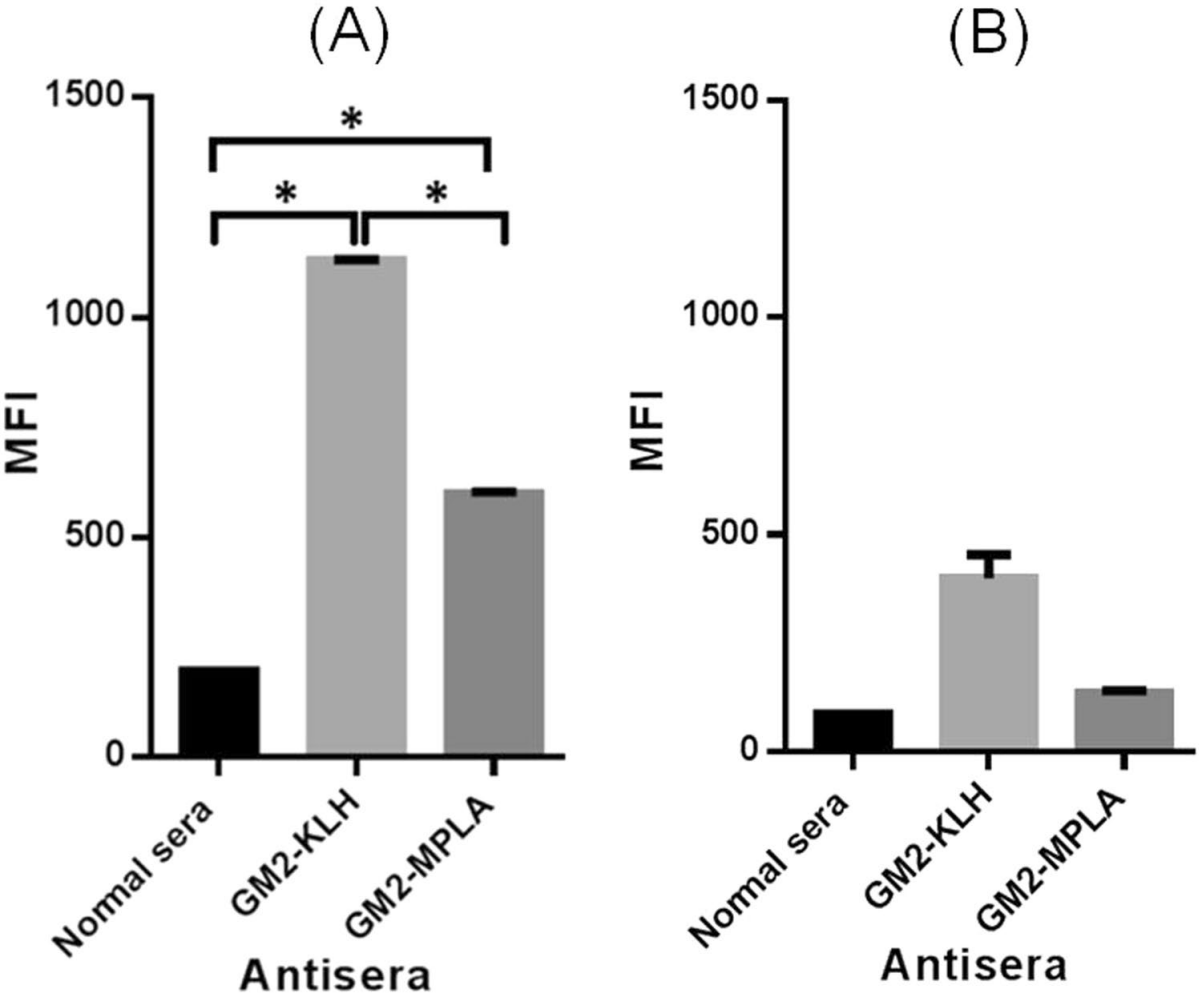
FACS results of the binding of MCF-7 (**A**) or SKMEL-28 (**B**) cells with normal mouse serum or the pooled antisera of GM2-MPLA conjugate **2** and GM2-KLH conjugate **3**. Median fluorescence intensity (MFI) values were used to assess the binding. The error bar represents the SD of two parallel experiments. *The difference between two groups of data is statistically significant (P < 0.05).

As mentioned above, GM2 is expressed by a number of tumors, and the cancer cell line used in this study was MCF-7, a human breast cancer cell line^37, 68, 69^. We studied also the antibody binding and antibody-mediated CDC to SKMEL-28 cell, which was reported to not display GM2 but express structurally closely related GM3 and GD3^70^. The assessments of antibody binding to cancer cells were carried out by the fluorescence-activated cell sorting (FACS) technology. After cancer cells were cultured with the antisera of GM2-MPLA conjugate **2** or GM2-KLH conjugate **3** or with normal mouse serum (the negative control), respectively, the cells were incubated with a DyLight 633-labeled anti-mouse kappa antibody and then subjected to FACS. The results shown in Fig. 10A suggested that antibodies in both antisera showed strong binding to MCF-7 cell, whereas the antiserum of GM2-KLH conjugate **3** had significantly stronger binding to MCF-7 cell than the antiserum of GM2-MPLA conjugate **2**. This finding agreed with the results of antibody titer analyses (Fig. 8). The antisera were also found to have some weak binding to SKMEL-28 cell (Fig. 10B), probably because of certain low-affinity, nonspecific cross-reactions of some antibodies in the antisera with the structurally similar GM3 or GD3 antigens on SKMEL-28 cell, even though for both antisera such binding was not statistically significant. Nevertheless, this study has verified that the antibodies and immune responses elicited by GM2-MPLA conjugate **2** or GM2-KLH conjugate **3** could target GM2 specifically on cancer cells.

To ultimately verify the potential anticancer activities of the induced antibodies, their abilities to mediate CDC to MCF-7 and SKMEL-28 cells were evaluated. For this purpose, cancer cells were first cultured with the antiserum of GM2-MPLA conjugate **2** or GM2-KLH conjugate **3** or a normal mouse serum or an irrelevant monoclonal antibody (negative controls) or without serum at all, and then cultured with rabbit complements. Finally, cell lysis was determined by the lactate dehydrogenase (LDH) assay, with cell lysis rate calculated according to the following equation:

% cell lysis = (experimental A - low control A)/(high control A - low control A) × 100%

where “experimental A” is the OD_490_ value of cells treated with the antiserum, “low control A” is the OD_490_ value of cells cultured without serum, and “high control A” is the OD_490_ value of cells completely lyzed with a 1% triton solution.

**Figure 11:**
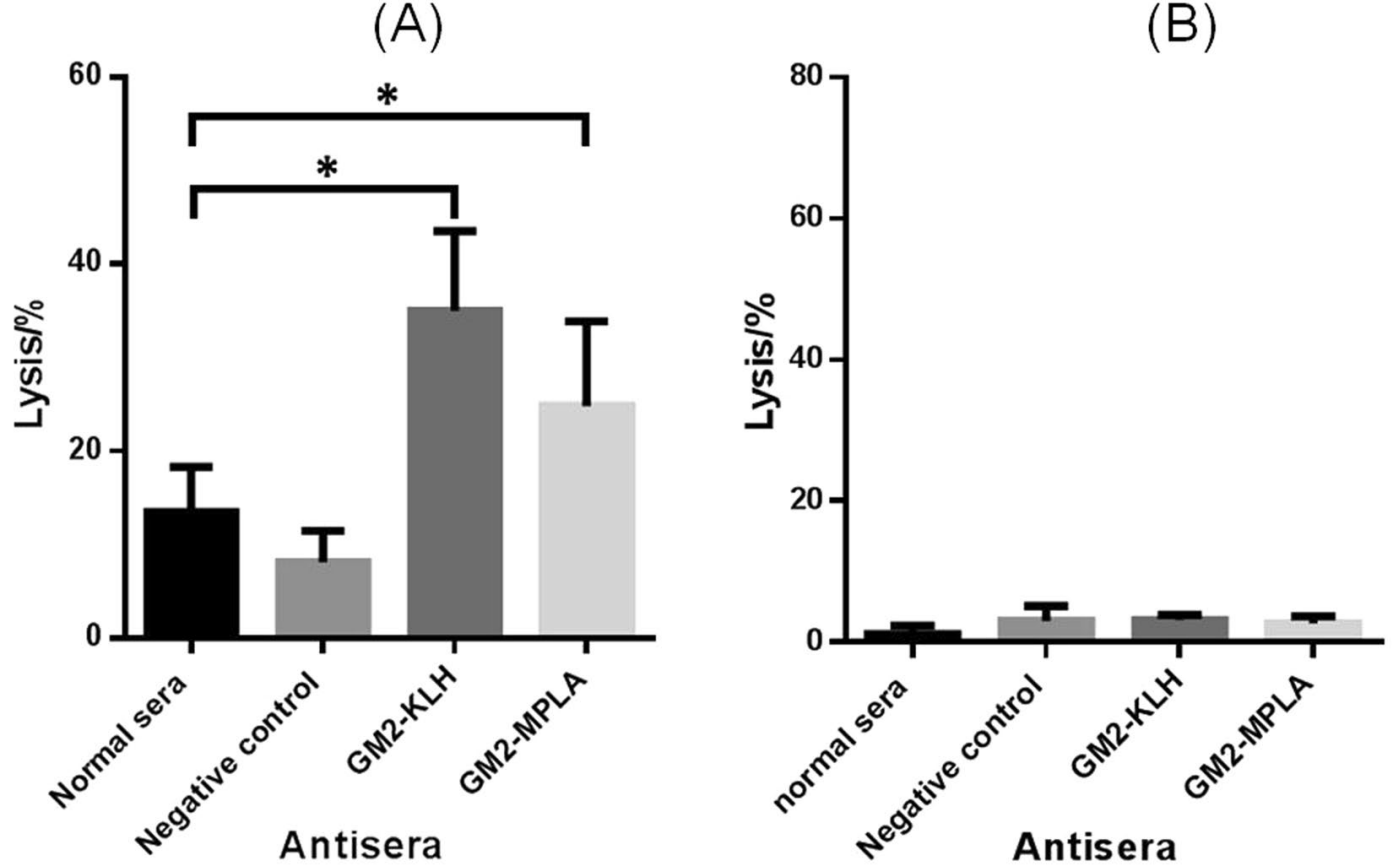
The results of antibody-mediated CDC to MCF-7 (**A**) and SKMEL-28 (**B**) cells, presented as cell lysis rates caused by treatment with rabbit complements and normal mouse serum, irrelevant 2H3 monoclonal antibody as negative control, or pooled antisera of GM2-MPLA conjugate **2** and GM2-KLH conjugate **3**, respectively. The error bar shows the standard deviation of six parallel experiments. *The difference between the results of normal sera and the antisera of GM2-KLH and GM2-MPLA conjugates is statistically significant (P < 0.05).

As shown in Fig. 11A, both antisera mediated significant lysis of MCF-7 cell, as compared to the negative controls, proving that antibodies in these antisera were functional. Interestingly, although the two antisera showed different binding levels to MCF-7 cell, they had similar ability to mediate CDC, i.e., the difference between them was statistically insignificant. This might suggest that so long as there were a sufficient number of antibodies bound to the cancer cell, efficient CDC would be triggered. Additional antibodies beyond necessary may not be useful. On the contrary, despite the weak antibody binding of both antisera to SKMEL-28 cell, neither antiserum induced CDC to this cell line (Fig. 11B). This is an interesting observation that deems more in-depth study. Thus, these results have verified unambiguously that CDCs mediated by antibodies in the antisera of GM2 conjugates **2** and **3** were specific to GM2-positive cancer cells.

In conclusion, an efficient method was developed for the synthesis of a GM2 derivative suitable for simple and effective conjugation with MPLA, proteins or other molecules. Although as mentioned above several GM2 syntheses were described previously, the current synthesis is different in that it used differently protected glycosyl donors and thus involved different synthetic intermediates and glycosylation methods. Moreover, the reactions utilized to achieve the challenging sialyl bond and the branched structure were examined in detail to provide a robust and scalable synthetic strategy. The synthesized GM2 derivative was then coupled with KLH and MPLA to create conjugates that were investigated as therapeutic cancer vaccines. Immunological evaluations in mice revealed that both conjugates elicited robust GM2-specific overall total and total IgG antibody responses. However, it was observed that the MPLA conjugate induced much faster immune responses than the KLH conjugate, whereas the antibody titers induced by the latter were slightly higher than those induced by the former. Among various IgG antibodies induced, IgG3 and IgG1 were the main isotypes for MPLA and KLH conjugates, respectively, which is consistent with the typical patterns of immune responses towards glycolipids and glycoproteins. A major difference between the two conjugates is in the protocol for immunization of mice. The MPLA conjugate was applied alone in the form of liposomes without the use of an external adjuvant, but the KLH conjugate was used as an emulsion with CFA/IFA. This has demonstrated the self-adjuvant property of fully synthetic GM2-MPLA conjugate as a vaccine.

The antisera of both conjugates were proved to contain antibodies that could effectively bind to GM2-positive cancer cells, such as MCF-7, and in agreement with the observed antibody titers, the antiserum of the KLH conjugate had higher binding titers to cancer cells than the antiserum of the MPLA conjugate. Most importantly, however, both antisera had similar activities to mediate efficient CDC to MCF-7. The results eventually verified that the antibodies or immune responses induced by the two conjugates could specifically target and mediate efficient killing of cancer cells that express GM2. Therefore, both glycoconjugates have been shown to be promising therapeutic cancer vaccine candidates. Considering the advantages that fully synthetic glycoconjugate vaccines possess, such as homogeneous and well-defined structure, self-adjuvant property and convenient administration protocol^71^, we are especially interested in GM2-MPLA conjugate **2** as a therapeutic cancer vaccine for further evaluation and study.

## Methods

### General methods

Chemicals and materials were purchased from commercial sources and were used as received without further purification unless otherwise noted. Molecular sieves 4 Å were flame-dried under vacuum and cooled to rt under an N_2_ atmosphere immediately before use. TLC was carried out on Silica Gel 60 Å F254 plates detected by a UV detector and/or by charring with 15% H_2_SO_4_ in EtOH (w/v). Mass spectrometry was recorded on either a high resolution (HR) ESI-TOF or a normal resolution MALD-TOF machine. NMR spectra were recorded on a 600 MHz machine with chemical shifts reported in ppm (*δ*) downfield from internal tetramethylsilane (TMS) or DHO as a reference. Signals are described as s (singlet), d (doublet), t (triplet) or m (multiplet). Coupling constants are reported in Hz. The detailed procedures for the synthesis of GM2 and its various conjugates, as well as all analytical data, are presented as Supplementary Information. CFA, DSPC, and cholesterol were purchased from Sigma-Aldrich. The MCF-7 and SKMEL-28 cancer cell lines, Eagle’s Minimum Essential Medium (EMEM) and Dulbecco’s Modified Eagle’s Medium (DMEM) used for cell culture, and fetal bovine serum (FBS) were purchased from American Type Culture Collection (ATCC). Penicilln-streptomycin and trypsin-EDTA were purchased from Invitrogen. Alkaline phosphatase (AP)-linked goat anti-mouse kappa, IgM, IgG1, IgG2b, IgG2c, and IgG3 antibodies were purchased from Southern Biotechnology. Dylight 633 labeled anti-mouse kappa antibody was purchased from Abcam. Female C57BL/6 mice of 6-8 weeks old used for immunological studies were purchased from the Jackson Laboratories.

### Preparation of vaccine formulations

The liposomal formulation of the GM2-MPLA conjugate **2** was prepared according to a previously reported protocol^36–38^. A mixture of the conjugate (0.57 mg, 0.22 µmol for 30 doses), DSPC, and cholesterol in a molar ratio of 10:65:50 was dissolved in the mixture of CH_2_Cl_2_, MeOH, and H_2_O (3:3:1, v/v). Solvents were removed under reduced pressure at 60 °C through rotary evaporation, resulting in a thin lipid film on the vial wall. The film was hydrated with HEPES buffer (3.0 mL, 20 mM, pH 7.5) containing 150 mM of NaCl, and the mixture was shaken on a vortex mixer. Finally, the mixture was sonicated at rt for 20 min to afford the liposomal formulation of GM2-MPLA conjugate **2**. The CFA or IFA emulsion of GM2-KLH conjugate **3** was prepared by dissolving it (15% loading of GM2, 1.2 mg for 30 doses) in 1.5 mL of 1 × PBS buffer and then thoroughly mixing the solution with CFA or IFA (1.5 mL) according to the manufacturer’s instructions.

### Mouse immunization

Each mouse (female, C57BL/6 J, 6-8 weeks old) was inoculated by s.c. injection of the liposomal formulation of GM2-MPLA conjugate **2** or the CFA/IFA emulsion of GM2-KLH conjugate **3** (0.1 mL) on day 1, day 14, day 21, and day 28. Blood samples were collected from each mouse on day 0 prior to the initial immunization and on day 21, day 27, and day 38 after immunizations. The blood samples were used to obtain antisera, which were stored at -80 °C before immunological analysis. The animal protocol (#A 02-10-14) for this research project was approved by the Institutional Animal Care and Use Committee (IACUC) of Wayne State University, and all animal experiments were carried out in compliance with the relevant laws and institutional guidelines.

### ELISA protocol

ELISAs were performed following the previous protocol. ELISA plates were coated with GM2-HSA conjugate 4 (2 µg/mL) in a buffer (0.1 M bicarbonate, pH 9.6) at 37 °C for 1 h. After washing with PBS buffer containing 0.05% Tween-20 (PBST), the plates were treated with a blocking buffer (1% BSA in PBST) at rt for 1 h. Then, a pooled or an individual mouse serum with serial half-log dilutions from 1:300 to 1:656,100 in PBS (100 µL) was added in each well of the plates, which were then incubated at 37 °C for 2 h. After washing with PBST, a 1:1000 diluted solution of AP enzyme-conjugated goat anti-mouse kappa, IgG1, IgG2b, IgG2c, IgG3 or IgM antibody was added to the plate wells, followed by incubation at rt for 1 h. Finally, the plates were washed and treated with a *p*-nitro-phenylphosphate (PNPP) solution in buffer (1.67 mg/mL, 100 µL per well) at rt for 1 h, followed by reading on a microplate reader at 405 nm wavelength. The antibody titers were calculated from a best-fit logarithm line of the OD_405_ values against the serum dilution numbers. The titers were defined as the dilution number at which an OD_405_ value of 0.1 was achieved.

### FACS assay protocol

The cell samples used for FACS assays were prepared according to the previously reported protocol^37^. MCF-7 and SKMEL-28 cancer cells were incubated in ATCC-formulated EMEM and ATCC-formulated DMEM, both containing 10% FBS and 1% antibiotics. After being treated with trypsin-EDTA solution, the cells were harvested and washed with FACS buffer (PBS containing 5% FBS). The cells were incubated with 50 µL of normal mouse serum (1:10 dilution) or a day 38 pooled antisera (1:10 dilution) at 4 °C for 30 min. Subsequently, the cells were washed and treated with Dylight 633-linked goat anti-mouse kappa antibody (2 µL in 50 µL FACS buffer) at 4 °C for 30 min. The labeled cells were washed, suspended in the FACS buffer, and finally analyzed with a Becton Dickinson LSR II Analyzer.

### Antibody-mediated CDC assay protocol

Antibody-mediated CDC to MCF-7 and SKMEL-28 cells was evaluated by means of the LDH Cytotoxicity Detection Kit according to manufacture’s instructions. MCF-7 and SKMEL-28 cells harvested from the culture medium were seeded in the wells of 96-well plates in concentrations of 1.0 × 10^4^ and 1.5 × 10^4^ cell/well, respectively. After the plates were incubated at 37 °C overnight and washed with the medium without FBS, a normal mouse serum or day 38 antiserum (100 µL, 1:50 dilution in medium without FBS) was added to the wells, and the plates were incubated further at 37 °C for 2 h. Thereafter, the plates were washed, and then a rabbit complement serum solution (100 µL, 1:10 dilution in medium without FBS) was added. The low control (no serum) and the high control (adding 100 µL of 1% triton-100 instead of the serum) were set at the same time. The negative control was cell samples incubated with the 2H3 monoclonal antibody (100 µL, 1 µg/ml in PBS), which was specific to an unnatural GM3 analog^72^. After incubation at 37 °C for 1 h, the supernatants of each plate (20 µL from each well) were carefully relocated to another 96-well plate containing 80 µL of PBS buffer in each well. The latter was then developed with the LDH cytotoxicity detection reagent, and after 1 h of incubation the absorbance at 490 nm wavelength was examined with a plate reader. The percentage of cell lysis is calculated according to the following equation:

Cell lysis (%) = (experimental A – low control A)/(high control A – low control A) × 100%

where “experimental A” is the OD_490_ value of cells treated with an antiserum, “low control A” is the OD_490_ value of cells cultured without serum, and “high control A” is the OD_490_ value of cells completely lysed with the 1% triton-100 solution.

## Electronic supplementary material

Supplementary Information
