# Supplementary material for: Synthesis and Evaluation of GM2-Monophosphoryl Lipid A Conjugate as a Fully Synthetic Self-Adjuvant Cancer Vaccine
Source: Sci Rep. 2017 Sep 12;7:11403. doi: 10.1038/s41598-017-11500-w (PMC5595996; doi:10.1038/s41598-017-11500-w)
Supplement: Supplementary file 1 — Supplementary Information [file 41598_2017_11500_MOESM1_ESM.pdf]

Supporting Information for

Synthesis and Evaluation of GM2-Monophosphoryl Lipid A Conjugate as a Fully Synthetic Self-Adjuvant Cancer Vaccine

Zhifang Zhou,[a,†] Satadru Mandal,[a,†] Guochao Liao,[a,b,†] Jiatong Guo,[a,c] and Zhongwu Guo[a,c,*]

[a] Department of Chemistry, Wayne State University, 1501 Cass Avenue, Detroit, Michigan 48202, United States

[b] International Institute for Translational Chinese Medicine, Guangzhou University of Chinese Medicine, 232 Waihuan Donglu, Guangdong 510006, China

[c] Department of Chemistry, University of Florida, 214 Leigh Hall, Gainesville, Florida 32611, United States

Table of contents

I. Synthesis and Analysis of GM2 conjugates-- Page S2-S10

II. Raw ELISA Data and Additional Data-- Page S10-S12

III. NMR and MS Spectra of the Synthetic Intermediates and Final Products------- Page S13-S46

I. Synthesis and Analysis of GM2 conjugates

Compound 9. To a solution of **8** (8.0 g, 19.4 mmol) dissolved in anhydrous MeCN were added DMP (2.88 mL, 23.3 mmol) and CSA (1.13 g, 4.8 mmol). The reaction was kept at rt until TLC showed its completion, and was then quenched with Et_3N (0.7 mL, 4.8 mmol) and diluted with CH_2Cl_2 (90 mL). The mixture was washed with brine, dried over Na_2SO_4, and concentrated in vacuum. The residue was purified by flash column chromatography ($MeOH/CH_2Cl_2$, 1:10, v/v) to afford **9** as a white solid (6.12 g, 72%). ^{1}H NMR (600 MHz, $CDCl_3$): δ: 5.22 (bs, 1-OH), 5.04 (bs, 1-OH), 4.51 (d, *J* = 8.1 Hz, 1 H, H-1), 4.35 (d, *J* = 7.3 Hz, 1 H, H-1'), 4.16 (bs, 1-OH), 4.09-4.03 (m, 3 H, H-3, H-6, H-6'), 3.94-3.87 (m, 3 H, H-2), 3.86-3.78 (m, 3H), 3.75-3.62 (m, 4H), 3.56-3.34 (m, 5H, 1-OH), 1.46 (s, 6 H); ^{13}C NMR (150 MHz, $CDCl_3$): δ: 102.7, 102.5, 99.4, 75.3, 75.2, 73.4, 72.1, 69.0, 68.4, 68.2, 66.8, 62.5, 61.2, 50.8, 21.4, 18.4; ESI-TOF HR-MS *m/z*: calcd. for $C_{17}H_{29}N_3O_{11}$ $[M + Na]^+$ 474.4; found 474.5.

Compound 10. A mixture of **9** (6.0 g, 13.4 mmol) and NaH (2.2 g, 93.46 mmol) in anhydrous DMF (30 mL) was stirred at 0 ^{o}C for 45 min, and then to it was added benzyl bromide (11.1 mL, 93.46 mmol). The mixture was stirred at 0 ^{o}C for another 6 h, when TLC showed the completion of reaction. The reaction was quenched with H_2O at 0 ^{o}C, and the mixture was diluted with EtOAc. The aqueous layer was washed with EtOAc (15 x 5 mL), and the organic phase was combined, dried over Na_2SO_4, and concentrated. The residue was purified by flash column chromatography (acetone/hexane 2:10, v/v) to give **10** (9.71 g, 81%) as colorless syrup. ^{1}H NMR (400 MHz, $CDCl_3$): δ 7.56-7.51 (m, 2 H, ArH), 7.44-7.16 (m, 23 H, ArH), 5.19 (d, *J* = 10.8 Hz, 1 H), 4.94 (d, *J* = 11.7 Hz, 1 H), 4.91 (d, *J* = 11.7 Hz, 1 H), 4.82 (d, *J* = 11.7 Hz, 1 H), 4.78 (d, *J* = 11.7 Hz, 1 H), 4.75-4.68 (m, 3 H), 4.55 (d, *J* = 12.7 Hz, 1 H), 4.47-4.41 (m, 2 H, H-1, H-1'), 4.36 (d, *J* = 12.7 Hz, 1 H), 4.10-3.92 (m, 3 H), 3.91-3.81 (m, 3 H), 3.79-3.61 (m, 4 H), 3.56-3.30 (m, 5 H), 2.86 (s, 1 H), 1.52 (s, 3 H), 1.44 (s, 3 H); ^{13}C NMR (100 MHz, $CDCl_3$): δ 139.0, 138.9, 138.7, 138.4, 138.3, 129.1, 128.7, 128.4, 128.3, 128.28, 128.25, 128.22, 128.1, 127.9, 127.8, 127.76, 127.6, 127.5, 127.4, 125.3, 103.7, 102.6, 98.8, 82.9, 81.8, 79.8, 78.9, 77.4, 75.7, 75.3, 75.2, 75.1, 73.0, 71.8, 68.3, 68.1, 66.4, 66.2, 62.5, 51.0, 29.2, 18.8; ESI-TOF HR-MS (positive mode) *m/z*: calcd. for $C_{52}H_{59}N_3O_{11}$ $[M + Na]^+$ 925.1; found 925.0.

Compound 5. To a solution of **10** (5.0 g, 5.5 mmol) in MeOH was added HCl in MeOH (5%, 0.2 mL). The solution was stirred at rt until TLC showed the completion of reaction. After the reaction was quenched with Et_3N, the mixture was diluted with CH_2Cl_2 (80 mL), washed with brine, dried over anhydrous Na_2SO_4, and concentrated in vacuum. The residue was purified by flash column chromatography (acetone/CH_2Cl_2, 2:10, v/v) to give **5** as a white solid (3.91 g, 83%). ^{1}H NMR (600 MHz, $CDCl_3$): δ 7.42-7.39 (m, 2 H, ArH), 7.37-7.20 (m, 23 H, ArH), 4.98 (d, *J* = 10.3 Hz, 1 H), 4.92 (d, *J* = 11.0 Hz, 1 H), 4.81-4.72 (m, 4 H), 4.68 (ABq, *J* = 11.7, 7.3 Hz, 2 H), 4.54 (d, *J* = 11.7 Hz, 1 H), 4.42 (d, *J* = 8.0 Hz, 1 H, H-1), 4.38 (d, *J* = 11.0 Hz, 1 H), 4.36 (d, *J* = 7.4 Hz, 1 H, H-1'), 4.06-4.00 (m, 1 H), 3.92 (t, *J* = 9.5 Hz, 1 H), 3.90-3.87 (m, 1 H), 3.79 (dd, *J* = 11.0, 4.4 Hz, 1 H), 3.74-3.68 (m, 2 H), 3.65-3.53 (m, 4 H), 3.52-3.37 (m, 4 H), 3.33 (dd, *J* = 9.5, 2.9 Hz, 1 H), 3.17-3.13 (m, 1 H), 2.65 (bs, 1-OH); ^{13}C NMR (150 MHz, $CDCl_3$): δ 138.8, 138.6, 138.5, 138.2, 137.8, 128.5, 128.4, 128.5, 128.4, 128.3, 128.27, 128.2,128.1, 128.0, 127.9, 127.8, 127.79, 127.77, 127.75, 127.6, 127.56, 103.6, 102.6, 82.7, 82.6, 81., 79.2, 76.7, 75.6, 75.3, 75.1,75.0, 74.0, 73.2, 72.1, 68.1, 67.2,62.3, 51.0; ESI-TOF HR-MS *m/z*: calcd. for $C_{49}H_{55}N_3O_{11}$ $[M + Na]^+$ 884.3734; found 884.3729.

Compound 6. A suspension of D-galactosamine hydrochloride **11** (5 g, 23.25 mmol), Na_2CO_3 (2.46 g 23.25 mmol), and phthalic anhydride (3.45 g, 23.25 mmol) in H_2O (50 ml) was stirred at rt for 3 h. The solution was lyophilized to give a pale yellow solid residue, which was suspended in pyridine (200 ml) at 0 ^{o}C, and then acetic anhydride (100 ml) was added. The suspension was stirred at rt for 20 h. Acetic anhydride and pyridine were evaporated in vacuum, and the residue was co-evaporated with toluene (10 ml) twice and then subjected to silica gel chromatography (EtOAc/toluene 1:9, v/v) to give **12** as a white solid (8.18 g, 74%). To the stirred solution of **12** (5.0 g, 10.47 mmol) and *p*-toluenethiol (1.95 g, 15.71 mmol) in anhydrous CH_2Cl_2 (30 mL) was added $BF_3{\cdot}Et_2O$ (1.6 mL, 12.56 mmol) dropwise at 0 ^{o}C. When TLC showed the completion of reaction, the mixture was washed with saturated aq. $NaHCO_3$ solution and brine, dried over anhydrous Na_2SO_4, and concentrated. The residue was purified by flash column chromatography (EtOAc/hexane 1:4, v/v) to give **13** (spectroscopic data were identical to that of the reported)[58] as a white solid (4.96 g, 88%). To a stirred solution of **13** (4.9 g, 9.05 mmol) in MeOH (25 mL) was added CH_3ONa in CH_3OH (0.4 M) until pH reached 9.5. The mixture was stirred for another 4 h. When TLC showed the completion of reaction, it was neutralized with Amberlyst (H^+) resin to pH

6-7, concentrated in vacuum, and purified by flash column chromatography ($MeOH/CH_2Cl_2$, 1:8, v/v) to afford a solid compound (3.43 g, 91%) that was directly applied to the next reaction. The solid product (3.0 g, 7.2 mmol) was dissolved in anhydrous DMF, and to the solution was added NaH (867 mg, 36.14 mmol) at 0 °C. After 45 min of stirring, benzyl bromide (4.3 mL, 36.14 mmol) was added at 0 °C, and the mixture was stirred for another 6 h. When TLC showed the completion of reaction, it was quenched with H_2O, and the mixture was diluted with EtOAc. The aqueous layer was extracted with EtOAc (5 x 20 mL), and the combined organic layer was dried over Na_2SO_4 and concentrated. The residue was purified by flash column chromatography (acetone/hexane 1:11, v/v) to yield **5** as a white solid (4.38 g, 83%). ^{1}H NMR (600 MHz, $CDCl_3$): δ 7.89-7.84 (m, 2 H, ArH), 7.75-7.64 (m, 3 H, ArH), 7.39-7.27 (m, 12 H, ArH), 7.08-6.94 (m, 7 H, ArH), 5.53 (d, J = 10.3 Hz, 1 H, H-1), 4.99 (d, J = 11.7 Hz, 1 H), 4.84 (t, J = 10.3 Hz, 1 H, H-2), 4.63 (d, J = 10.3 Hz, 1 H), 4.60 (d, J = 11.0 Hz, 1 H), 4.47 (ABq, J = 11.7 Hz, 2 H), 4.36 (d, J = 10.3 Hz, 1 H, H-3), 4.31 (d, J = 11.7 Hz, 1 H), 4.10 (s, 1H, H-4), 3.83-3.79 (m, 1 H, H-5), 3.73-3.69 (m, 2 H, H-6, H-6'), 2.26 (s, 3 H); ^{13}C NMR (150 MHz, $CDCl_3$): δ 168.4, 167.5, 138.6, 138., 137.6, 134.0, 133.8, 132.6, 131.8, 129.5, 129.2, 128.4, 128.2, 128.1, 127.9, 127.8, 127.7, 127.6, 127.5, 123.5, 123.1, 84.5, 77.6, 77.5, 74.5, 73.5, 72.2, 71.4, 68.9, 51.8, 21.1; ESI-TOF HR-MS *m/z*: calcd. for $C_{42}H_{39}NO_6S$ $[M + Na]^+$ 708.2396; found 708.2390.

Compound 14. A mixture of **5** (1.5 g, 1.74 mmol) and **7a** (2.19 g, 3.48 mmol) was azeotroped twice with anhydrous toluene (5 mL) and dried under high vacuum for 5 h. It was then dissolved in dry CH_3CN/CH_2Cl_2 (8:2, 20 mL), mixed with freshly activated 4Å molecular sieves (3 g), and stirred under an Ar atmosphere at rt for 1 h. To the mixture was added NIS (1.17 g, 5.22 mmol). After cooling to -30 ºC, TfOH (15.39 μL, 0.174 mmol) was added, and the mixture was stirred at -20 ºC for 2 h. When TLC showed the completion of reaction, saturated aq. $NaHCO_3$ and CH_2Cl_2 were added, and the mixture was filtered through a Celite pad to remove molecular sieves. After extraction of the aqueous layer with CH_2Cl_2 (3 x 15), the combined organic phase was dried over Na_2SO_4 and concentrated in vacuum. The residue was purified by flash column chromatography (acetone/hexane 5:10, v/v) to give **14** (1.78 g, 74%) as syrup and a small amount of the β isomer (8%). ^{1}H NMR (600 MHz, $CDCl_3$): δ 7.43-7.40 (m, 2 H, ArH), 7.36-7.22 (m, 22 H, ArH), 7.19-7.15 (m, 1 H, ArH), 5.57-5.54 (m, 1 H, H-8c), 5.35 (s, 1 H, -Sialic NH), 5.15 (dd, J = 9.5, 1.5 Hz, 1 H, H-7c), 4.93 (d, J = 11.0 Hz, 1 H), 4.90 (d, J = 11.0 Hz, 1 H), 4.79 (d, J = 11.0 Hz, 1 H), 4.75 (d,

J = 11.0 Hz, 1 H), 4.73 (d, *J* = 11.0 Hz, 1 H), 4.72 (d, *J* = 11.0 Hz, 1 H), 4.68 (d, *J* = 11.7 Hz, 1 H), 4.55 (d, *J* = 11.7 Hz, 1 H), 4.49 (d, *J* = 12.5 Hz, 1 H), 4.48 (d, *J* = 7.3 Hz, 1 H, H-1'), 4.43 (d, *J* = 7.3 Hz, 1 H, H-1), 4.42 (d, *J* = 11.7 Hz, 1 H), 4.32 (dd, *J* = 12.5, 3.6 Hz, 1 H), 4.26 (d, *J* = 14.1 Hz, 1 H), 4.22-4.20 (m, 1 H, H-4c), 4.14 (d, *J* = 14.1 Hz, 1 H), 4.01 (s, 2 H), 4.05-4.00 (m, 4 H), 3.99-3.88 (m, 3 H), 3.82-3.77 (m, 3 H), 3.76 (s, 3 H), 3.74-3.68 (m, 3 H), 3.62-3.54 (m, 3 H), 3.52-3.36 (m, 5 H), 3.28 (t, J = 6.6 Hz, 1 H), 2.91 (t, J = 10.3 Hz, 1 H), 2.75 (dd, *J* = 12.5, 3.6 Hz, 1 H, H-3eq-c), 2.03 (t, J = 12.5 Hz, 1 H, H-3ax-c), 1.77 (bs, 1 OH); ^{13}C NMR (150 MHz, $CDCl_3$): δ 168.2, 168.0, 167.0, 166.2, 159.1, 139.3, 138.52, 138.50, 138.2, 137.9, 129.0, 128.5, 128.4, 128.3, 128.2, 128.1, 128.05, 128.0, 127.9, 127.8, 127.7, 127.6, 127.5, 127.2, 125.3, 103.6, 102.2, 100.2, 82.6, 81.8, 80.9, 79.2, 76.7, 76.0, 75.2, 75.0, 74.9, 73.5, 73.2, 72.2, 72.1, 70.4, 68.4, 68.2, 65.6, 63.2, 62.9, 57.4, 53.4, 51.0, 40.9, 40.4, 40.3, 36.1; ESI-TOF HR-MS *m/z*: calcd. for $C_{66}H_{73}Cl_3N_4O_{22}$ $[M + Na]^+$ 1401.3680; found 1401.3647.

Compound 15. A mixture of **14** (0.50 g, 0.362 mmol) and **6** (0.50 g, 0.724 mmol) was azeotroped twice with anhydrous toluene (5 mL) and then dried under high vacuum for 5 h. The mixture was dissolved in CH_2Cl_2 (10 mL), combined with freshly activated 4Å molecular sieves (3 g), and stirred under an Ar atmosphere at rt for 1 h. To the mixture was added NIS (162 mg, 0.724 mmol). After cooling to -20 ºC, TfOH (16 μL, 0.181 mmol) was added, and the reaction was stirred at -10 ºC for 2 h. After TLC showed the completion of reaction, CH_2Cl_2 and saturated aq. $NaHCO_3$ solution were added. The resulting mixture was filtered through a Celite pad to remove molecular sieves. The water layer was extracted with CH_2Cl_2 (3 x 10), and the combined organic phase was dried over Na_2SO_4 and concentrated in vacuum. The residue was purified by silica gel column chromatography (acetone/hexane 1:11, v/v) to afford an anomeric mixture **15** (α:β 1:2) (455 mg, 65%) as syrup. The β isomer: ^{1}H NMR (600 MHz, $CDCl_3$): δ 7.79-7.76 (m, 1 H, ArH), 7.54-7.50 (m, 2 H, ArH), 7.49-7.46 (m, 2 H, ArH), 7.44-7.14 (m, 32 H, ArH), 7.07-7.04 (m, 2 H, ArH), 7.01-6.96 (m, 2 H, ArH), 6.95-6.92 (m, 2 H, ArH), 6.76-6.71 (m, 1 H, ArH), 5.39-5.36 (m, 1 H, H-8c), 5.23 (d, *J* = 6.9 Hz, 1 H, H-1'''), 5.35 (s, 1 H, -Sialic NH), 5.00-4.97 (m, 1H, H-7c, *J* = 11.7 Hz, 1 H), 4.91-4.87 (m, 2 H), 4.72-4.69 (m, 2 H), 4.67-4.63 (m, 3 H), 4.59 (d, *J* = 11.7 Hz, 1 H), 4.54 (d, *J* = 12.5 Hz, 1 H), 4.49 (d, *J* = 12.5 Hz, 1 H), 4.45 (d, *J* = 12.5 Hz, 1 H), 4.43 (d, *J* = 12.5 Hz, 1 H), 4.39 (d, *J* = 6.9 Hz, 1 H, H-1), 4.36 (d, *J* = 12.5 Hz, 1 H), 4.34 (d, *J* = 12.5 Hz, 1 H), 4.29 (d, *J* = 12.5 Hz, 1 H), 4.18 (d, *J* = 7.3 Hz, 1 H, H-1'), 4.16-4.13 (m, 4 H), 4.09 (d, *J* = 12.5 Hz, 1 H), 4.06

(d, J = 12.5 Hz, 1 H), 4.02-3.96 (m, 4 H), 3.92 (d, J = 10.1 Hz, 1 H), 3.87 (d, J = 13.6 Hz, 1 H), 3.83 (d, J = 10.1 Hz, 1 H), 3.80-3.72 (m, 3 H), 3.71-3.67 (m, 2 H), 3.66-3.55 (m, 5 H), 3.53 (s, 3 H), 3.50-3.44 (m, 4 H), 3.41-3.36 (m, 1 H), 3.35-3.30 (m, 1 H), 3.29-3.24 (m, 1 H), 3.07-2.96 (m, 3 H), 2.48 (dd, J = 12.5, 3.0 Hz, 1 H, H-3eq-c), 1.90 (t, J = 12.5 Hz, 1 H, H-3ax-c)**;** ^{13}C NMR (150 MHz, $CDCl_3$): δ 168.7, 168.1, 167.8, 167.7, 166.9, 166.0, 159.2, 138.9, 138.7, 138.5, 138.4, 138.3, 138.01, 137.97, 137.88, 133.4, 132.6, 131.8, 129.9, 129.0, 128.5, 128.3, 127.7, 127.4, 126.9, 123.2, 122.7, 103.7, 101.4, 100.5, 99.7, 82.3, 81.8, 80.3, 79.9, 76.9, 75.6, 75.3, 74.5, 73.5, 73.1, 72.9, 72.4, 72.0, 70.5, 68.9, 68.5, 68.2, 64.6, 62.8, 56.8, 53.3, 53.1, 50.9, 40.7, 40.4, 40.3, 35.4; ESI-TOF HR-MS *m/z*: calcd. for $C_{101}H_{104}N_5O_{28}$ $[M + Na]^+$ 1962.5831; found 1962.5844.

Compound 16. A mixture of **5** (1.5 g, 1.74 mmol) and **7b** (2.03 g, 3.48 mmol) was azeotroped twice with anhydrous toluene (5 mL) and dried under high vacuum for 5 h. The mixture was then dissolved in CH_3CN/CH_2Cl_2 (4:1, 30 mL), combined with freshly activated 4Å molecular sieves (3 g), and stirred under an Ar atmosphere at rt for 1 h. To the mixture was added NIS (783 mg, 3.48 mmol). After cooling to -20 ºC, TfOH (15 μL, 0.174 mmol) was added, and the mixture was stirred at -15 ºC for 2 h. When TLC showed the completion of reaction, CH_2Cl_2 and saturated aq. $NaHCO_3$ solution were added, and the resulting mixture was filtered through a Celite pad to remove molecular sieves. The aqueous layer was extracted with CH_2Cl_2 (3 x 10). The combined organic phase was dried over Na_2SO_4 and concentrated in vacuum, and the residue was purified by flash column chromatography (EtOAc/toluene 2:10, v/v) to give **16** (1.39 g, 60%) as syrup, as well as a very small amount of its β isomer (2.5%). Compound **16**: ^{1}H NMR (600 MHz, $CDCl_3$): δ 7.43-7.40 (m, 1 H, ArH), 7.38-7.20 (m, 24 H, ArH), 5.38-5.33 (m, 2 H, -Sialic NH, H-8c), 5.21 (dd, J = 9.5, 2.2 Hz, 1 H, H-7c), 4.97 (d, J = 11.0 Hz, 1 H), 4.89 (d, J = 11.0 Hz, 1 H), 4.87-4.83 (m, 1 H, H-4c), 4.78 (d, J = 11.0 Hz, 2 H), 4.72 (d, J = 11.0 Hz, 2 H), 4.64 (d, J = 11.7 Hz, 1 H), 4.49 (d, J = 11.7 Hz, 1 H), 4.45 (d, J = 8.1 Hz, 1 H, H-1'), 4.41 (d, J = 7.3 Hz, 1 H, H-1), 4.38 (d, J = 12.4 Hz, 1 H), 4.36-4.34 (m, 1 H), 4.11 (d, J = 10.4 Hz, 1 H), 4.09-4.00 (m, 3 H), 3.97 (t, J = 8.8 Hz, 1 H, H-3), 3.77 (s, 3 H), 3.79-3.66 (m, 5 H), 3.63-3.55 (m, 3 H), 3.51-3.49 (m, 1 H), 3.45-3.36 (m, 3 H), 3.29-3.25 (m, 1 H), 2.45 (dd, J = 12.5, 4.4 Hz, 1 H, H-3eq-c), 2.11, 2.10, 2.03, 1.94, 1.88 (5s, 15 H), 1.89 (t, 1 H, H-3ax-c); ^{13}C NMR (150 MHz, $CDCl_3$): δ 170.9, 170.8, 170.2, 170.1, 170.0, 167.9, 139.1, 138.7, 138.6, 138.3, 138.2, 128.4, 128.34, 128.28, 128.27, 128.21, 128.11, 128.1, 127.9, 127.8, 127.7, 127.6, 127.52, 127.48, 127.4, 103.6, 102.5, 98.9, 82.5, 81.8, 81.2, 79.3,

76.7, 75.2, 75.1, 75.0, 74.9, 73.1, 72.1, 71.6, 69.11, 69.1, 68.4, 68.1, 67.5, 64.9, 62.4, 61.8, 52.9, 50.9, 49.4, 36.4, 23.2, 21.1, 20.8, 20.7; ESI-TOF HR-MS *m/z*: calcd. for $C_{69}H_{82}N_4O_{23}$ $[M + H]^+$ 1335.5440; found 1335.5448.

Compound 17. A mixture of **16** (0.50 g, 0.374 mmol) and **6** (0.77 g, 1.12 mmol) was azeotroped twice with anhydrous toluene (5 mL) and then dried under high vacuum for 5 h. The mixture was dissolved in CH_2Cl_2 (20 mL), combined with freshly activated 4Å molecular sieves (3 g), and stirred under an Ar atmosphere at rt for 1 h. To the mixture was added NIS (253 mg, 1.12 mmol). After cooling to -40 ºC, TfOH (3.31 μL, 0.04 mmol) was added, and the reaction was stirred at -30 ºC for 2 h. When TLC showed the completion of reaction, CH_2Cl_2 and saturated aq. $NaHCO_3$ solution were added, and the mixture was filtered through a Celite pad to remove molecular sieves. After the aqueous layer was extracted with CH_2Cl_2 (3 x 10), the combined organic phase was dried over Na_2SO_4 and condensed in vacuum. The residue was purified by silica gel column chromatography (acetone/hexane 1:5, v/v) to give **17** (440 mg, 62%) as syrup, as well as a small amount of its α isomer (10%). Compound **17**: ^{1}H NMR (600 MHz, $CDCl_3$): δ 7.77-7.75 (m, 1 H, ArH), 7.54-7.45 (m, 4 H, ArH), 7.41-7.30 (m, 14 H, ArH), 7.30-7.15 (m, 18 H, ArH), 7.08-7.05 (m, 2 H, ArH), 7.03-6.98 (m, 2 H), 6.96-6.93 (m, 2 H), 6.89-6.85 (m, 1 H), 5.25-5.22 (m, 1 H, H-8c, *J* = 8.1 Hz, 1 H, H-1’’’), 5.20-5.14 (m, 2 H, -Sialic NH, H-7c), 5.21 (dd, *J* = 9.5, 2.2 Hz, 1 H, H-7c), 4.95 (d, *J* = 11.0 Hz, 2 H), 4.88 (d, *J* = 11.0 Hz, 1 H), 4.85-4.80 (m, 1 H), 4.78 (d, *J* = 11.0 Hz, 1 H), 4.69 (d, *J* = 12.5 Hz, 1 H), 4.67 (d, *J* = 12.5 Hz, 1 H), 4.63 (d, *J* = 12.5 Hz, 2 H), 4.57 (d, *J* = 11.7 Hz, 1 H), 4.56 (d, *J* = 11.0 Hz, 1 H), 4.50 (d, *J* = 11.7 Hz, 1 H), 4.49 (d, *J* = 12.5 Hz, 1 H), 4.42 (d, *J* = 12.5 Hz, 1 H), 4.37 (d, *J* = 12.5 Hz, 1 H), 4.36 (d, *J* = 7.3 Hz, 1 H, H-1), 4.29 (d, *J* = 11.7 Hz, 1 H), 4.28 (d, *J* = 12.5 Hz, 1 H), 4.24 (d, *J* = 7.3 Hz, 1 H, H-1’), 4.20 (dd, *J* = 12.5, 2.9 Hz, 1 H), 4.13-4.10 (m, 2 H), 4.02-3.92 (m, 4 H), 3.85-3.80 (m, 2 H), 3.78-3.74 (m, 2 H), 3.69-3.55 (m, 5 H), 3.54 (s, 3 H), 3.53-3.31 (m, 7 H), 3.15-3.09 (m, 2 H), 3.03-2.99 (m, 1 H), 2.43 (dd, *J* = 12.5, 4.4 Hz, 1 H, H-3eq-c), 2.01, 1.99, 1.98, 1.90, 1.84 (5s, 15 H), 1.76 (t, *J* = 12.5 Hz, 1 H, H-3ax-c); ^{13}C NMR (150 MHz, $CDCl_3$): δ 170.9, 170.5, 170.2, 170.1, 169.8, 168.74, 167.7, 167.6, 138.9, 138.8, 138.5, 138.4, 138.4, 138.2, 138.0, 128.5, 128.3, 128.2, 128.1, 128.0, 127.5, 127.2, 127.1, 123.1, 123.0, 122.7, 103.6, 101.7, 99.6, 99.2, 82.1, 81.7, 81.1, 80.1, 79.2, 76.2, 75.6, 75.1, 74.6, 73.4, 73.0, 72.6, 72.2, 71.7, 69.3, 68.1, 67.5, 62.8, 62.1, 53.3, 52.7, 51.0, 49.5, 37.5, 29.7,

23.2, 20.9, 20.7; ESI-TOF HR-MS *m/z*: calcd. for $C_{104}H_{113}N_5O_{29}$ $[M+Na]^+$ 1918.7419; found 1918.7372.

Compound 1. To a stirred solution of **17** (50 mg, 0.026 mmol) in MeOH (10 mL) was added LiOH (25 mg in 10 mL of H_2O) in portions. After being refluxed for 2 h, hydrazine monohydrate (2.5 mL) was added, and the mixture was heated at reflux for 2 d. The mixture was concentrated under vacuum, and the residue was dissolved in pyridine (5 ml). To the stirred solution was added acetic anhydride (5 ml) at rt. After stirring for 12 h, the solution was concentrated and co-evaporated with toluene 3 times to give a solid residue, which was dissolved in CH_3OH (12 mL). To the solution was added NaOMe (40 mg), and the reaction was stirred at rt for 12 h and then neutralized with 0.1 N HCl at 0 °C. To the solution was added 10% Pd-C (50.0 mg). The mixture was shaken under an H_2 atmosphere at 50 psi for 24 h. The catalyst was removed by filtration through a Celite pad, and the pad was washed with MeOH:H_2O (1:1). The combined filtrate was concentrated under vacuum and the residue was dissolved in 2 ml of H_2O and lyophilized to give the crude product that was finally purified on a Sephadex G-25 column with water as the eluent, followed by lyophilization to afford **1** (14.3 mg, 62%, over four steps) as a white solid. ^{1}H NMR (600 MHz, D_2O): δ 4.49 – 4.33 (m, 2H), 4.27 (d, *J* = 7.8 Hz, 1H), 3.98 (m, 1H), 3.94 – 3.62 (m, 12H), 3.62 – 3.34 (m, 12H), 3.26 (m, 2H), 3.13 (m, 2H), 2.55 (dd, *J* = 12.3, 4.2 Hz, 1H, H-3_{eq}), 1.97 – 1.85 (m, 6H), 1.58 (t, *J* = 12.2 Hz, 1H, H-3_{ax}); ^{13}C NMR (150 MHz, D_2O): δ 174.9, 174.9, 173.5, 103.2, 102.8, 101.8, 100.4, 79.6, 77.1, 74.8, 74.6, 74.5, 73.5, 72.6, 72.4, 72.3, 71.6, 71.1, 70.8, 68.4, 68.0, 65.7, 64.9, 62.6, 61.2, 60.1, 52.7, 51.8, 40.2, 39.4, 22.4, 22.1; ESI-TOF HR-MS *m/z*: calcd. for $C_{33}H_{57}N_3NaO_{24}$ $[M + Na]^+$ 902.3230; found 902.3221.

Compound 20. To a stirred solution of **19** (12 mg, 5 μmol) and **1** (7 mg, 8 μmol) in anhydrous DMF (3 mL) was added *N*-methylmorpholine (6 µL, 54 μmol) at rt. After being stirred for 2 d, the solution was concentrated in vacuo. The residue was purified with a preparative TLC plate (using MeOH/CH_2Cl_2/H_2O/DMF 3:3:1:1, v/v, as the eluent) to give **20** (7.0 mg, 45%) as a white powder.^{1}H NMR (600 MHz, $CDCl_3$:CD_3OD:D_2O = 5:3:1): δ 5.20 – 5.14 (m, 1H), 5.13 – 5.01 (m, 2H), 4.62 (m, 1H), 4.40 – 4.20 (m, 5H), 4.29 (m, 3H), 4.08 (m, 2H), 4.02 (m, 4H), 3.97 – 3.20 (m, 39H), 2.77 – 2.11 (m, 14H), 1.97 (2s, 6H), 1.80 – 1.41 (m, 13H), 1.40 – 1.00 (m, 98H), 0.96 – 0.78 (m, 18H); ^{31}P NMR (400 MHz, $CDCl_3$:CD_3OD:D_2O = 5:3:1): δ -2.70; ESI-TOF HR-MS *m/z*:

calcd. for $C_{169}H_{264}N_6Na_4O_{48}P$ $[M + 4Na - H]^{3+}$ 1089.5910; found 1089.7224; *m/z*: calcd. for $C_{169}H_{264}N_6Na_3O_{48}P$ $[M + 3Na - H]^{2+}$ 1622.8916; found 1623.0938.

MPLA conjugate 2. A mixture of **20** (7.0 mg, 2.2 μmol) and 10% Pd-C (20.0 mg) in CH_2Cl_2, MeOH, and H_2O (3:3:1, 5 mL) was stirred under an H_2 atmosphere at rt for 12 h. The catalyst was removed by filtration through a Celite pad, and the Celite pad was washed with a mixture of CH_2Cl_2, MeOH, and H_2O (3:3:1). The combined filtrates were concentrated in vacuum to give **2** (5.0 mg, 85%) as a white solid. ^1H NMR (600 MHz, $CDCl_3$:CD_3OD:D_2O = 3:3:1): δ 7.36 – 7.10 (m, 30H), 5.49 – 5.44 (m, 1H), 5.20 – 5.14 (m, 1H), 5.13 – 5.01 (m, 2H), 4.82 (m, 4H), 4.56 – 4.37 (m, 7H), 4.29 (m, 3H), 4.16 (m, 2H), 4.02 (m, 2H), 3.97 – 3.78 (m, 10H), 3.79 – 3.66 (m, 6H), 3.67 – 3.47 (m, 13H), 3.47 – 3.35 (m, 4H), 3.32 (m, 6H), 2.76 – 2.71 (m, 1H), 2.58 – 2.36 (m, 6H), 2.35 – 2.20 (m, 4H), 2.13 (m, 3H), 1.97 (2s, 6H), 1.73 – 1.37 (m, 13H), 1.37 – 0.99 (m, 98H), 0.96 – 0.66 (m, 18H); ^{31}P NMR (400 MHz, $CDCl_3$:CD_3OD:D_2O = 3:3:1): δ -2.44; ESI-TOF HR-MS *m/z*: calcd. for $C_{127}H_{229}N_6NaO_{48}P$ $[M + Na]^+$ 2660.5298; found 2660.5304.

Protein conjugates 3 and 4: After a mixture of **1** (3 mg) and DSG (15 eq.) in DMF and 0.1 M PBS buffer (4:1, 1 mL) was stirred at rt for 6 h, it was concentrated under reduced pressure and the residue was washed with EtOAc five times to afford the activated ester **18** [MALDI-TOF MS (positive mode) m/z: calcd. for $C_{42}H_{66}N_4NaO_{29}$ $[M + Na]^+$ 1113.37; found 1113.37], which was directly applied to the conjugation reaction with KLH and HSA. Thus, a solution of **18** and KLH or HSA (5 mg) in 0.4 mL of 0.1 M PBS buffer was gently stirred at rt for 2 d. The mixture was applied to a Biogel A0.5 column with 0.1 M PBS buffer as the eluent. Fractions containing the protein glycoconjugate, characterized by the bicinchoninic acid assay for protein, were combined and dialyzed against distilled water for 2 d. The solution was lyophilized to afford the desirable glycoconjugates **3** and **4** as white fluffy solids.

Analysis of the carbohydrate loadings of conjugates 3 and 4: The carbohydrate loadings of **3** and **4** were examined by the Svennerholm method.[65] The solution of an accurately weighted conjugate sample (0.3-0.6 mg) in distilled water (1 mL) and the resorcinol reagent (2.0 mL) was heated in a boiling water bath for 30 min and then cooled to rt. To the mixture was added the extraction solution (1-butanol acetate and 1-butanol, 85/15, v/v, 3 mL). The mixture was shaken vigorously and then allowed to stand for 10 min. The organic layer was transferred into a 1.0-cm cuvette, and

its light absorbance at the wavelength of 580 nm was determined by an UV-Vis spectrometer, using samples obtained from free proteins KLH and HSA as blank controls, respectively. The sialic acid content of each glycoconjugate was determined based on the calibration curve created with standard sialic acid samples analyzed under the same conditions and employed to calculate the carbohydrate loading of a glycoconjugate according to the following equation:

$$\text{GM2 loading (\%)} = \frac{sialic\ acid\ content(mg) in\ the\ sample}{weight\ of\ glycocojugate\ sample\ (mg)} \times \frac{molecular\ weight\ of\ GM2}{molecular\ weight\ of\ sialic\ acid} \times 100\%$$

The carbohydrate loading of the HSA conjugate **4** was further confirmed with MS analysis.

II. Raw ELISA Data and Additional Data

Table S1. Total antibody titers of pooled antisera of conjugates **2** and **3** (6 µg of GM2/dose)

Conjugate	1			2		
	Mean	SD	N	Mean	SD	N
d21	31561	5948	3	6616	1385	3
d28	20411	2683	3	19358	2394	3
d35	39633	5336	3	62457	5701	3

Table S2. Titers of various isotypes of antibodies in the individual mouse antiserum of conjugate **2** (6 µg of GM2/dose)

Mouse	1	2	3	4	5	6	Mean
kappa	50011	43058	50579	69730	53959	39566	51150
IgG1	0	0	8	0	12559	65	2105
IgG2b	170	21550	1244	16195	20396	2136	10282
IgG2c	0	0	0	0	0	0	0
IgG3	50422	7179	72709	94358	85926	50180	60129
IgM	52190	6479	71014	89117	17128	32790	44786

Table S3. Titers of various isotypes of antibodies in the individual mouse antiserum of conjugate **3** (6 µg of GM2/dose)

Mouse	1	2	3	4	5	6	Mean
kappa	41509	55763	38234	41382	49697	120778	57894
IgG1	58350	46680	35502	58695	76481	80128	59306
IgG2b	233	28900	954	2957	7	121268	25720
IgG2c	46	33	0	0	0	31614	5282
IgG3	11	0	1070	854	2	57	332
IgM	12826	7864	14221	1113	9215	13	7542

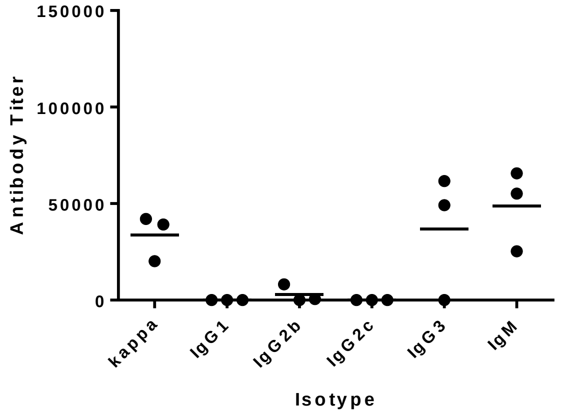

Figure S1. Titers of various isotypes of antibodies in the antiserum of individual mouse immunized with conjugate **2** containing 1 µg of GM2/injection. Each dot represents one mouse and the horizontal bar represents the average antibody titer of each group of mice.

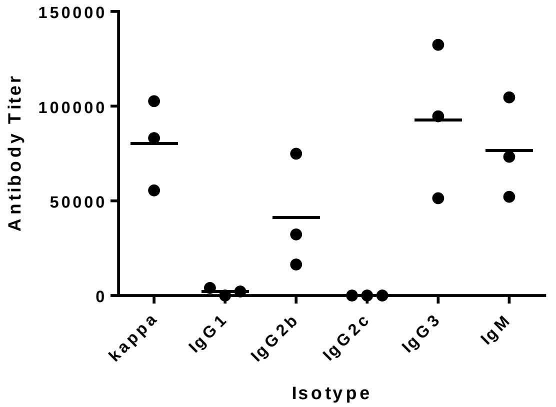

Figure S2. Titers of various isotypes of antibodies in the antiserum of individual mouse immunized with conjugate **2** containing 15 µg of GM2/injection. Each dot represents one mouse and the horizontal bar represents the average antibody titer of each group of mice.

Table S4. Titers of various isotypes of antibodies in the individual mouse antiserum of conjugate **2** (1 µg of GM2/dose)

Mouse	1	2	3	Mean
kappa	39172	20141	42112	33808
IgG1	0	1	0	0
IgG2b	48	8128	545	2907
IgG2c	0	0	0	0
IgG3	61700	3	49145	36949
IgM	25316	55125	65698	48713

Table S5. Titers of various isotypes of antibodies in the individual mouse antiserum of conjugate **2** (15 µg of GM2/dose)

Mouse	1	2	3	Mean
kappa	55596	83204	102661	80487
IgG1	5	2245	4010	2087
IgG2b	16380	74995	32310	41228
IgG2c	0	0	0	0
IgG3	51394	94627	132495	92839
IgM	73335	52226	104673	76745

III. NMR and MS Spectra of the Synthetic Intermediates and Final Products

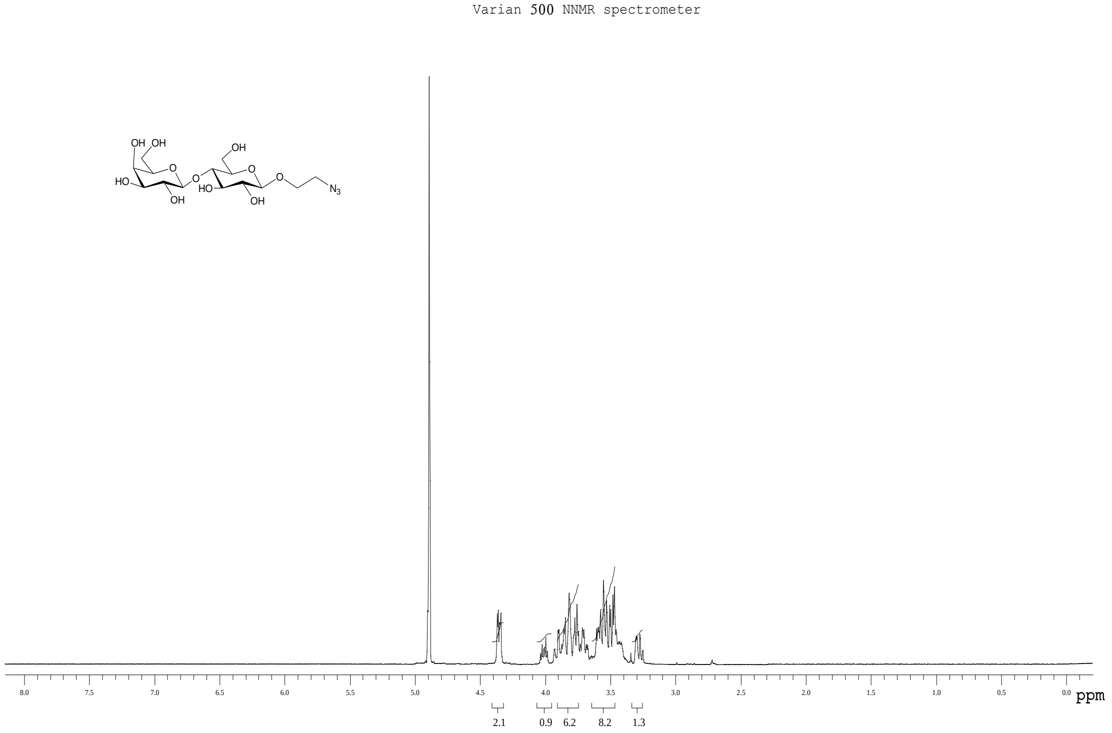

Figure S3. ^{1}H NMR spectrum of compound **8** (CD_3OD, 500 MHz)

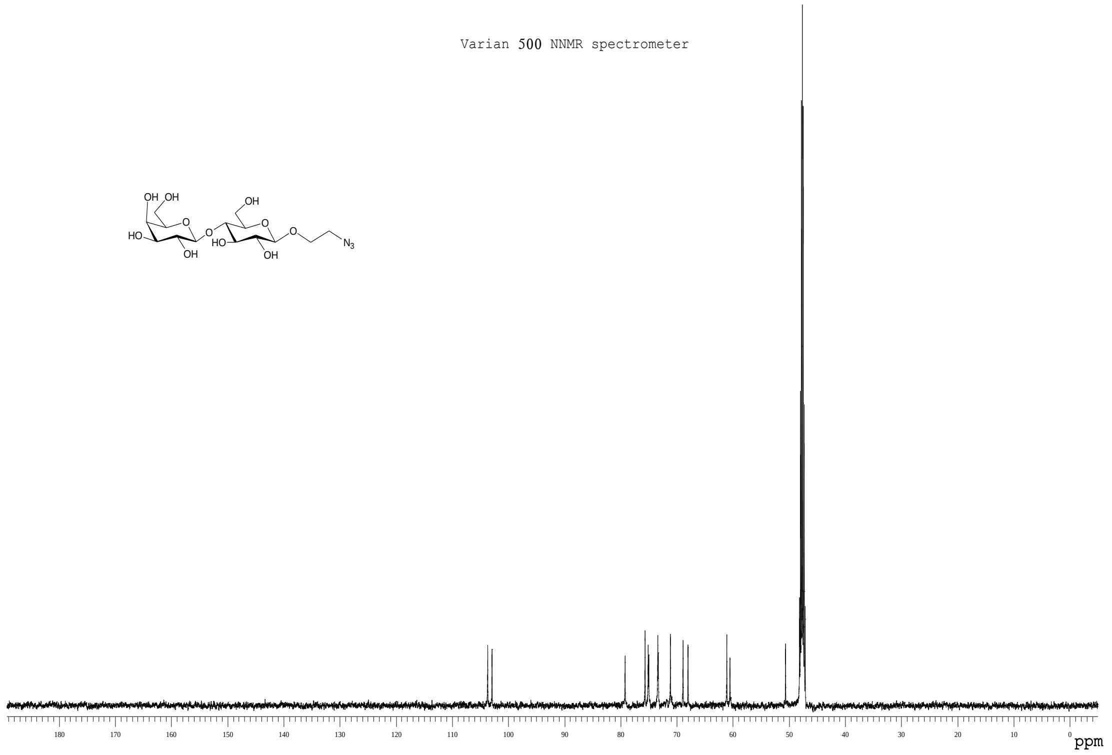

Figure S4. ^{13}C NMR spectrum of compound **8** (CD_3OD, 125 MHz)

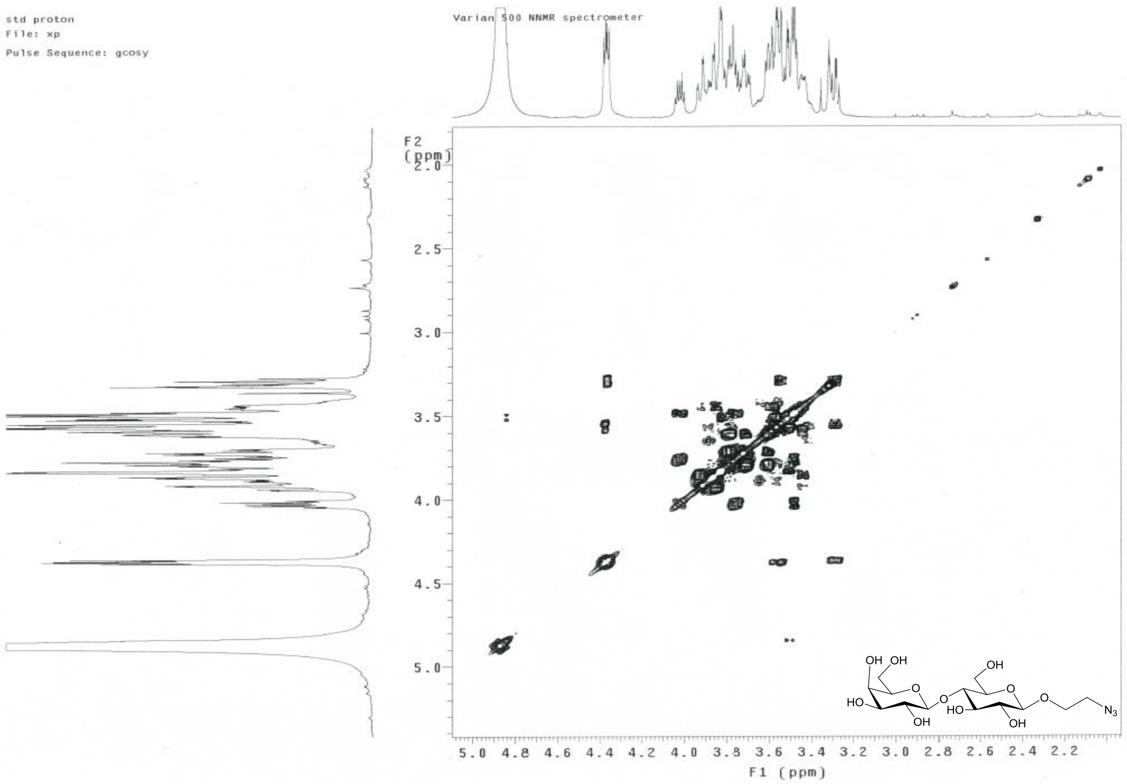

Figure S5. ^{1}H-^{1}H COSY spectrum of compound **8** (CD_3OD, 500 MHz)

INDEX	FREQUENCY	PPM	HEIGHT
1	13021.7	103.677	27.1
2	12926.3	102.918	26.1
3	9948.5	79.209	25.1
4	9502.9	75.661	34.1
5	9434.5	75.116	24.5
6	9418.2	74.987	21.8
7	9217.7	73.390	26.4
8	9206.5	73.301	25.5
9	8935.8	71.146	30.7
10	8652.5	68.890	33.8
11	8542.7	68.016	-26.1
12	7674.2	61.101	-27.8
13	7603.9	60.541	-18.6
14	6359.9	50.637	-22.6

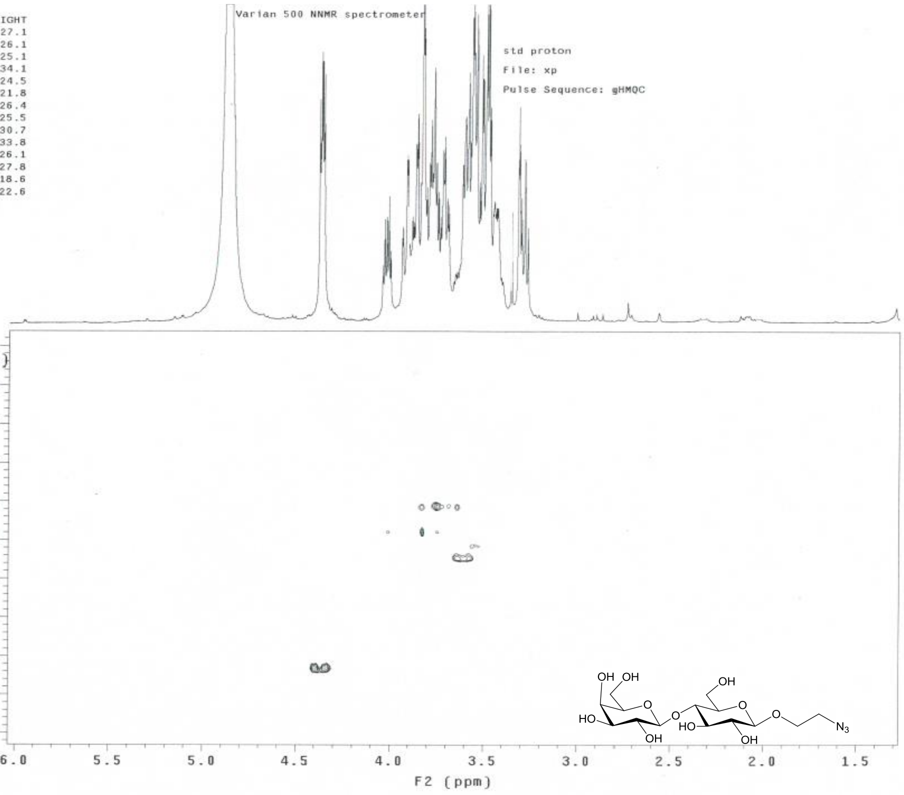

Figure S6. ^{1}H-^{13}C HMQC NMR spectrum of compound **8** (CD_3OD, 500/125 MHz)

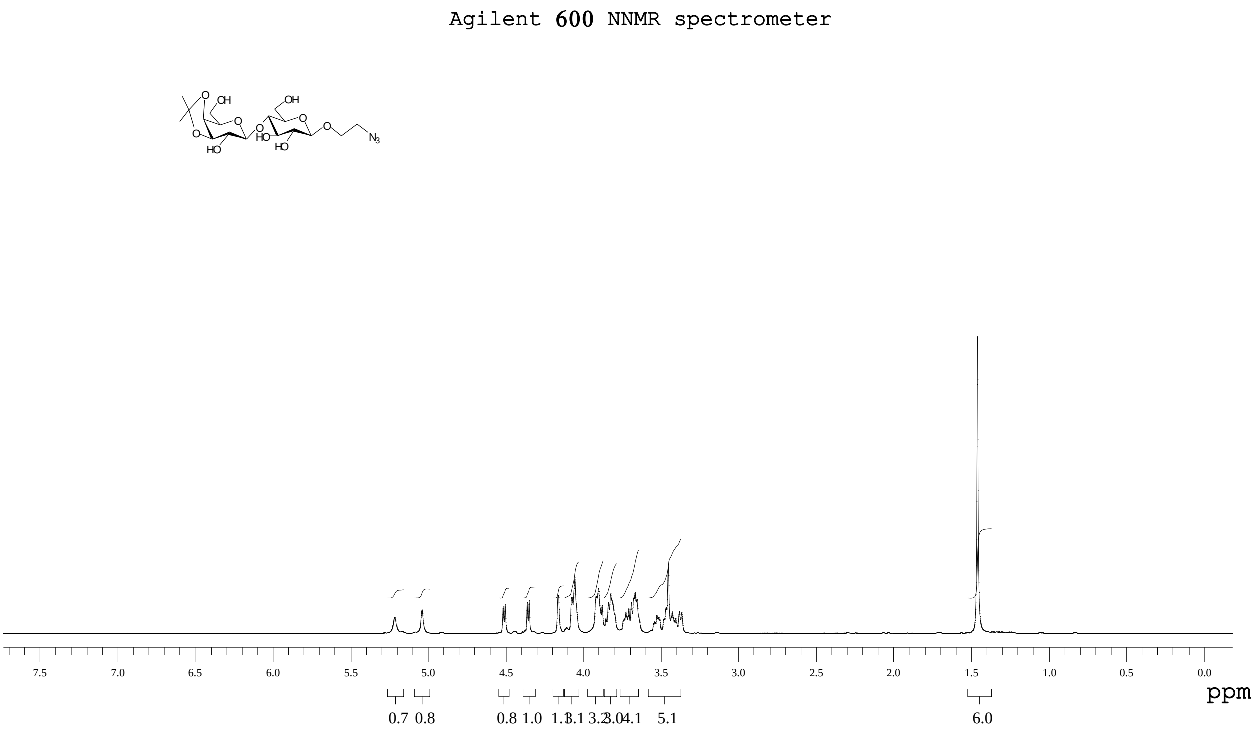

Figure S7. ^{1}H NMR spectrum of compound **9** ($CDCl_3$, 600 MHz)

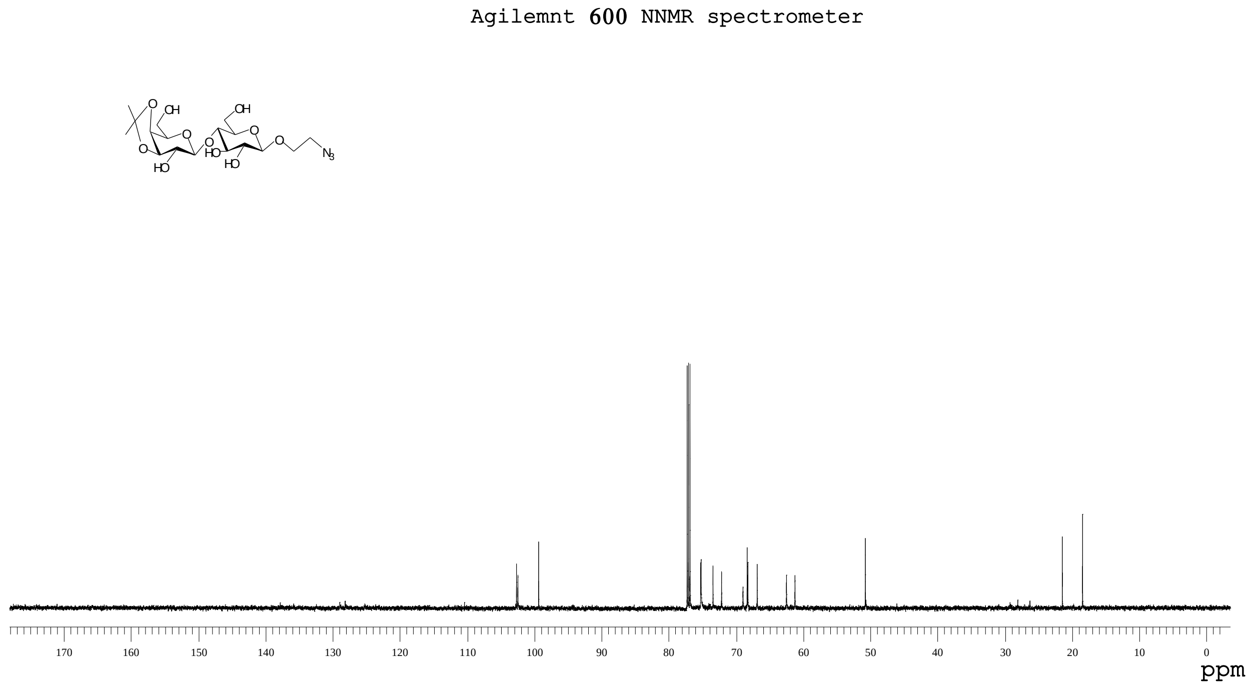

Figure S8. ^{13}C NMR spectrum of compound **9** ($CDCl_3$, 150 MHz)

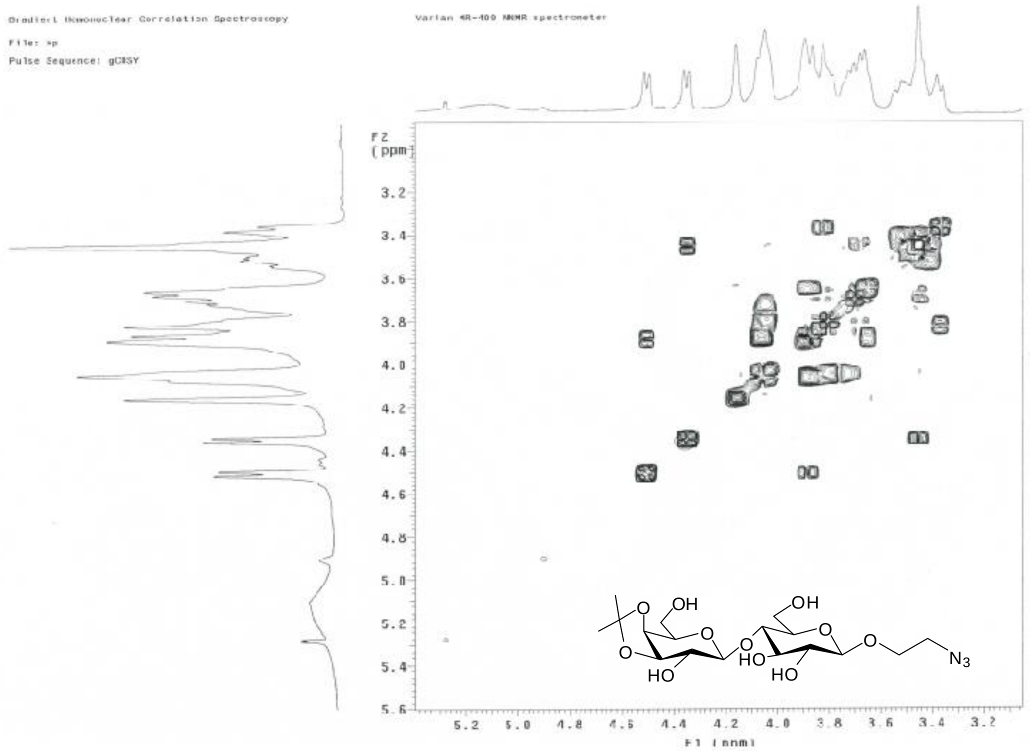

Figure S9. ^{1}H-^{1}H COSY spectrum of compound **9** ($CDCl_3$, 600 MHz)

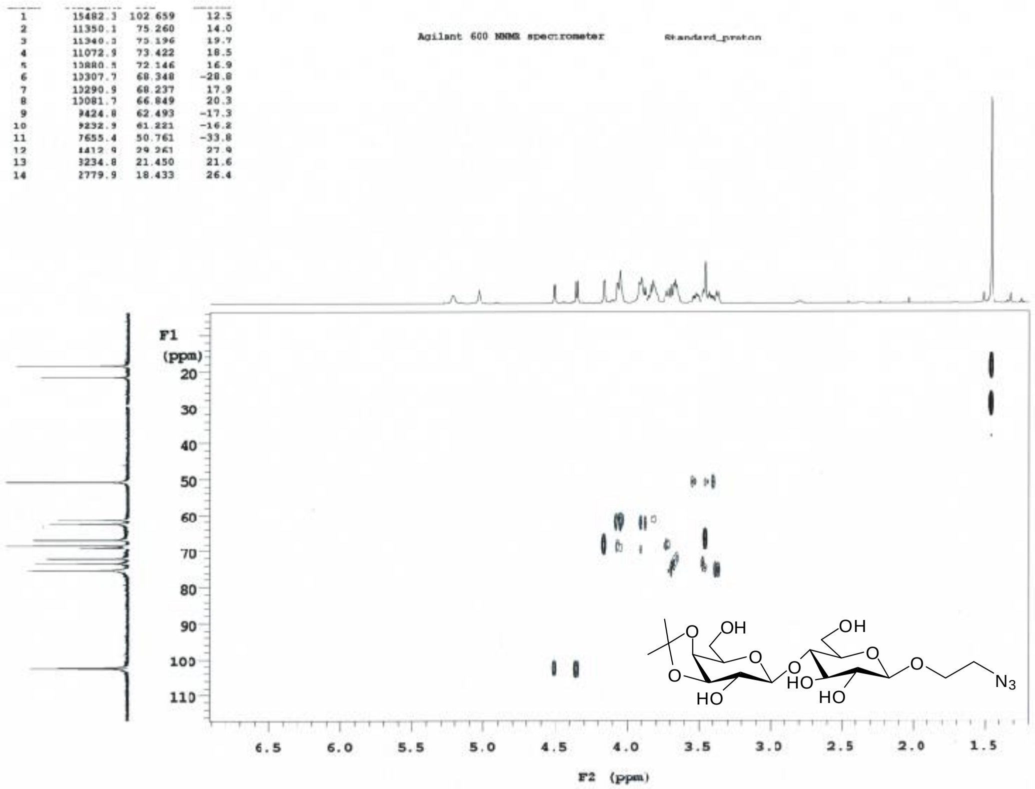

Figure S10. ^{1}H-^{13}C HMQC NMR spectrum of compound **9** ($CDCl_3$, 600/150 MHz)

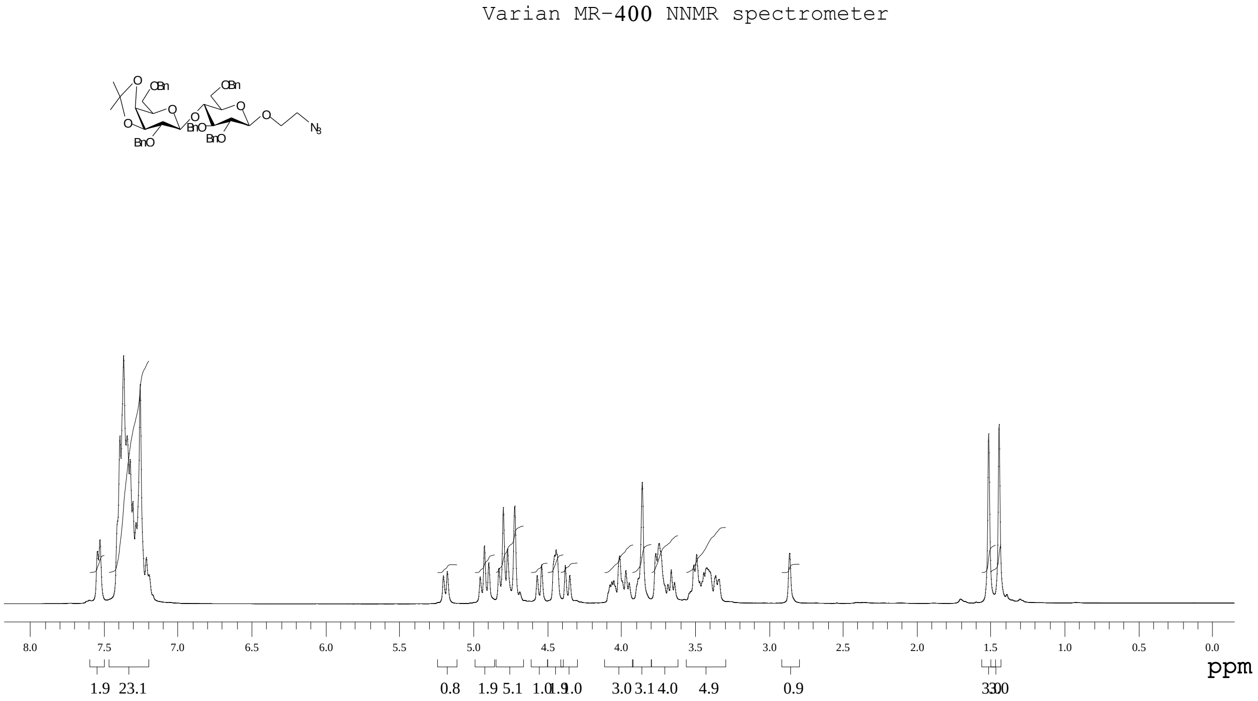

Figure S11. ^{1}H NMR spectrum of compound **10** ($CDCl_3$, 400 MHz)

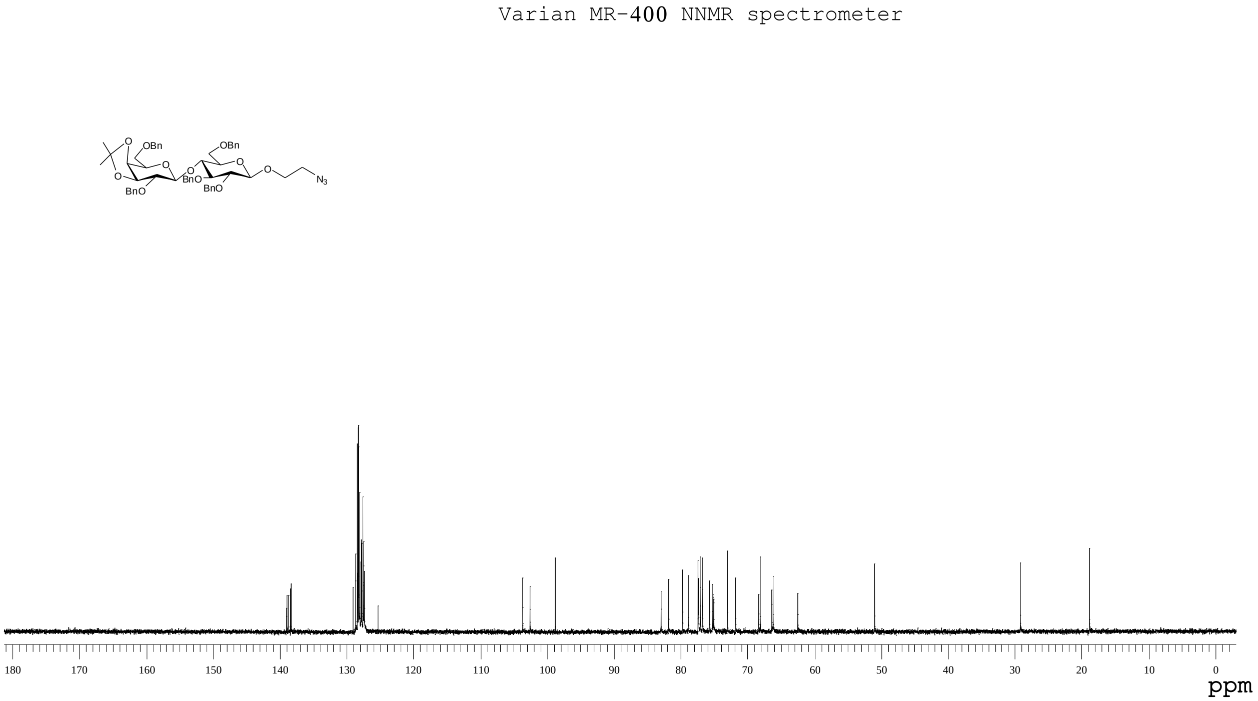

Figure S12. ^{13}C NMR spectrum of compound **10** ($CDCl_3$, 100 MHz)

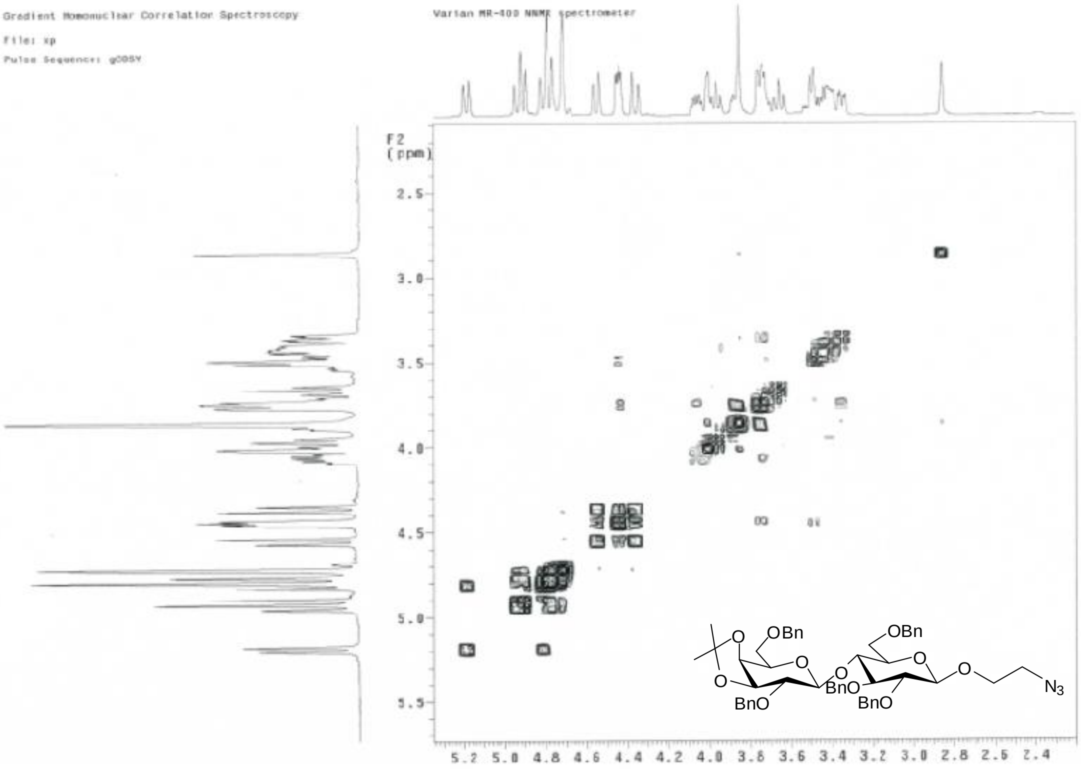

Figure S13. ^{1}H-^{1}H COSY spectrum of compound **10** ($CDCl_3$, 400 MHz)

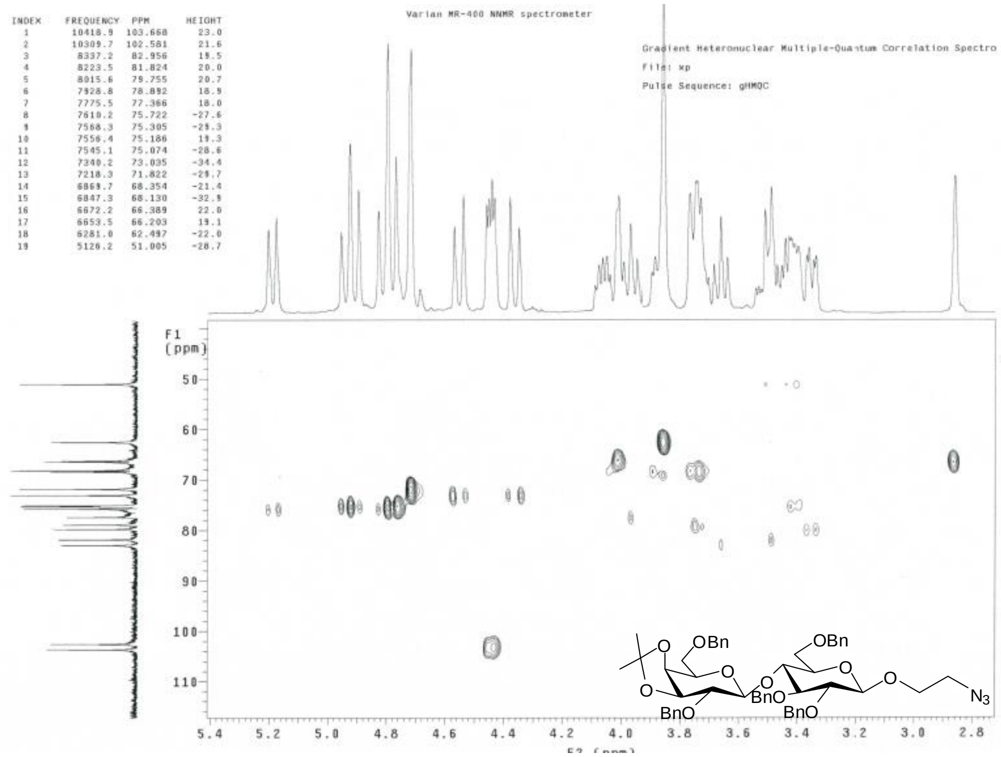

Figure S14. ^{1}H-^{13}C HMQC NMR spectrum of compound **10** ($CDCl_3$, 400/100 MHz)

Agilent 600 NNMR spectrometer

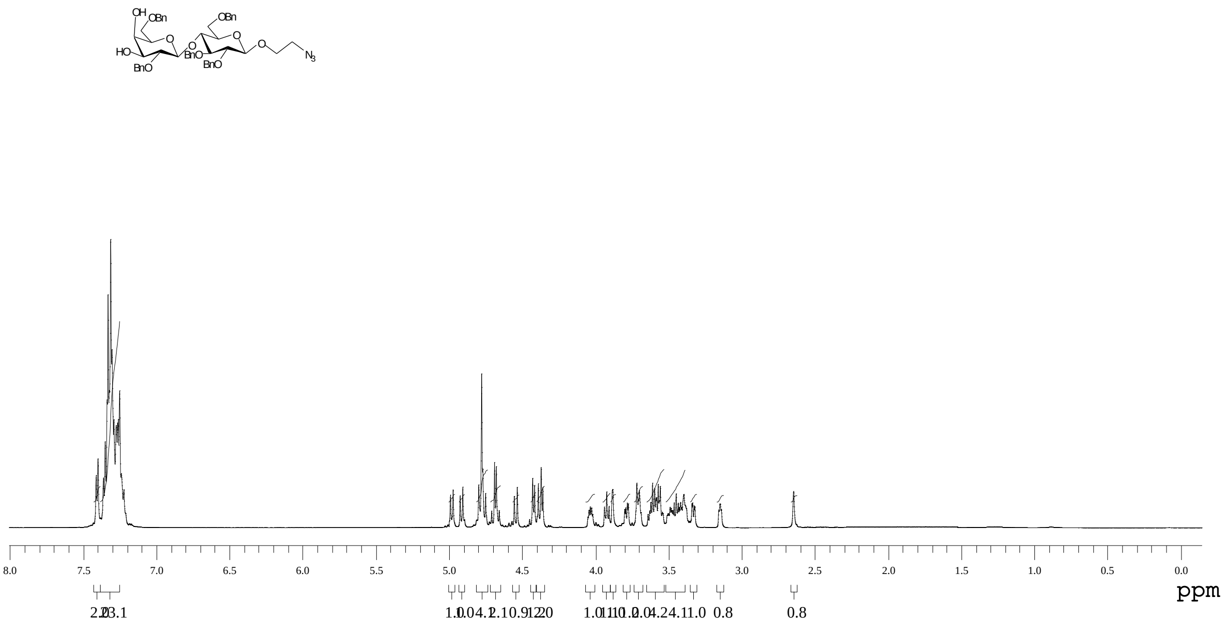

Figure S15. ^{1}H NMR spectrum of compound **5** ($CDCl_3$, 600 MHz)

Agilent 600 NNMR spectrometer

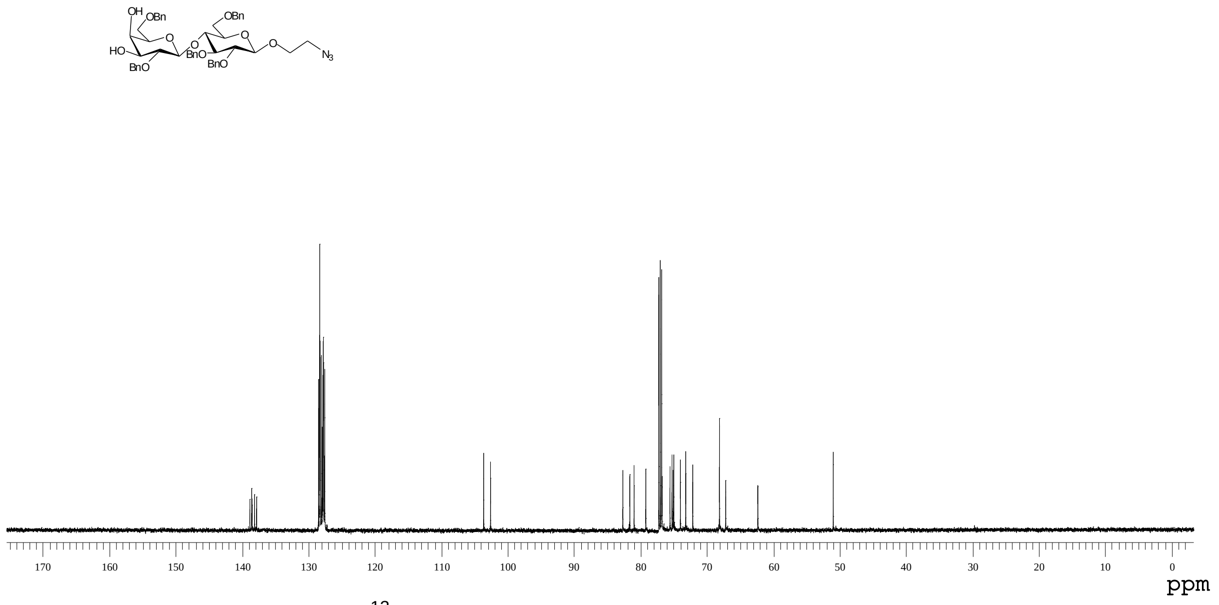

Figure S16. ^{13}C NMR spectrum of compound **5** ($CDCl_3$, 150 MHz)

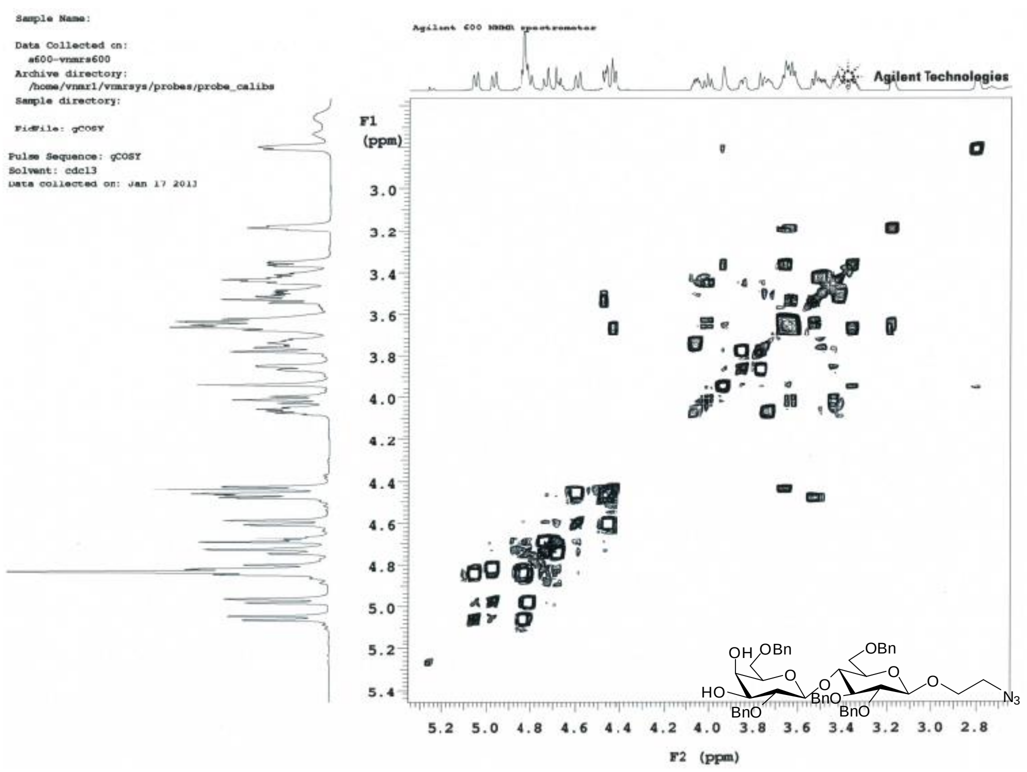

Figure S17. ^{1}H-^{1}H COSY spectrum of compound **5** ($CDCl_3$, 600 MHz)

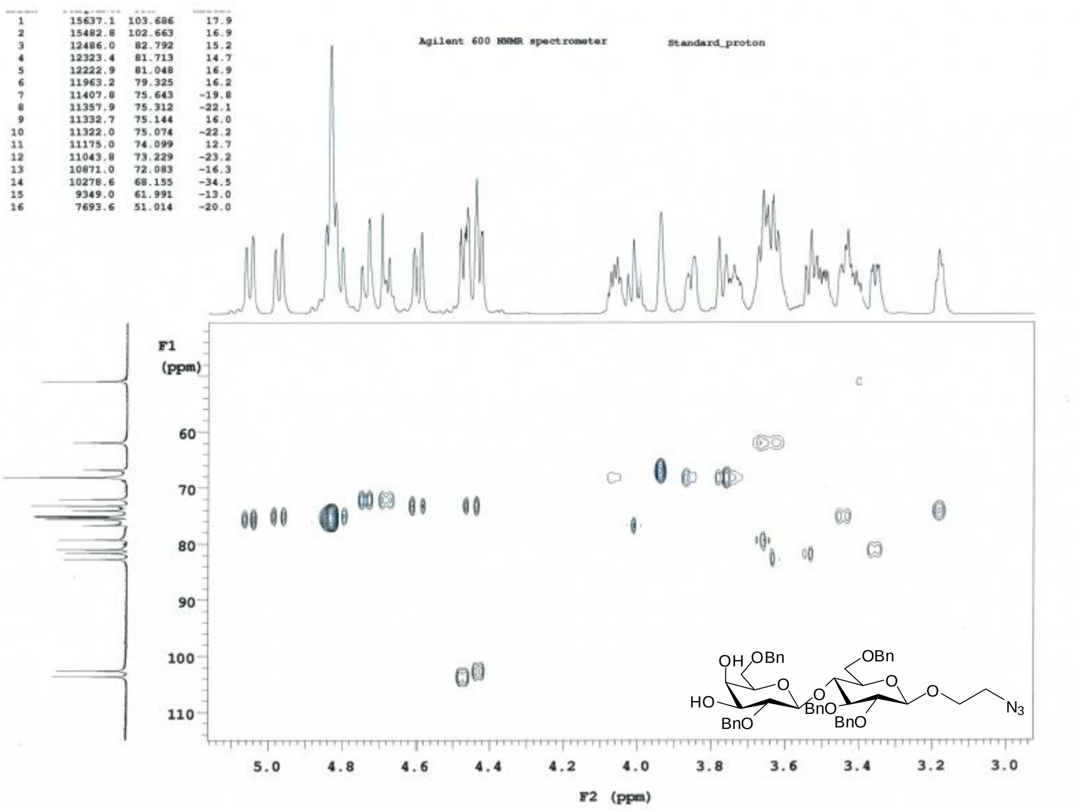

Figure S18. ^{1}H-^{13}C HMQC NMR spectrum of compound **5** ($CDCl_3$, 600/150 MHz)

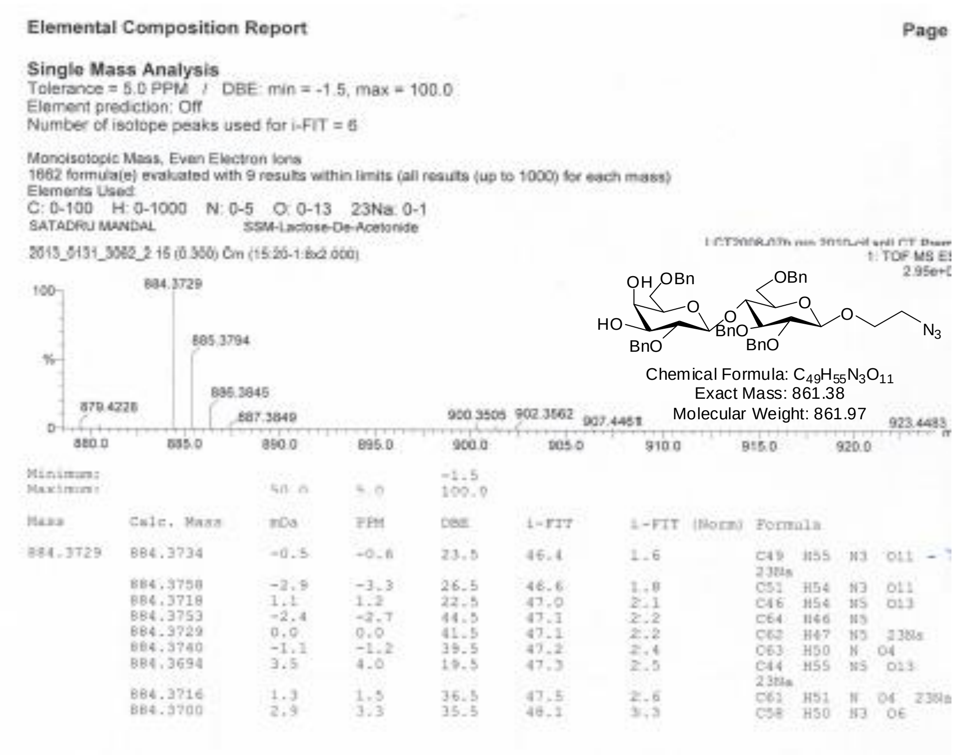

Elemental Composition Report

Page

Single Mass Analysis
Tolerance = 5.0 PPM / DBE: min = -1.5, max = 100.0
Element prediction: Off
Number of isotope peaks used for i-FIT = 6

Monoisotopic Mass, Even Electron Ions
1662 formula(e) evaluated with 9 results within limits (all results (up to 1000) for each mass)
Elements Used:
C: 0-100 H: 0-1000 N: 0-5 O: 0-13 23Na: 0-1
SATADRU MANDAL SSM-Lactose-De-Acetonide
2013_0131_3062_2 16 (0.350) Cm (15:20-1:8x2.000)

Minimum:				-1.5			
Maximum:		50.0	5.0	100.0			
Mass	Calc. Mass	mDa	PPM	DBE	i-FIT	i-FIT (Norm)	Formula
884.3729	884.3734	-0.5	-0.6	23.5	46.4	1.6	C49 H55 N3 O11 23Na
	884.3758	-2.9	-3.3	26.5	46.6	1.8	C51 H54 N3 O11
	884.3718	1.1	1.2	22.5	47.0	2.1	C46 H54 N5 O13
	884.3753	-2.4	-2.7	44.5	47.1	2.2	C64 H46 N5
	884.3729	0.0	0.0	41.5	47.1	2.2	C62 H47 N5 23Na
	884.3740	-1.1	-1.2	39.5	47.2	2.4	C63 H50 N O4
	884.3694	3.5	4.0	19.5	47.3	2.5	C44 H55 N5 O13 23Na
	884.3716	1.3	1.5	36.5	47.5	2.6	C61 H51 N O4 23Na
	884.3700	2.9	3.3	35.5	48.1	3.3	C58 H50 N3 O6

Figure S19. HR ESI-TOF MS spectrum of compound **5**

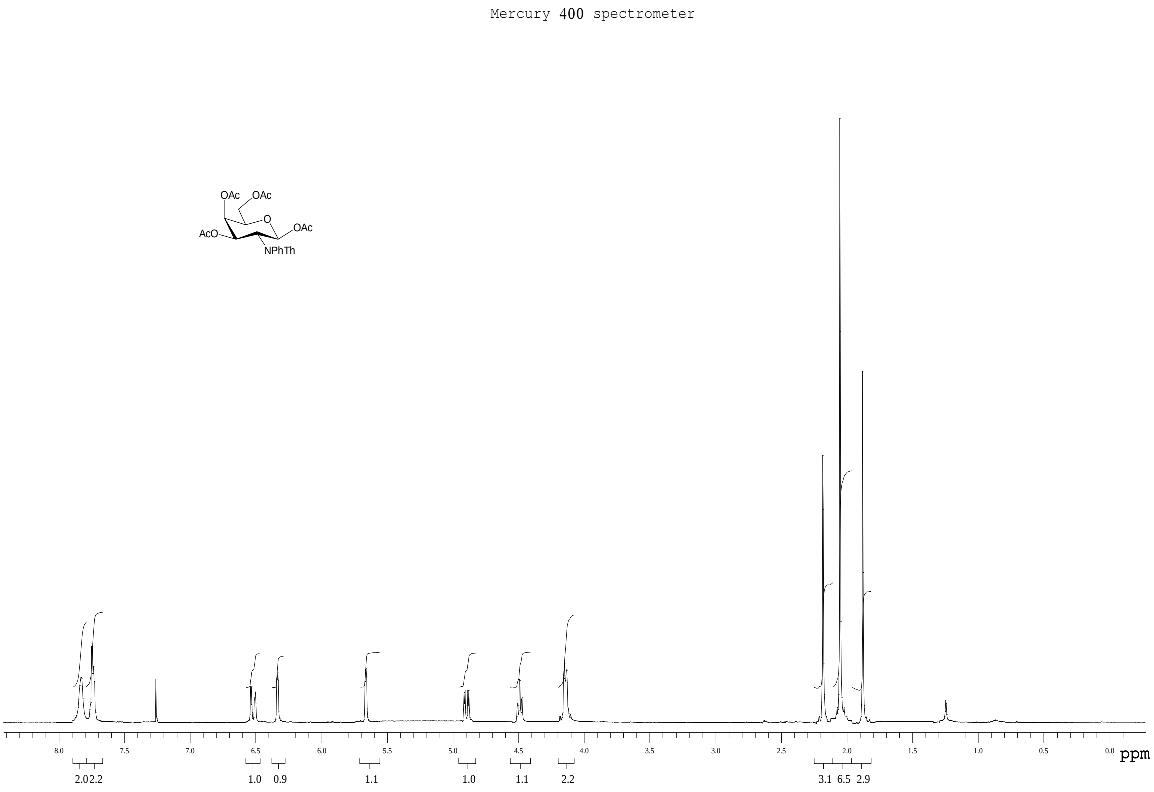

Figure S20. ^{1}H NMR spectrum of compound **12** ($CDCl_3$, 400 MHz)

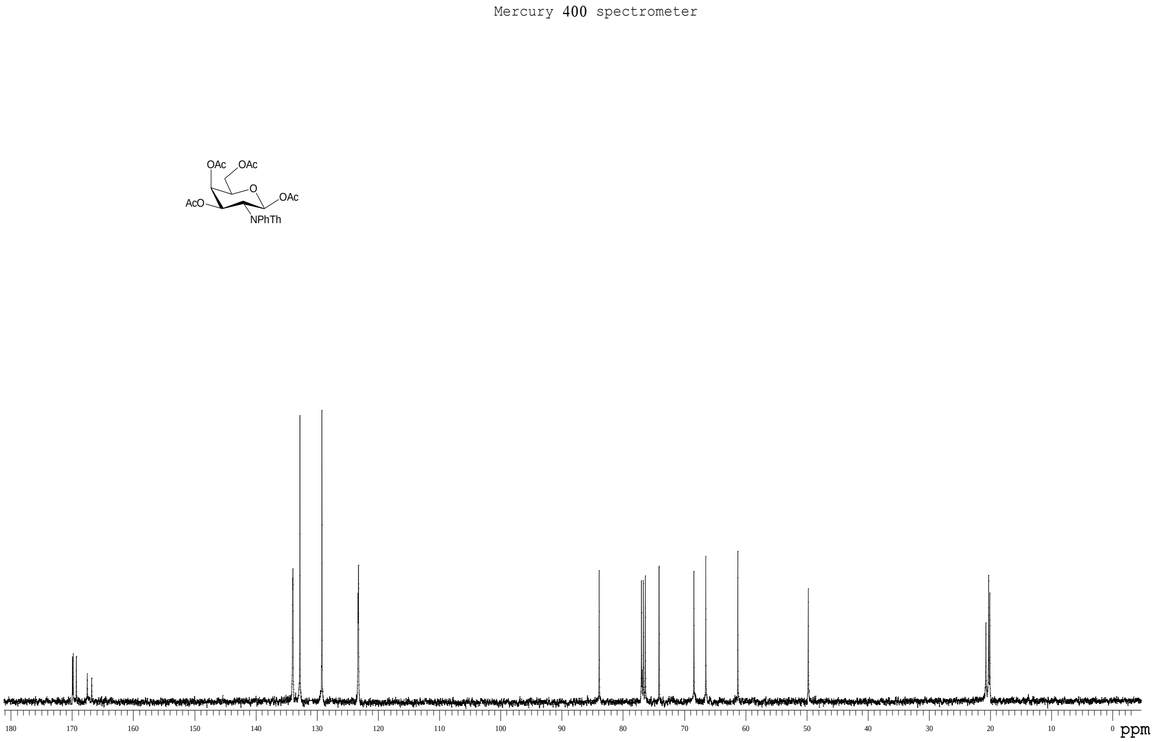

Figure S21. ^{13}C NMR spectrum of compound **12** ($CDCl_3$, 100 MHz)

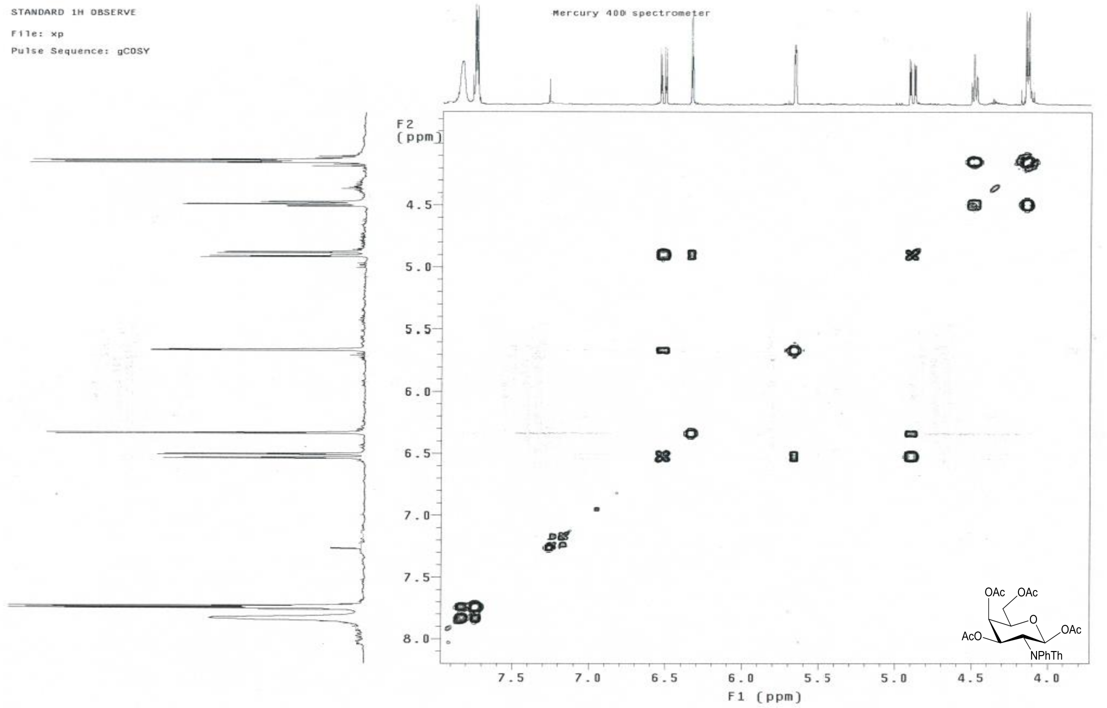

Figure S22. ^{1}H-^{1}H COSY spectrum of compound **12** ($CDCl_3$, 400 MHz)

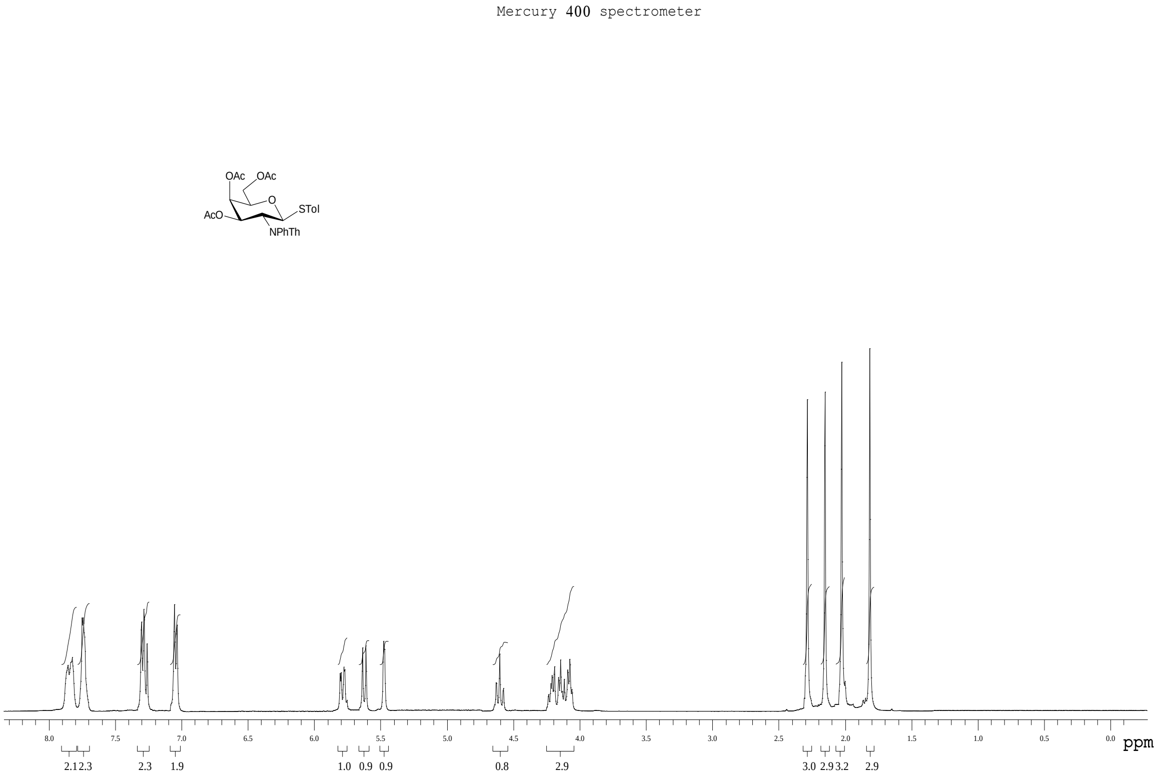

Figure S23. ^{1}H NMR spectrum of compound **13** ($CDCl_3$, 400 MHz)

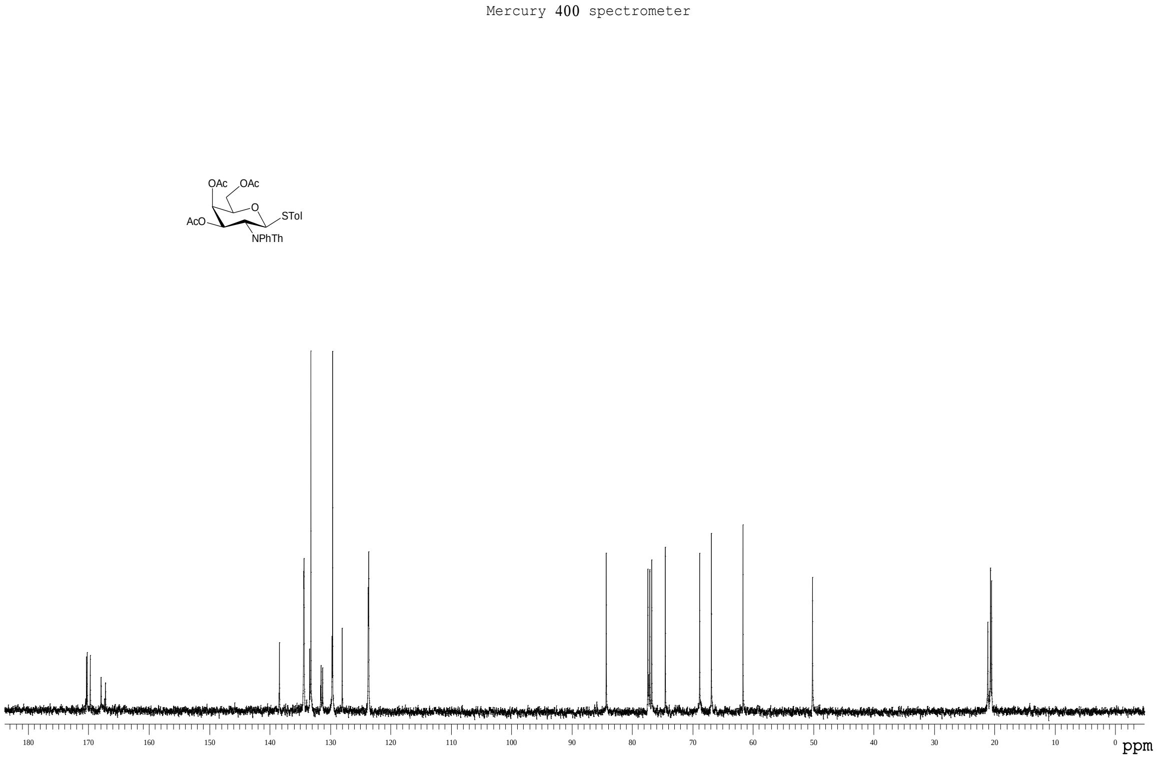

Figure S24. ^{13}C NMR spectrum of compound **13** ($CDCl_3$, 100 MHz)

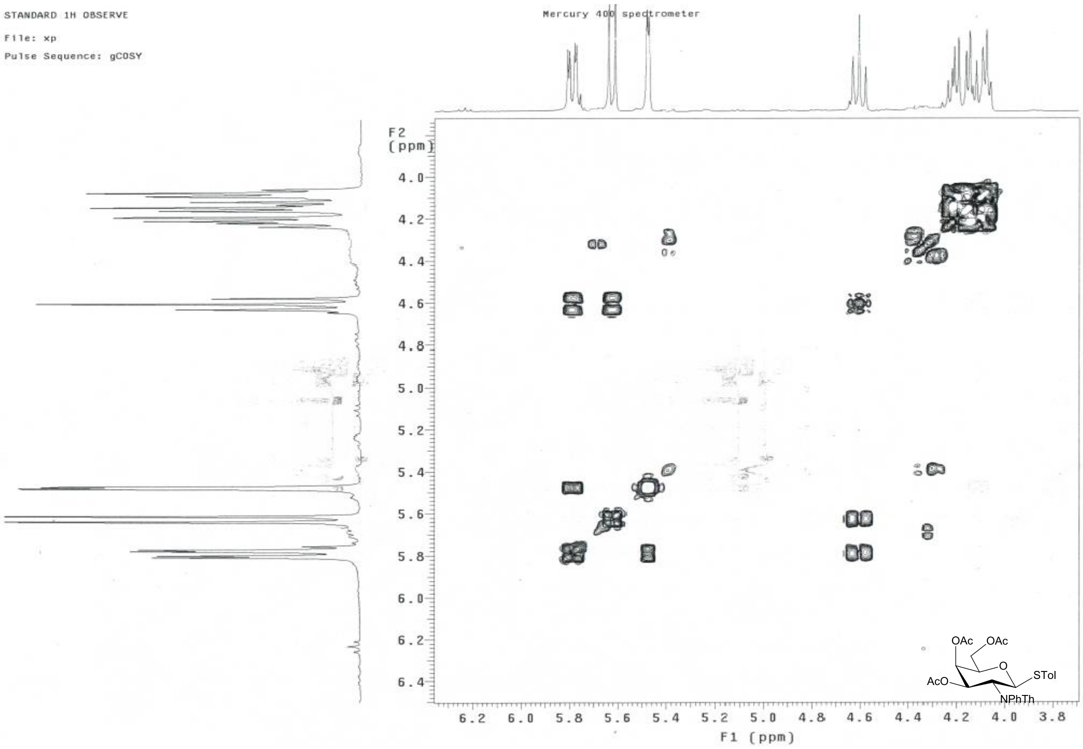

Figure S25. ^{1}H-^{1}H COSY spectrum of compound **12** ($CDCl_3$, 400 MHz)

Agilent 600 NNMR spectrometer

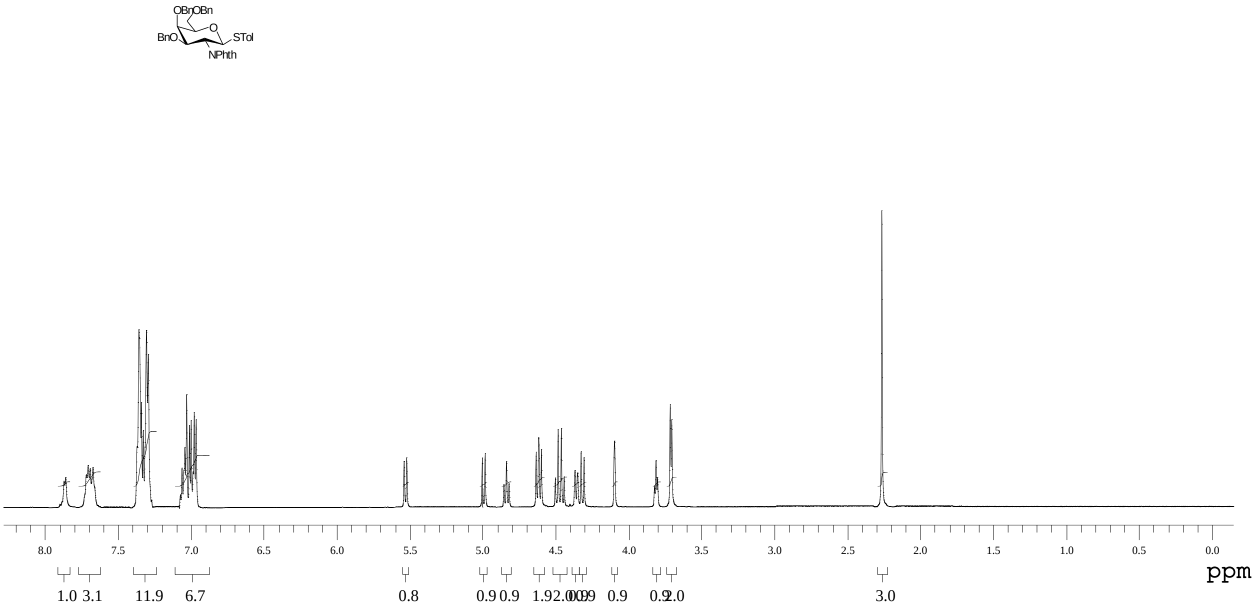

Figure S26. ^{1}H NMR spectrum of compound **6** ($CDCl_3$, 600 MHz)

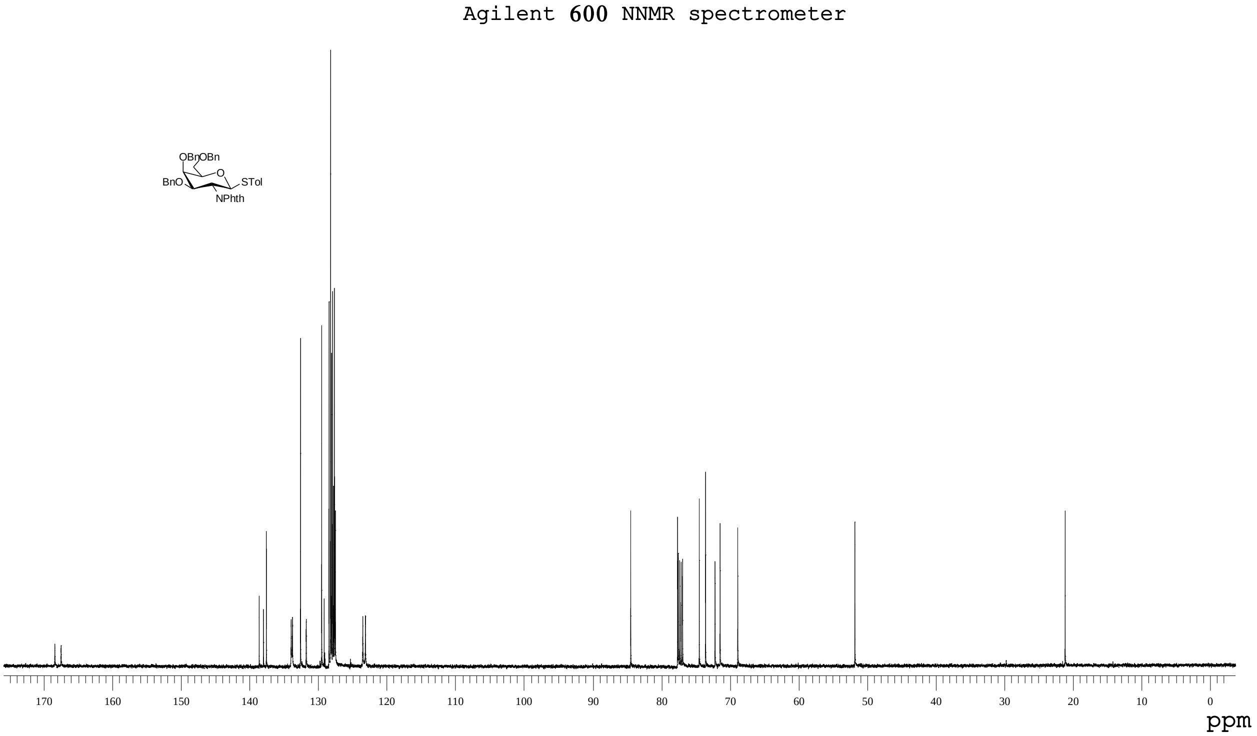

Figure S27. ^{13}C NMR spectrum of compound **6** ($CDCl_3$, 150 MHz)

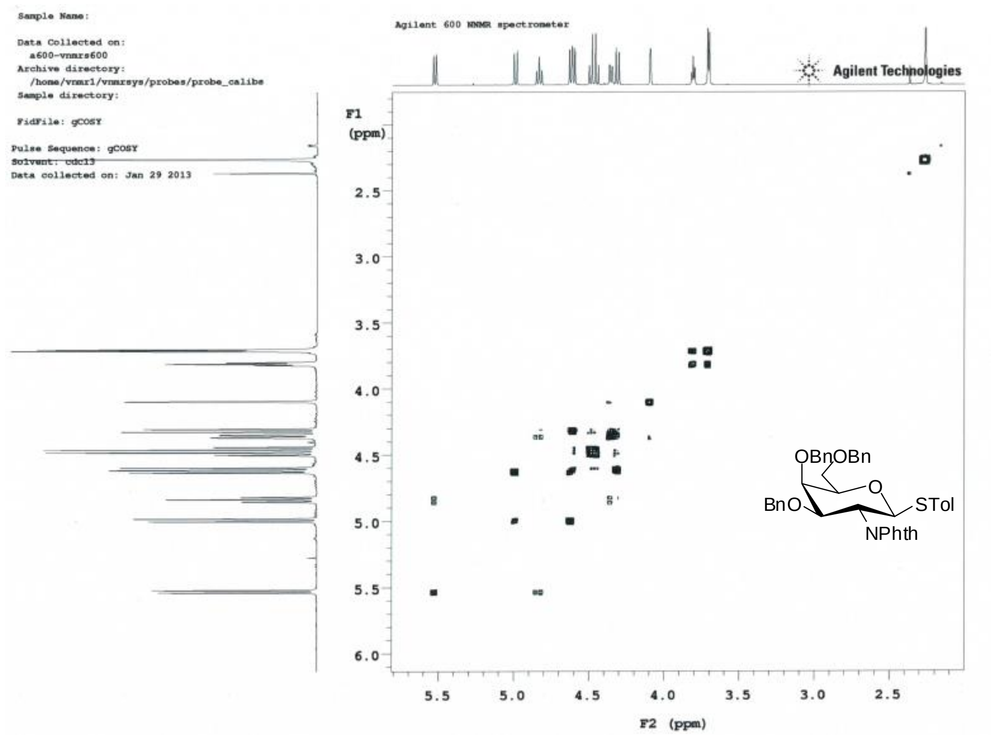

Figure S28. ^{1}H-^{1}H COSY Spectrum of compound **6** ($CDCl_3$, 600 MHz)

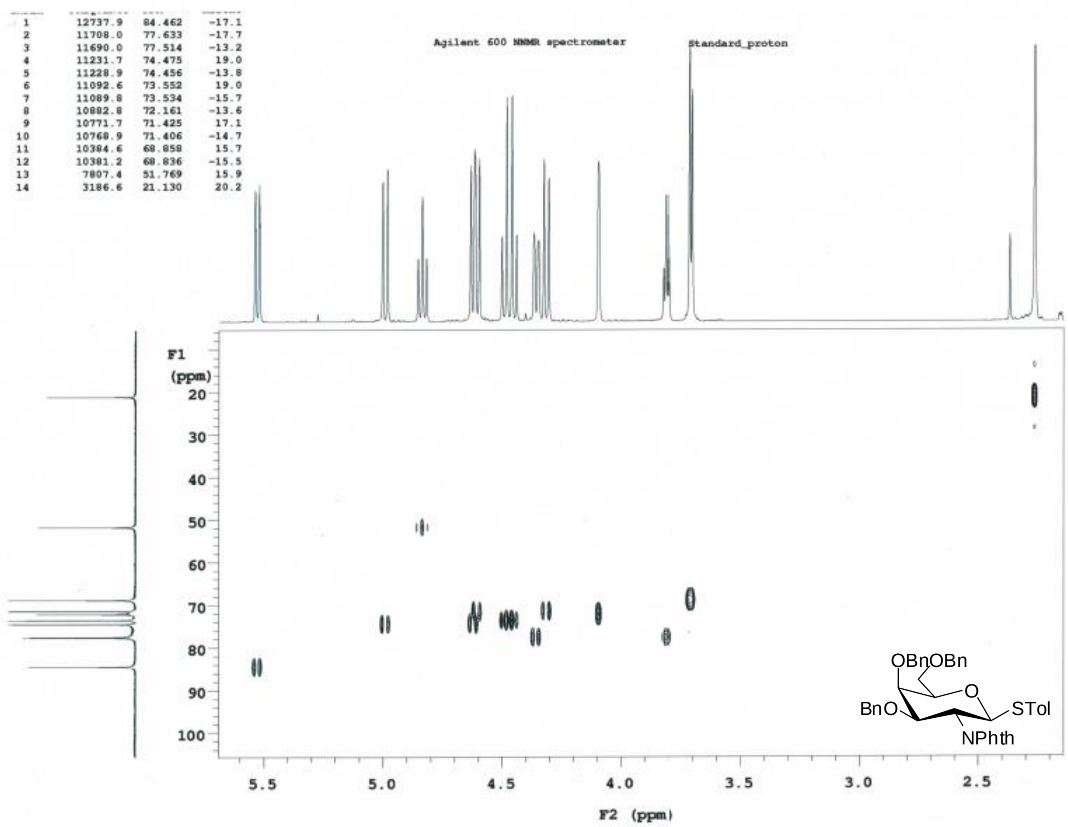

Figure S29. ^{1}H-^{13}C HMQC NMR spectrum of compound **6** ($CDCl_3$, 600/150 MHz)

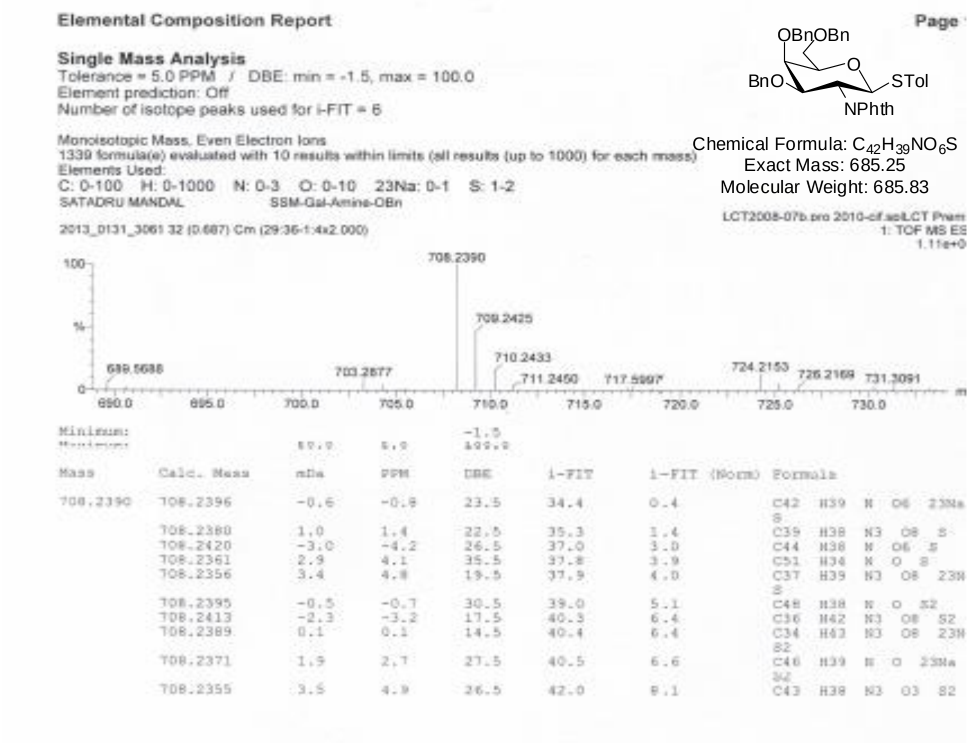

Elemental Composition Report

Single Mass Analysis
Tolerance = 5.0 PPM / DBE: min = -1.5, max = 100.0
Element prediction: Off
Number of isotope peaks used for i-FIT = 6

Monoisotopic Mass, Even Electron Ions
1339 formula(e) evaluated with 10 results within limits (all results (up to 1000) for each mass)
Elements Used:
C: 0-100 H: 0-1000 N: 0-3 O: 0-10 23Na: 0-1 S: 1-2
SATADRU MANDAL SSM-Gal-Amine-OBn
2013_0131_3061 32 (0.687) Cm (29:36-1:4x2.000)

Chemical Formula: $C_{42}H_{39}NO_6S$
Exact Mass: 685.25
Molecular Weight: 685.83

Mass	Calc. Mass	mDa	PPM	DBE	i-FIT	i-FIT (Norm)	Formula
708.2390	708.2396	-0.6	-0.8	23.5	34.4	0.4	C42 H39 N O6 23Na S
	708.2380	1.0	1.4	22.5	35.3	1.4	C39 H38 N3 O8 S
	708.2420	-3.0	-4.2	26.5	37.0	3.0	C44 H38 N O6 S
	708.2361	2.9	4.1	35.5	37.8	3.9	C51 H34 N O S
	708.2356	3.4	4.8	19.5	37.9	4.0	C37 H39 N3 O8 23N S
	708.2395	-0.5	-0.7	30.5	39.0	5.1	C48 H38 N O S2
	708.2413	-2.3	-3.2	17.5	40.3	6.4	C36 H42 N3 O8 S2
	708.2389	0.1	0.1	14.5	40.4	6.4	C34 H43 N3 O8 23N S2
	708.2371	1.9	2.7	27.5	40.5	6.6	C46 H39 N O 23Na S2
	708.2355	3.5	4.9	26.5	42.0	8.1	C43 H38 N3 O3 S2

Figure S30. HRMS ESI-TOF MS spectrum of compound **6**.

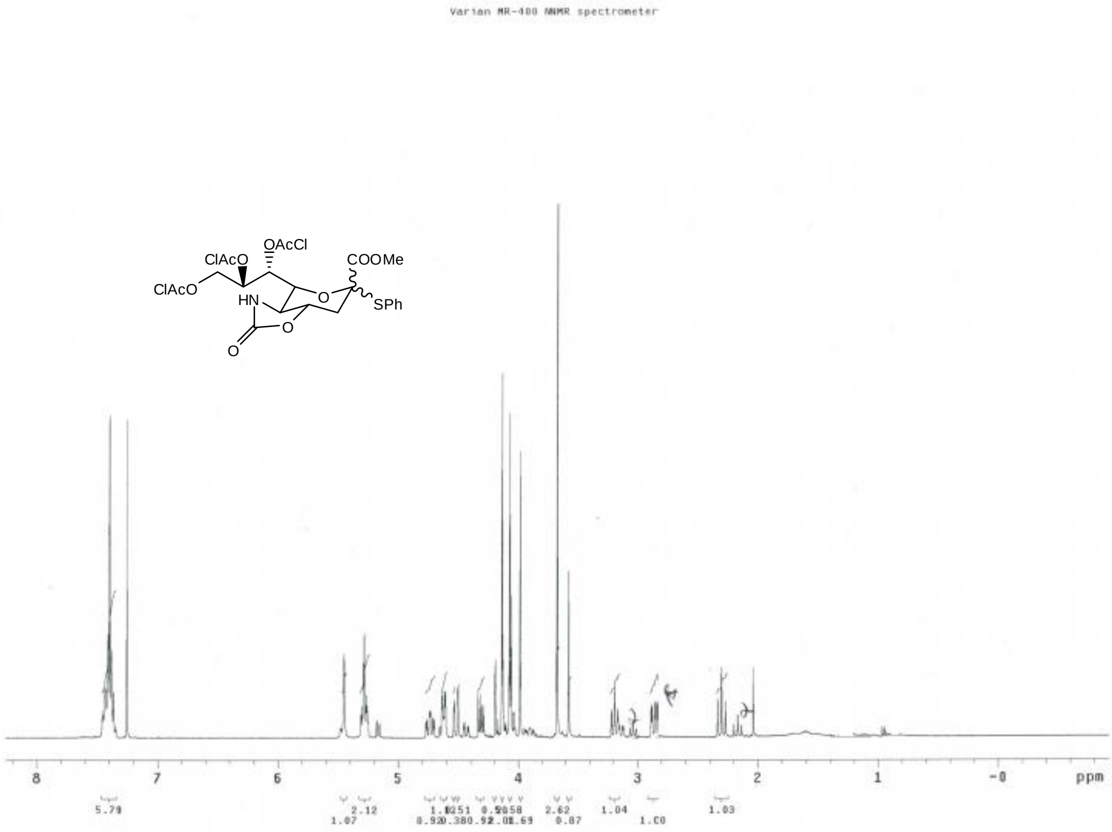

Figure S31. ^{1}H NMR spectrum of compound **7a** ($CDCl_3$, 400 MHz)

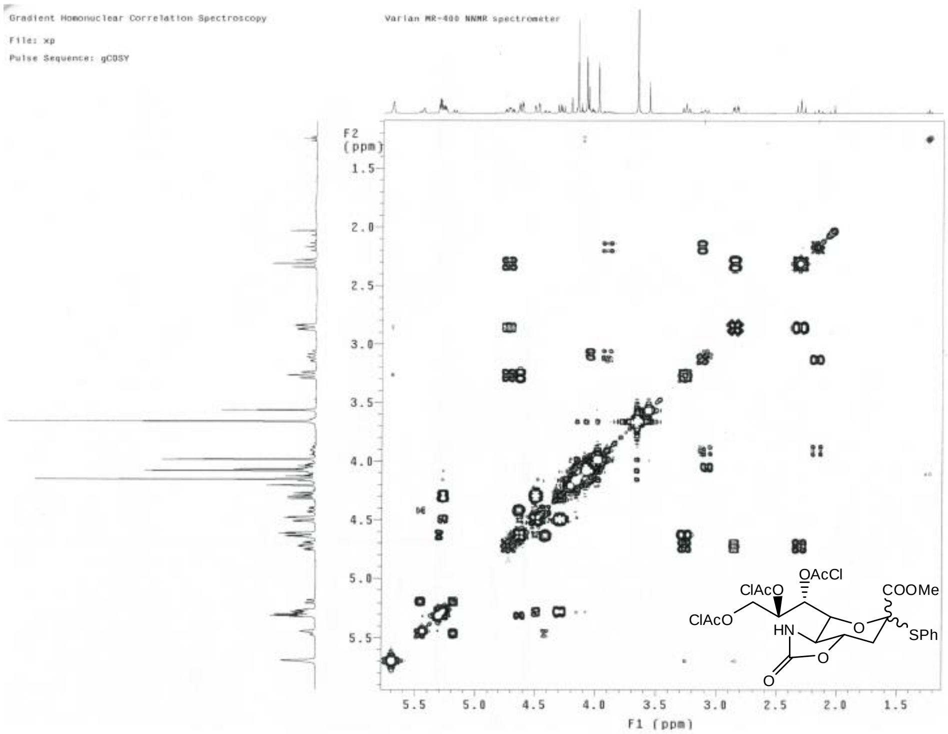

Figure S32. ^{1}H-^{1}H COSY spectrum of compound **7a** ($CDCl_3$, 400 MHz)

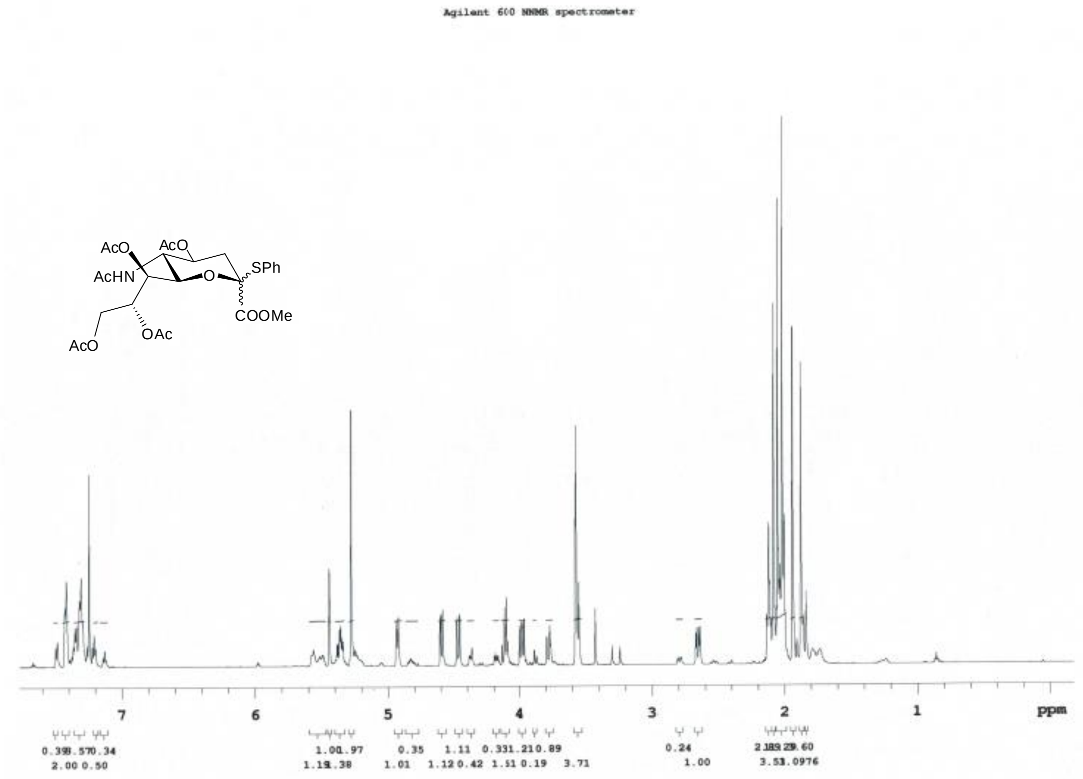

Figure S33. ^{1}H NMR spectrum of compound **7b** ($CDCl_3$, 600 MHz)

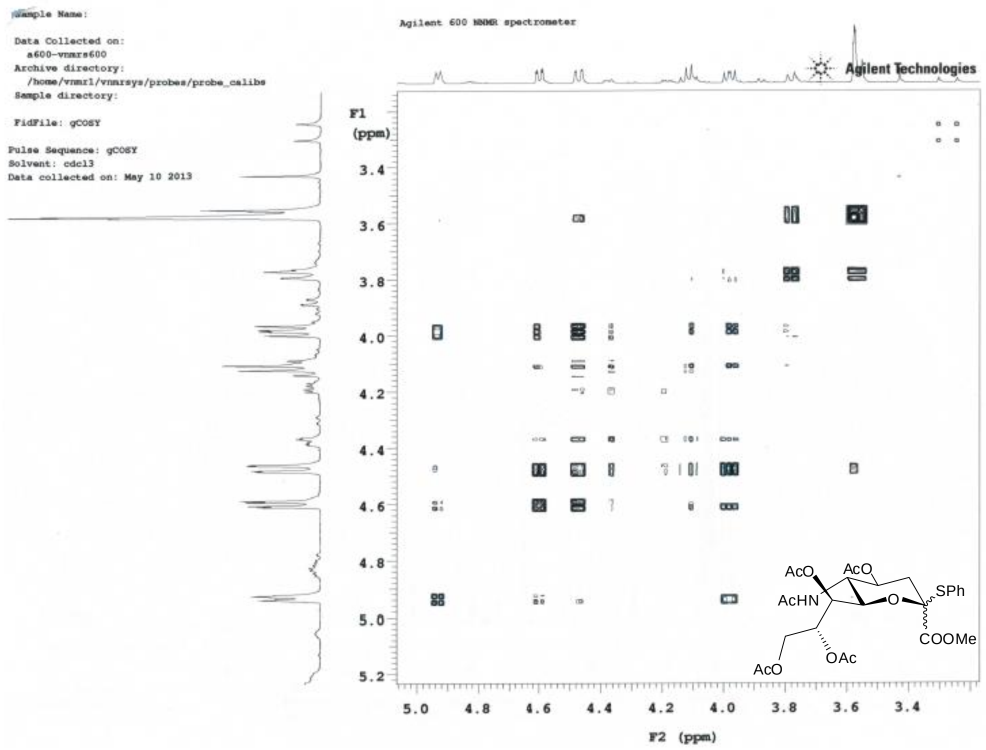

Figure S34. ^{1}H-^{1}H COSY spectrum of compound **7b** ($CDCl_3$, 600 MHz)

Agilent 600 NNMR spectrometer

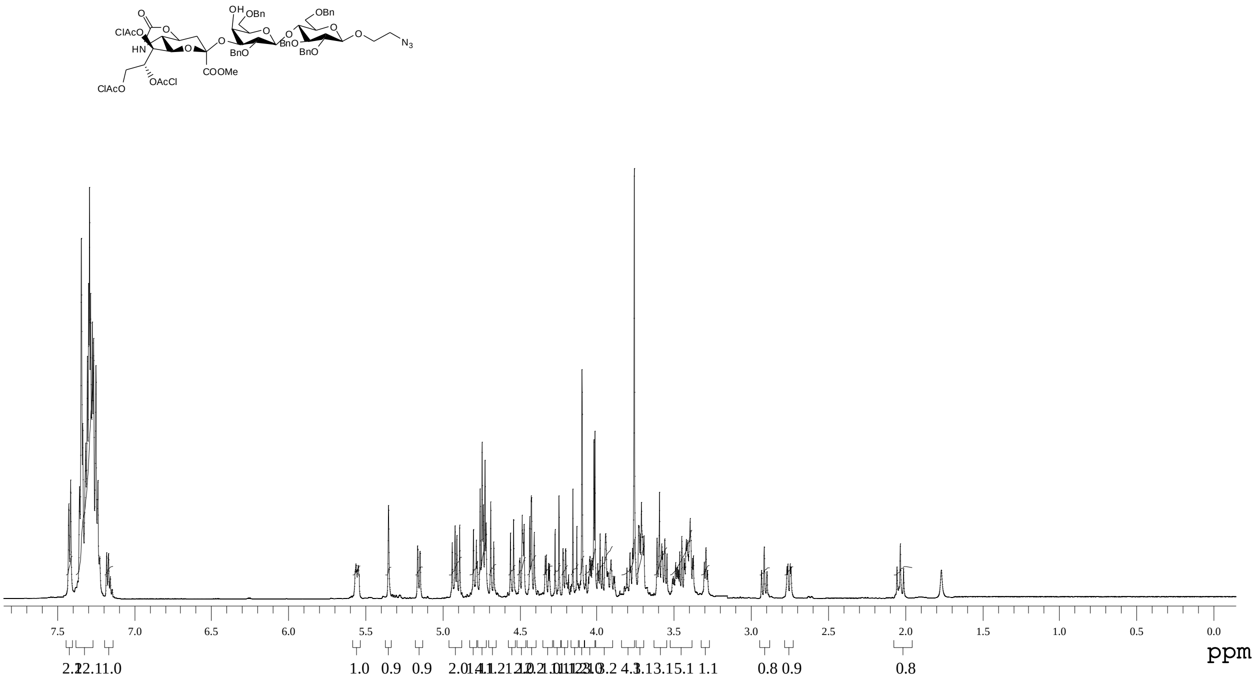

Figure S35. ^{1}H NMR spectrum of compound **14** ($CDCl_3$, 600 MHz)

Agilent 600 NNMR spectrometer

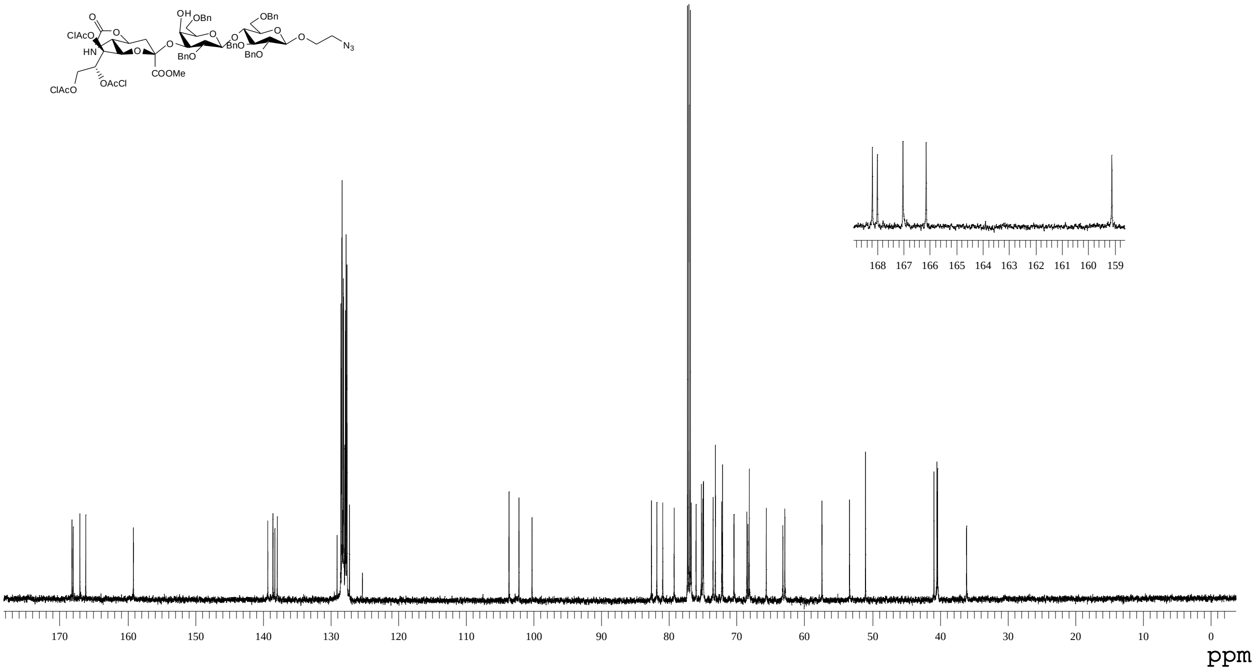

Figure S36. ^{13}C NMR spectrum of compound **14** ($CDCl_3$, 150 MHz)

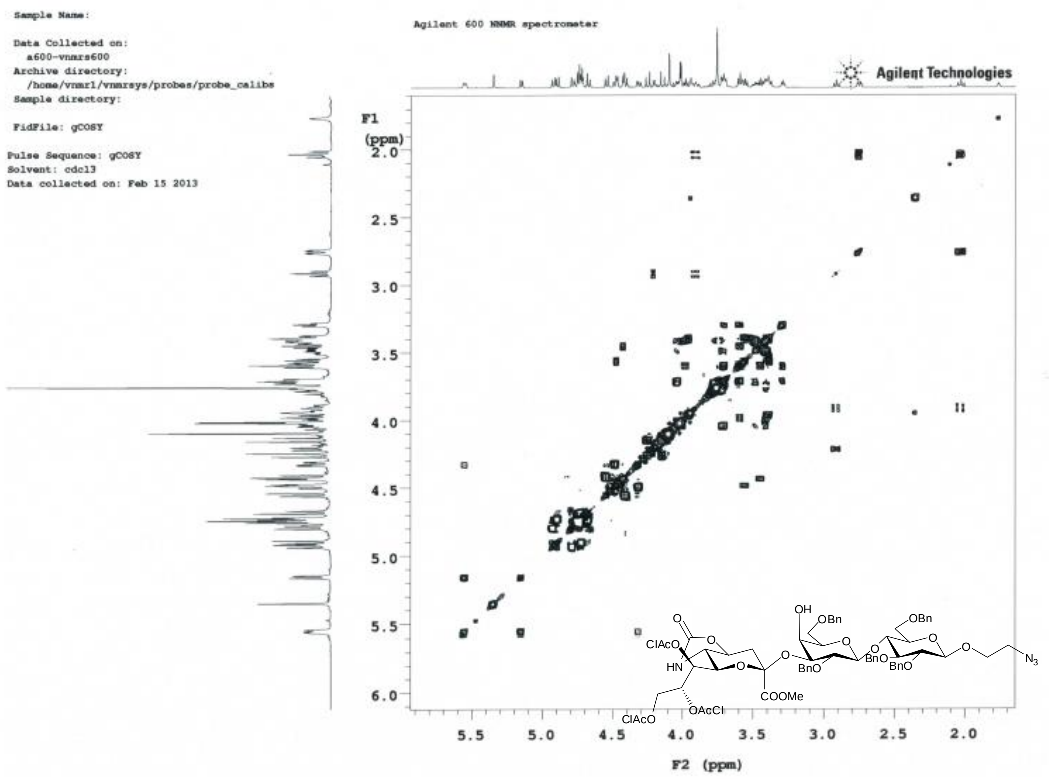

Figure S37. ^{1}H-^{1}H COSY spectrum of compound **14** ($CDCl_3$, 600 MHz)

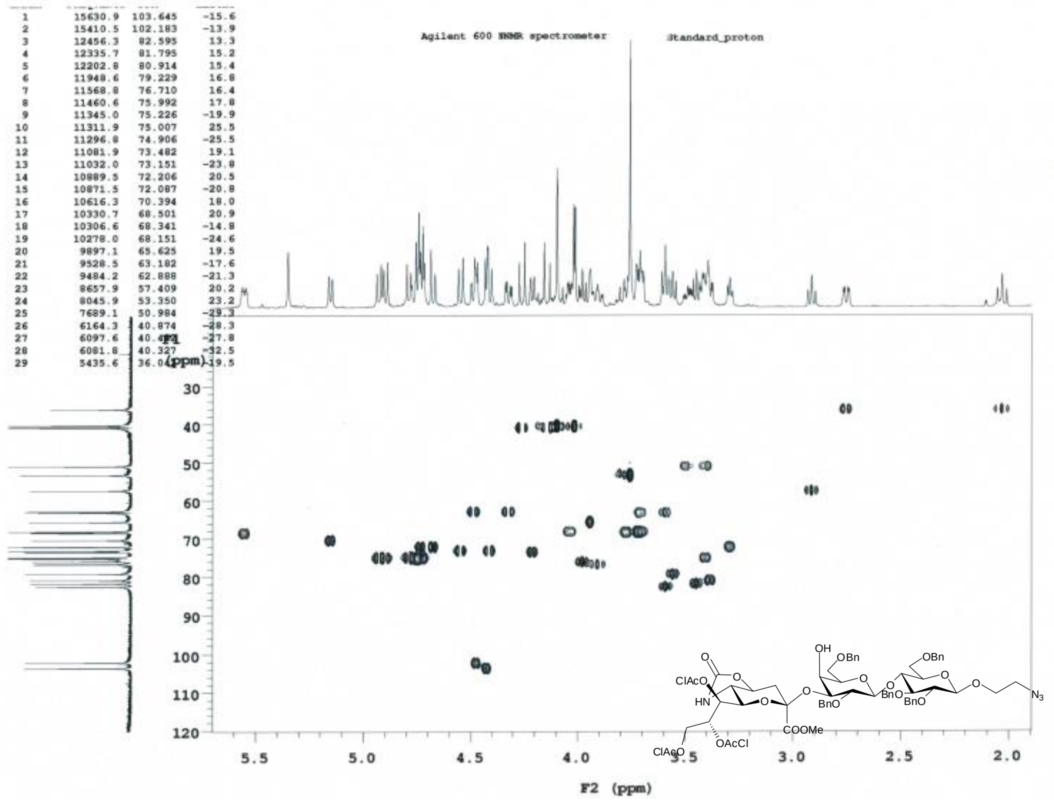

Figure S38. ^{1}H-^{13}C HMQC NMR spectrum of compound **14** ($CDCl_3$, 600/150 MHz)

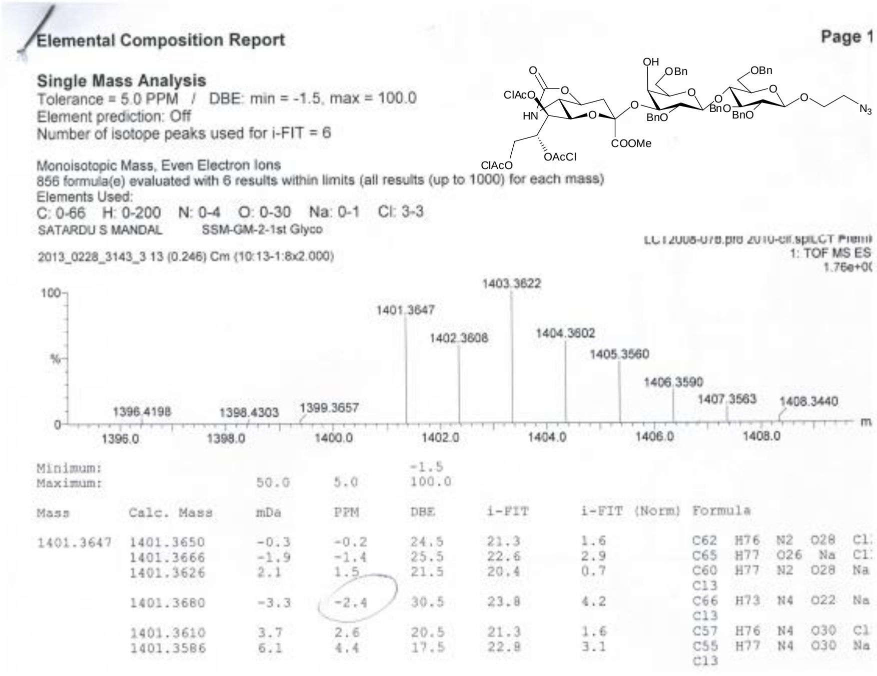

Elemental Composition Report

Page 1

Single Mass Analysis
Tolerance = 5.0 PPM / DBE: min = -1.5, max = 100.0
Element prediction: Off
Number of isotope peaks used for i-FIT = 6

Monoisotopic Mass, Even Electron Ions
856 formula(e) evaluated with 6 results within limits (all results (up to 1000) for each mass)
Elements Used:
C: 0-66 H: 0-200 N: 0-4 O: 0-30 Na: 0-1 Cl: 3-3
SATARDU S MANDAL SSM-GM-2-1st Glyco
2013_0228_3143_3 13 (0.246) Cm (10:13-1:8x2.000)

Minimum:				-1.5			
Maximum:		50.0	5.0	100.0			
Mass	Calc. Mass	mDa	PPM	DBE	i-FIT	i-FIT (Norm)	Formula
1401.3647	1401.3650	-0.3	-0.2	24.5	21.3	1.6	C62 H76 N2 O28 Cl:
	1401.3666	-1.9	-1.4	25.5	22.6	2.9	C65 H77 O26 Na Cl:
	1401.3626	2.1	1.5	21.5	20.4	0.7	C60 H77 N2 O28 Na C13
	1401.3680	-3.3	-2.4	30.5	23.8	4.2	C66 H73 N4 O22 Na C13
	1401.3610	3.7	2.6	20.5	21.3	1.6	C57 H76 N4 O30 Cl
	1401.3586	6.1	4.4	17.5	22.8	3.1	C55 H77 N4 O30 Na C13

Figure S39. HRMS ESI-TOF MS spectrum of compound **14**

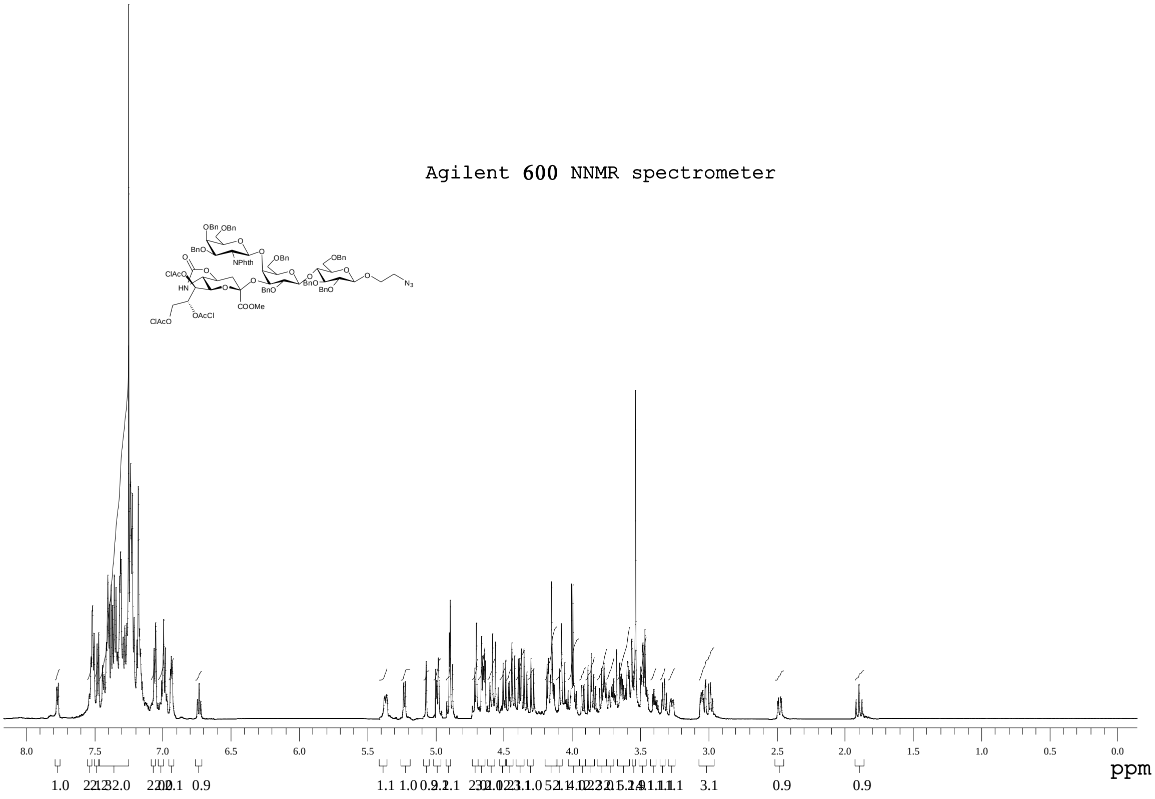

Figure S40. ^{1}H NMR spectrum of compound **15** ($CDCl_3$, 600 MHz)

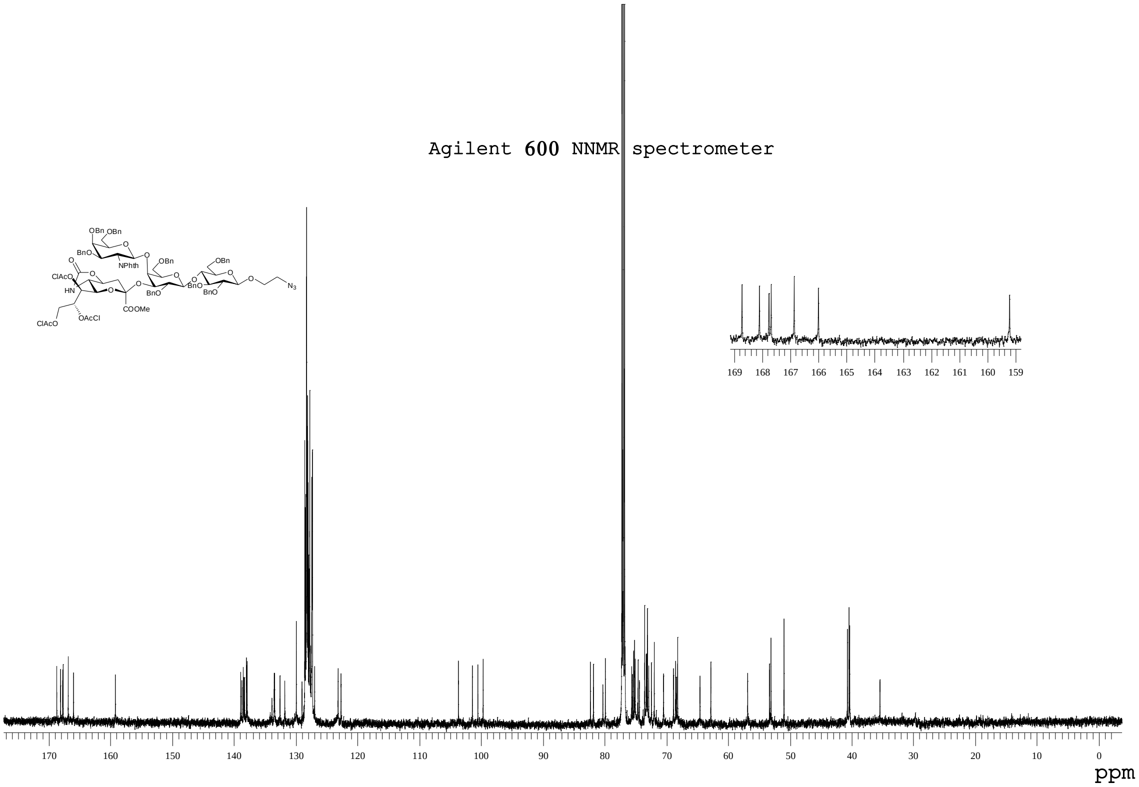

Figure S41. ^{13}C NMR spectrum of compound **15** ($CDCl_3$, 150 MHz)

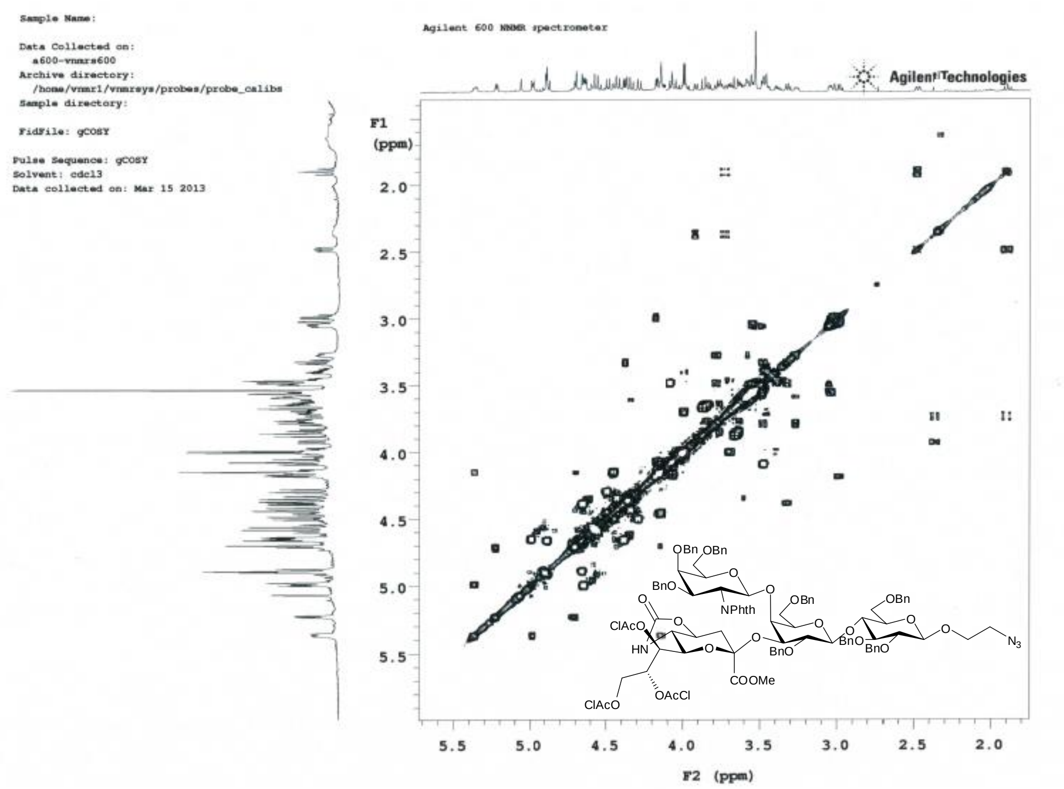

Figure S42. ^{1}H-^{1}H COSY spectrum of compound **15** ($CDCl_3$, 600 MHz)

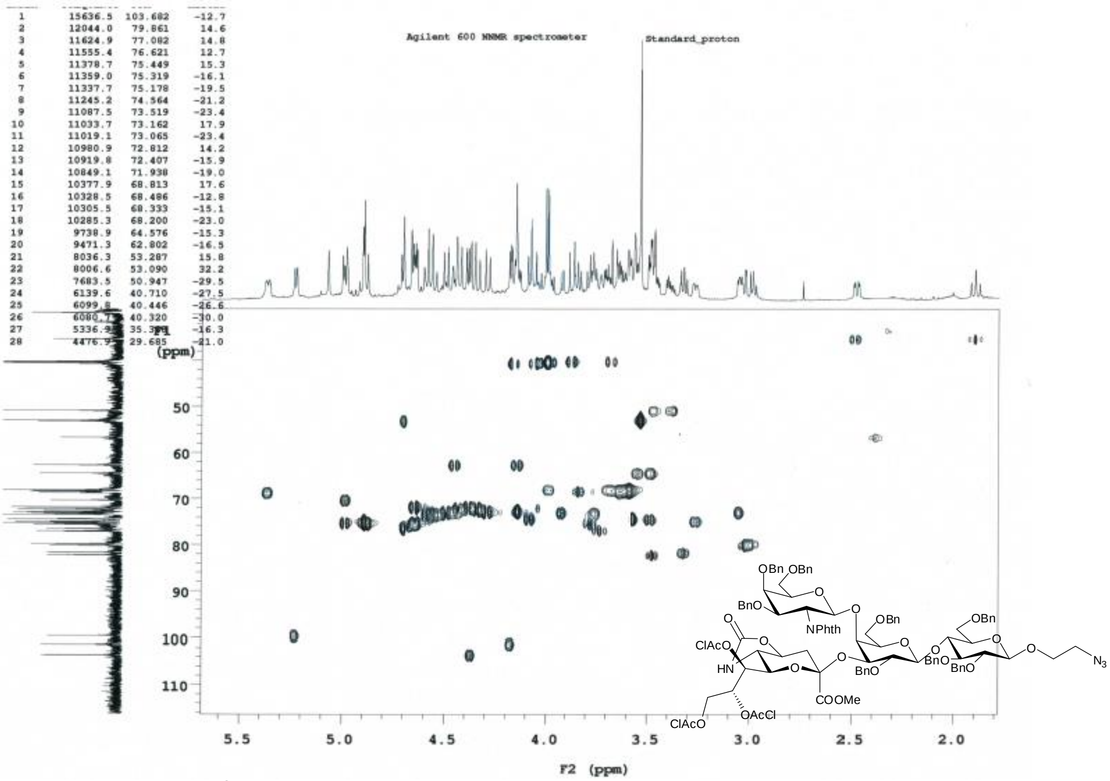

Figure S43. ^{1}H-^{13}C HMQC NMR spectrum of compound **15** ($CDCl_3$, 600/150 MHz)

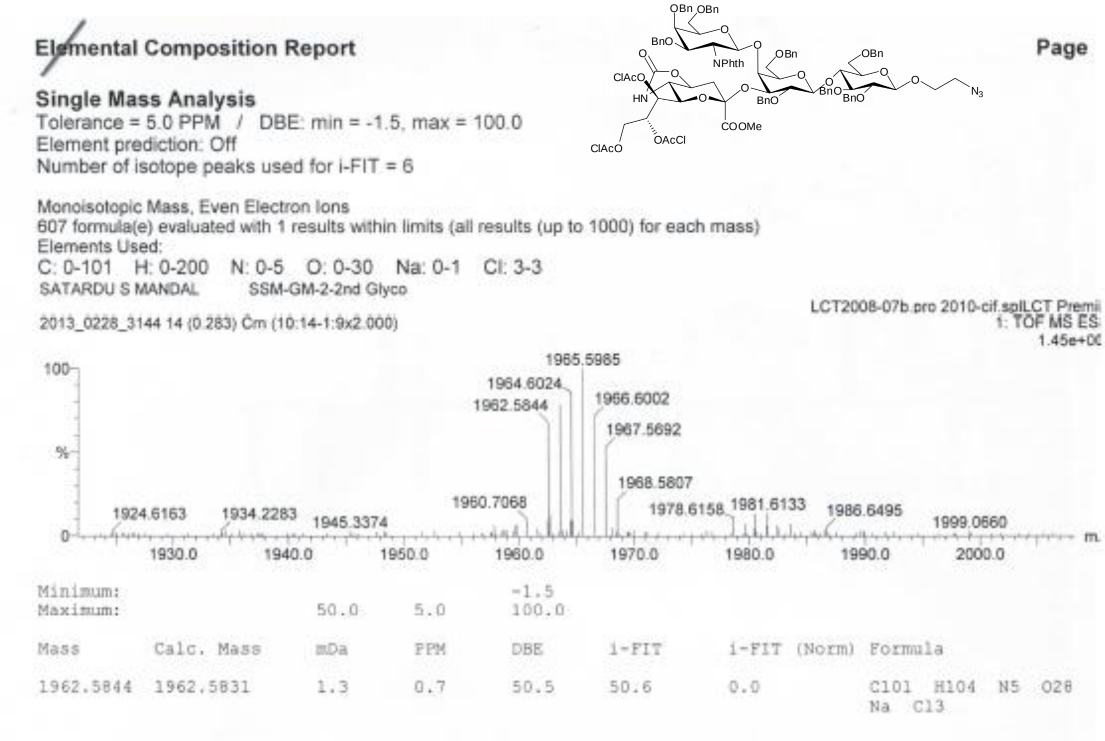

Figure S44. HRMS ESI-TOF MS spectrum of compound **15**

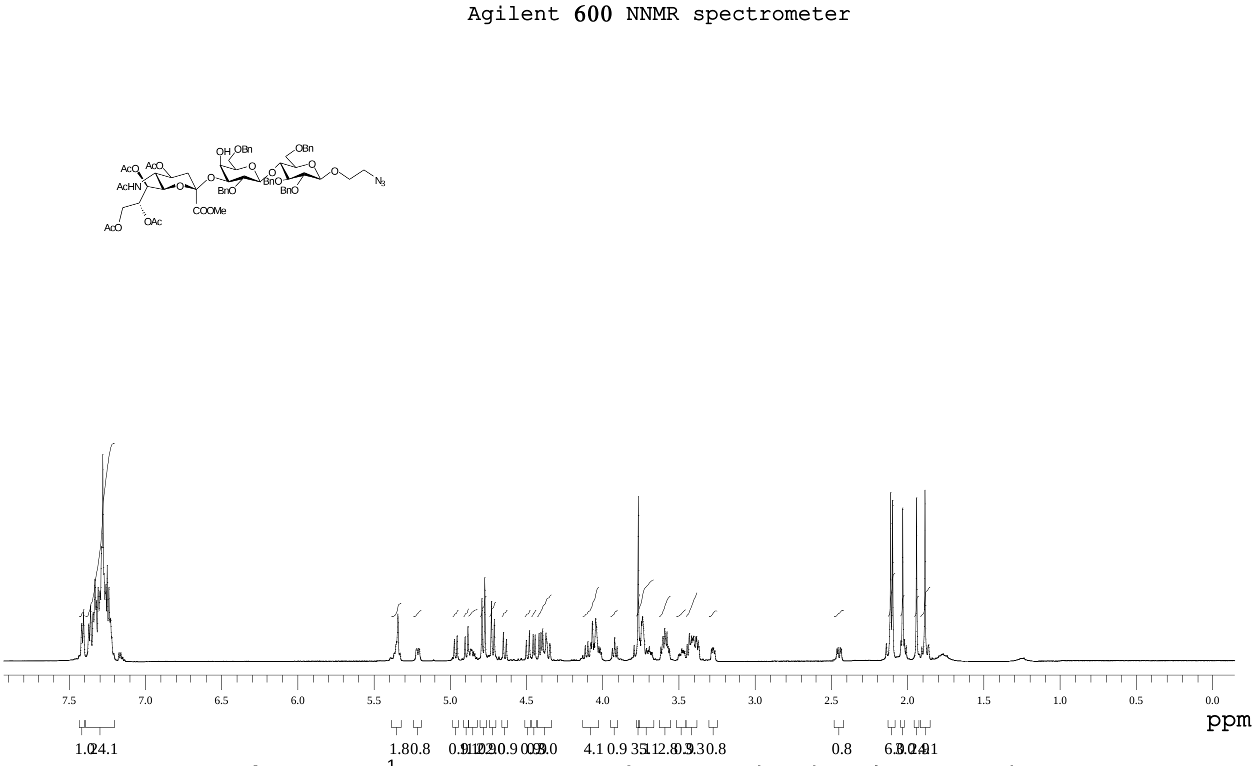

Figure S45. ^{1}H NMR spectrum of compound **16** ($CDCl_3$, 600 MHz)

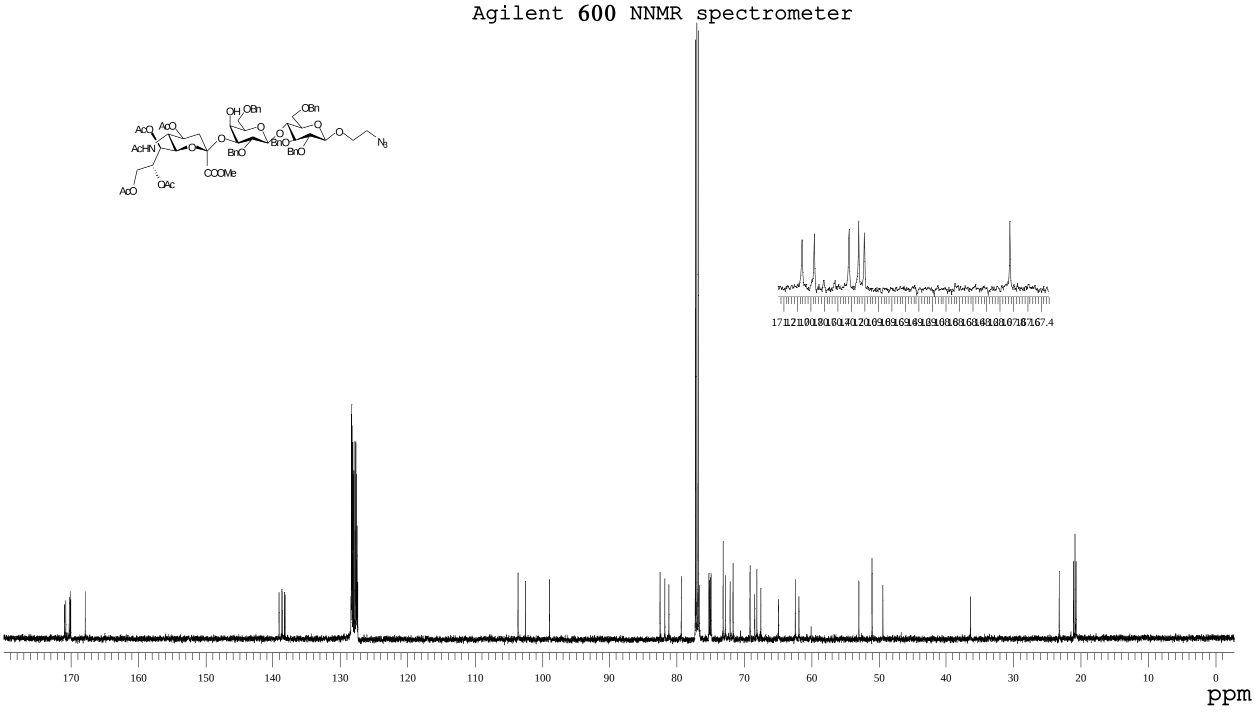

Figure S46. ^{13}C NMR spectrum of compound **16** ($CDCl_3$, 150 MHz)

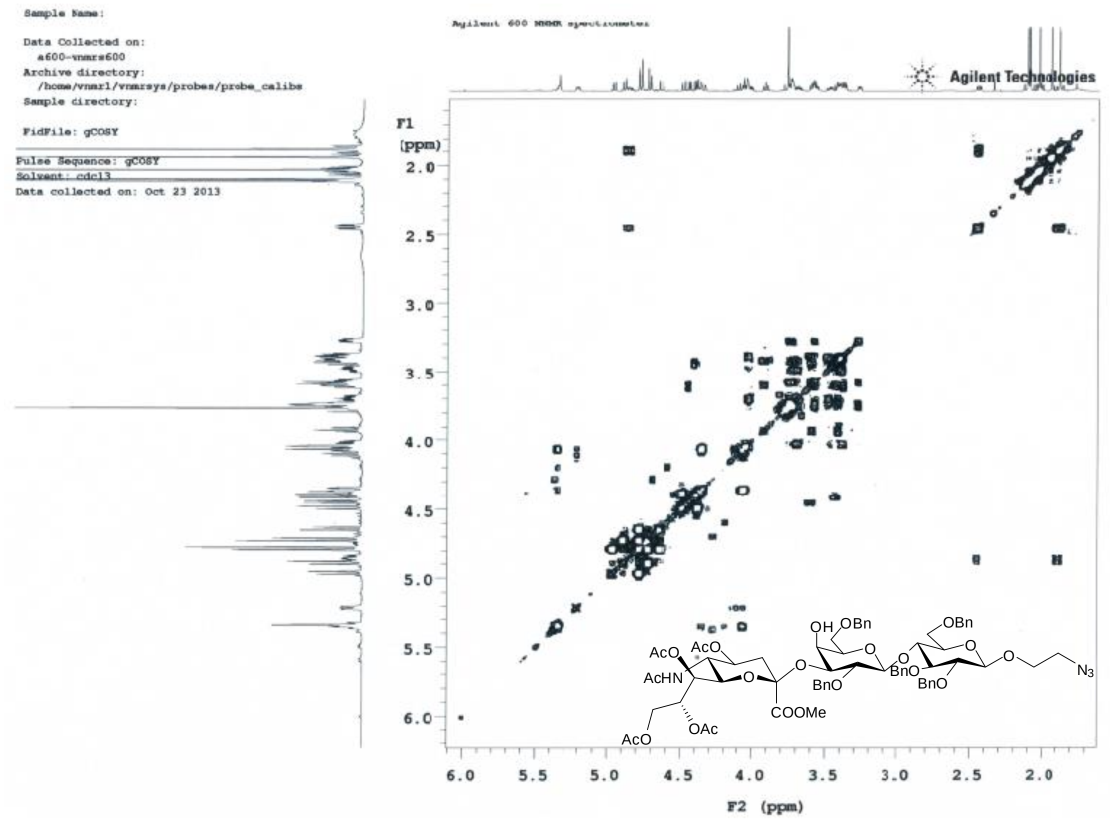

Figure S47. ^{1}H-^{1}H COSY spectrum of compound **16** ($CDCl_3$, 600 MHz)

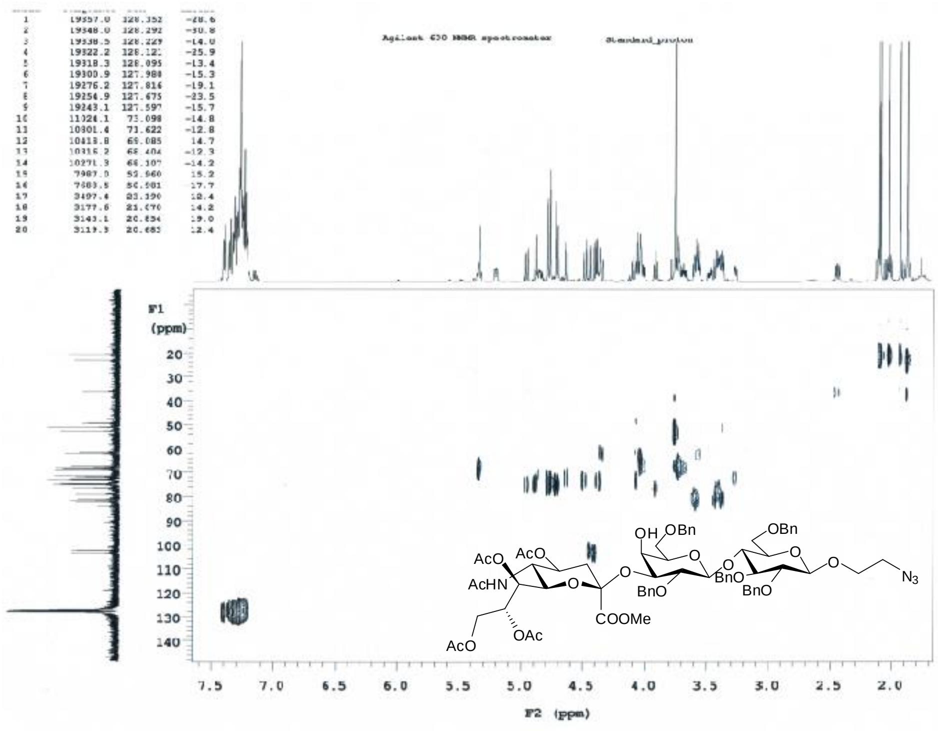

Figure S48. ^{1}H-^{13}C HMQC NMR spectrum of compound **16** ($CDCl_3$, 600/150 MHz)

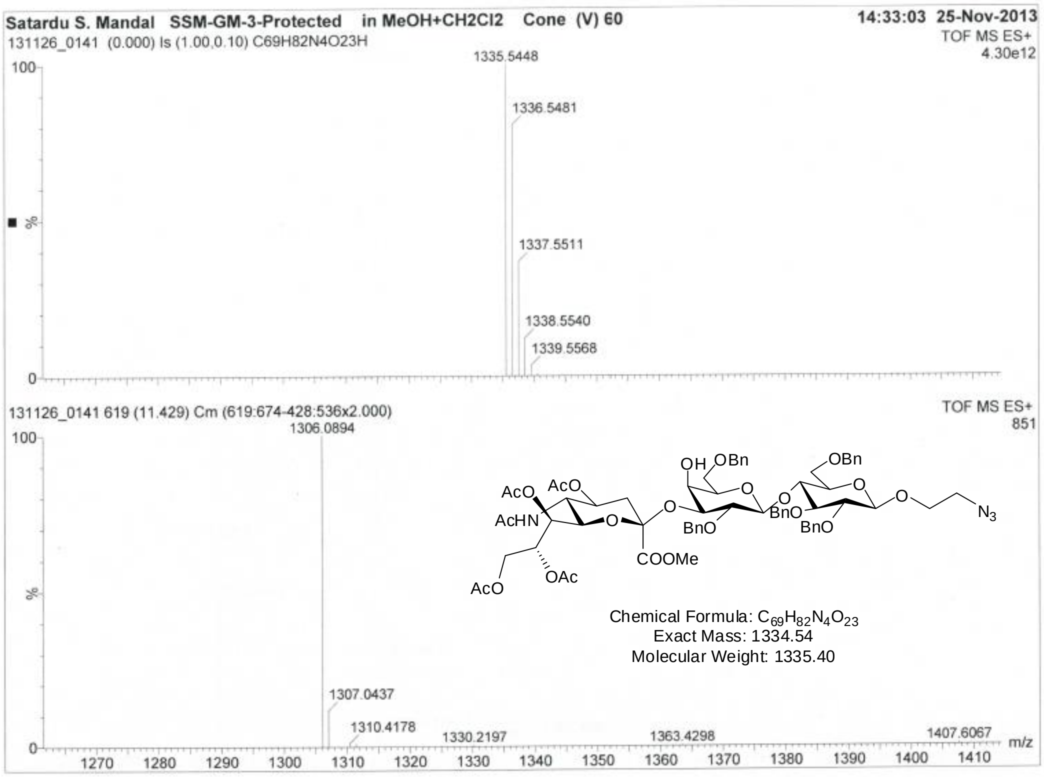

Figure S49. HRMS ESI-TOF MS spectrum of compound **16**

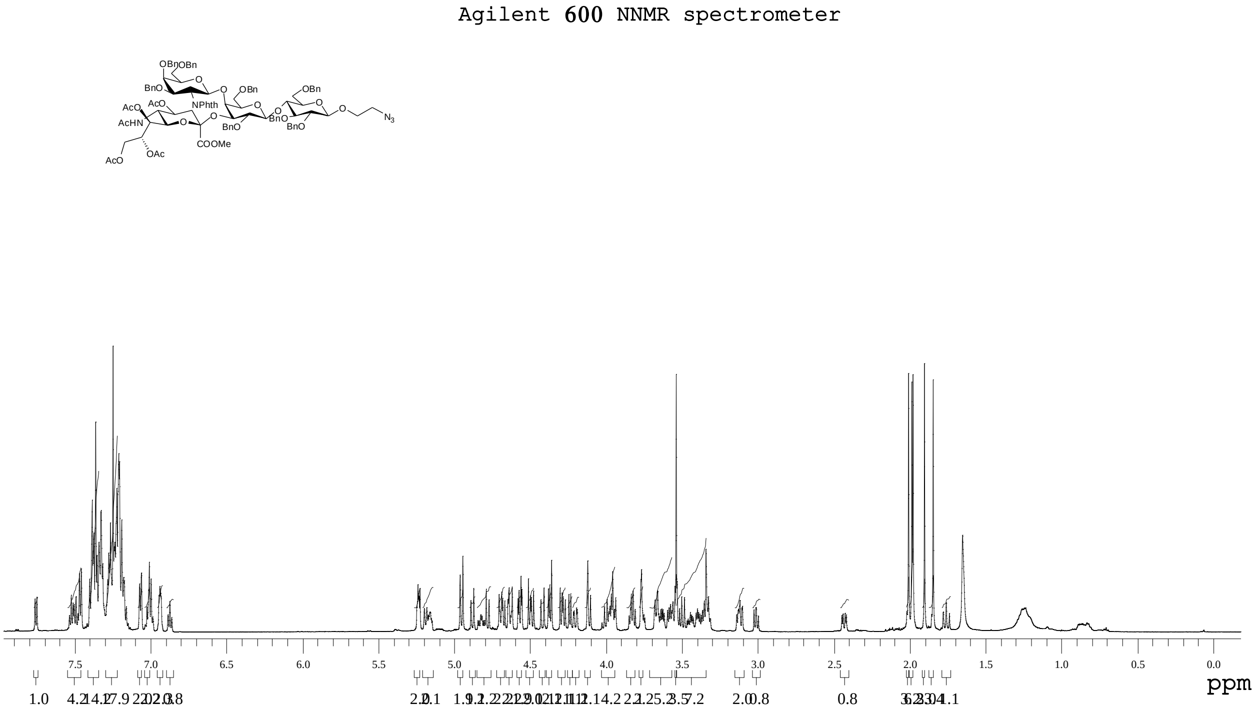

Figure S50. ^{1}H NMR spectrum of compound **17** ($CDCl_3$, 600 MHz)

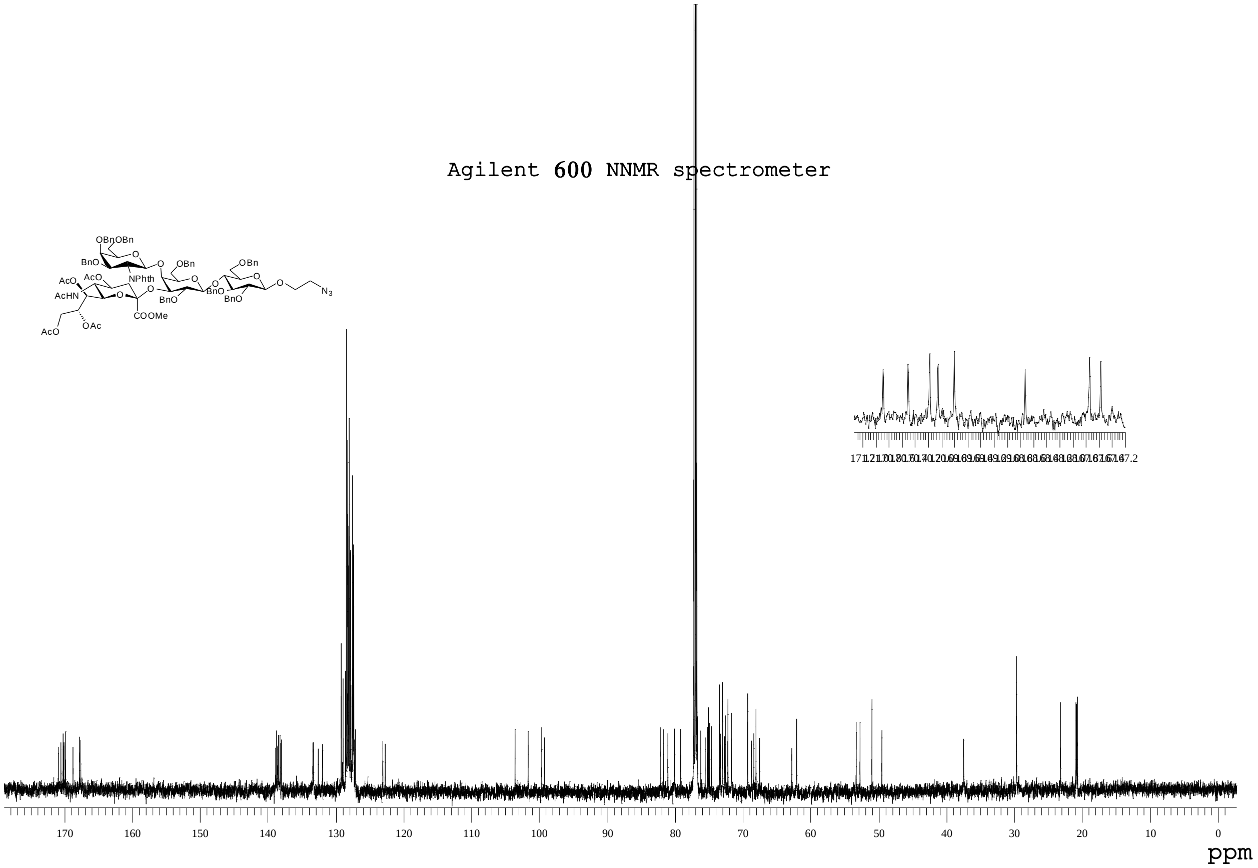

Figure S51. ^{13}C NMR spectrum of compound **17** ($CDCl_3$, 150 MHz)

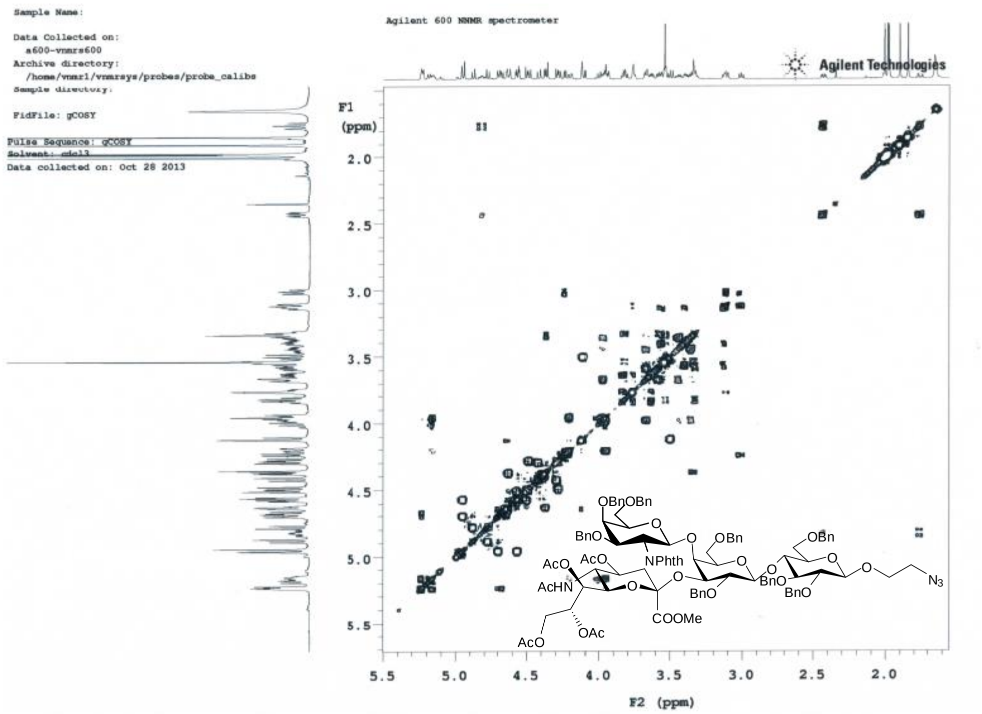

Figure S52. ^{1}H-^{1}H COSY spectrum of compound **17** ($CDCl_3$, 600 MHz)

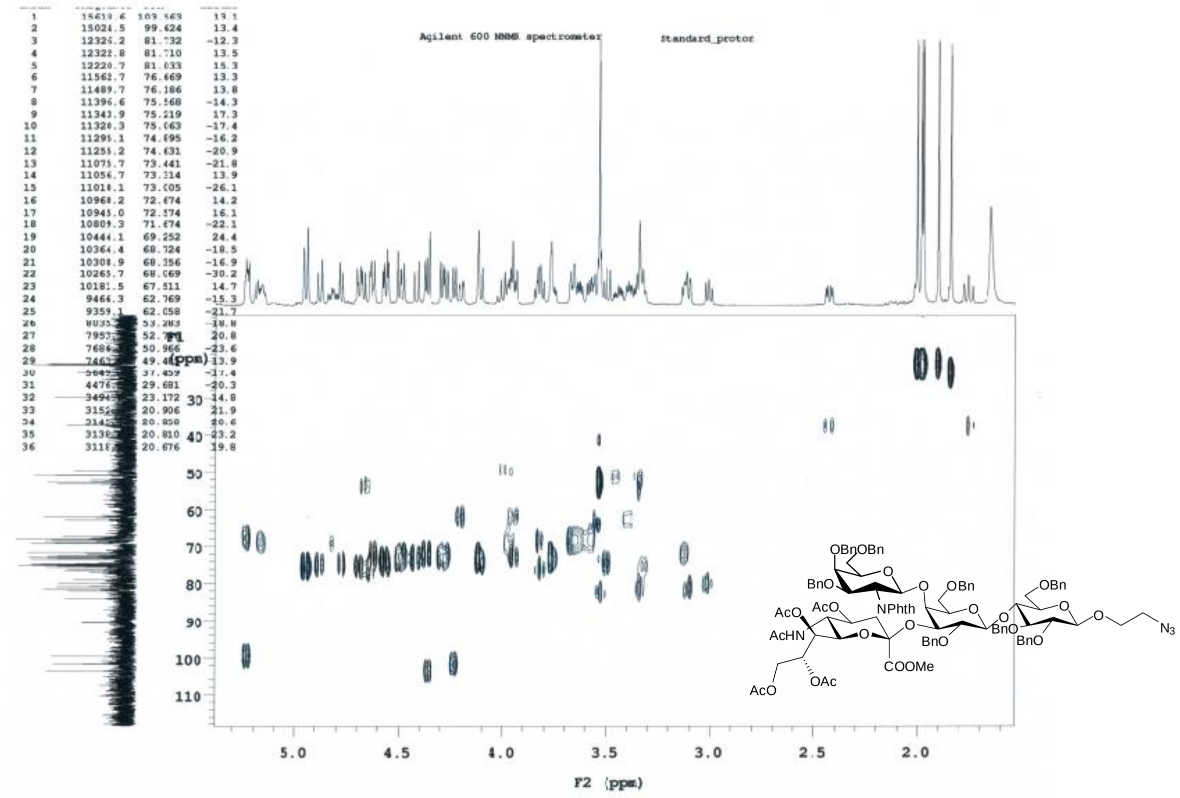

Figure S53. ^{1}H-^{13}C HMQC NMR spectrum of compound **17** ($CDCl_3$, 600/150 MHz)

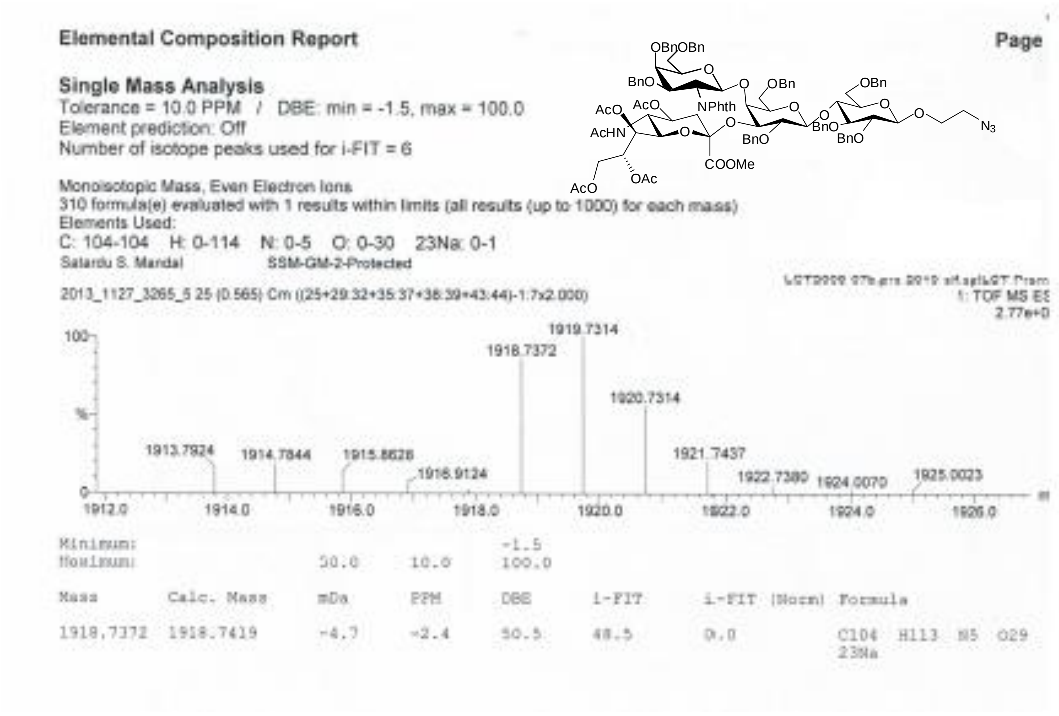

Figure S54. HRMS ESI-TOF MS spectrum of compound **17**.

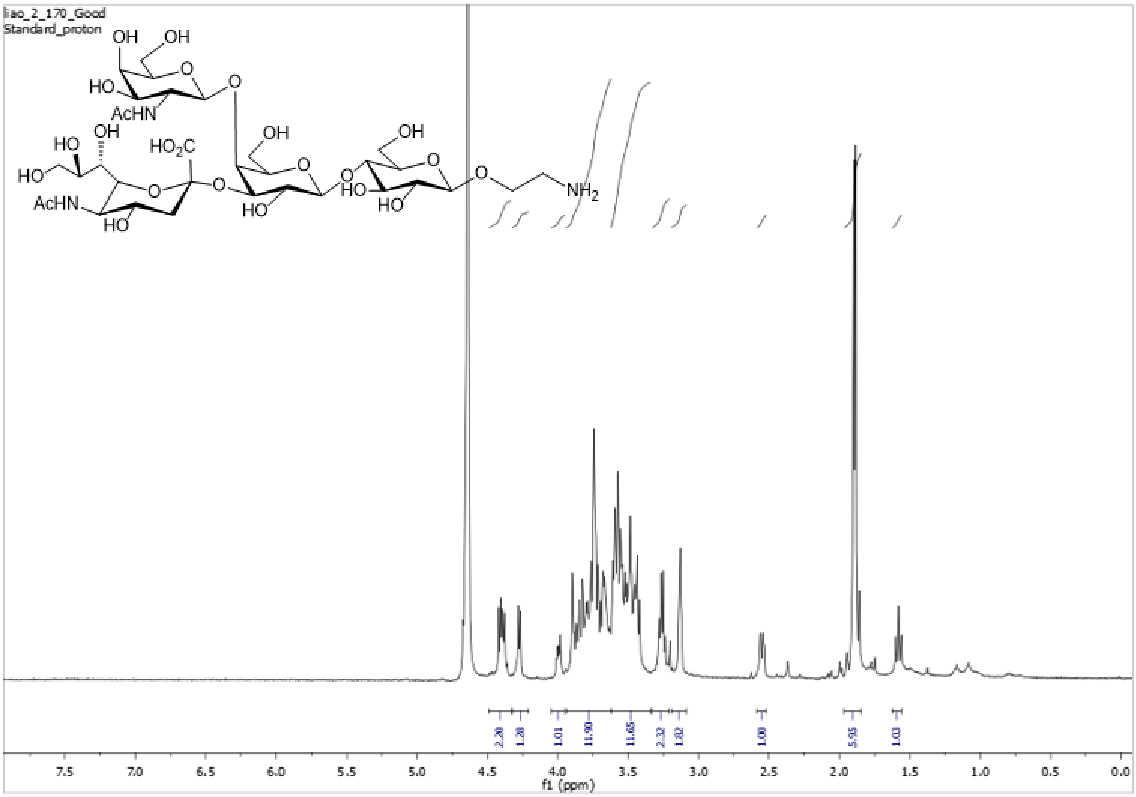

Figure S55. ^{1}H NMR spectrum of compound **1** ($CDCl_3$, 600 MHz)

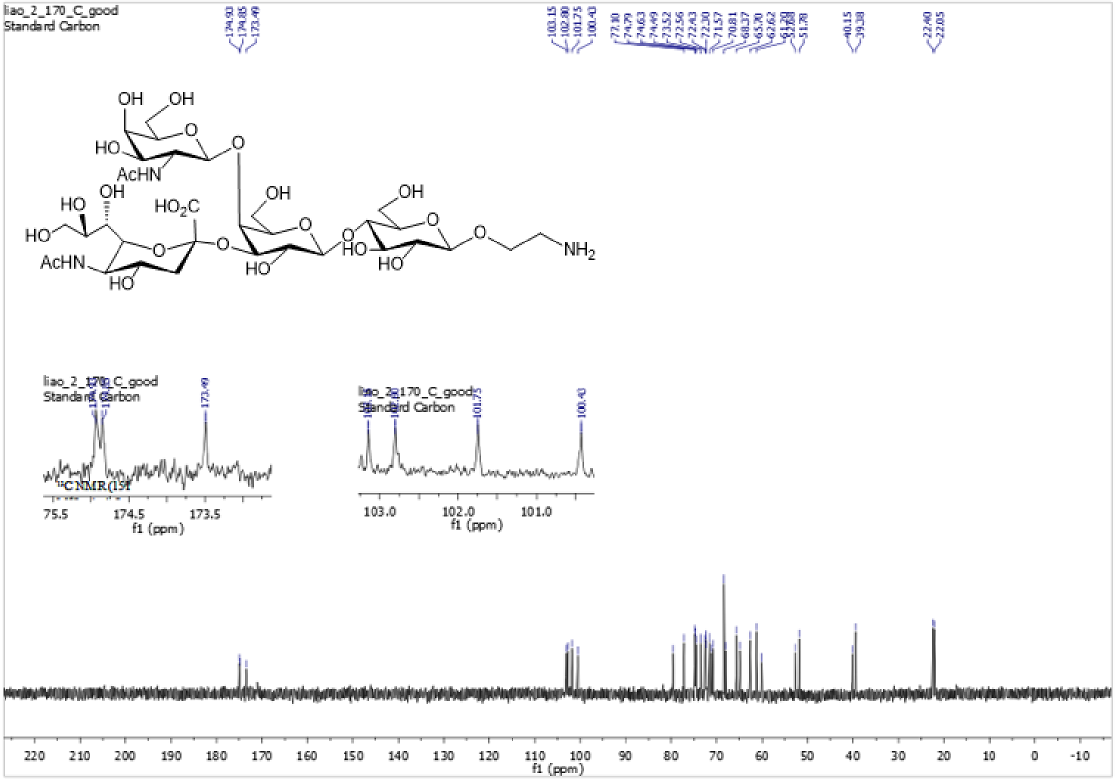

Figure S56. ^{13}C NMR spectrum of compound **1** ($CDCl_3$, 150 MHz)

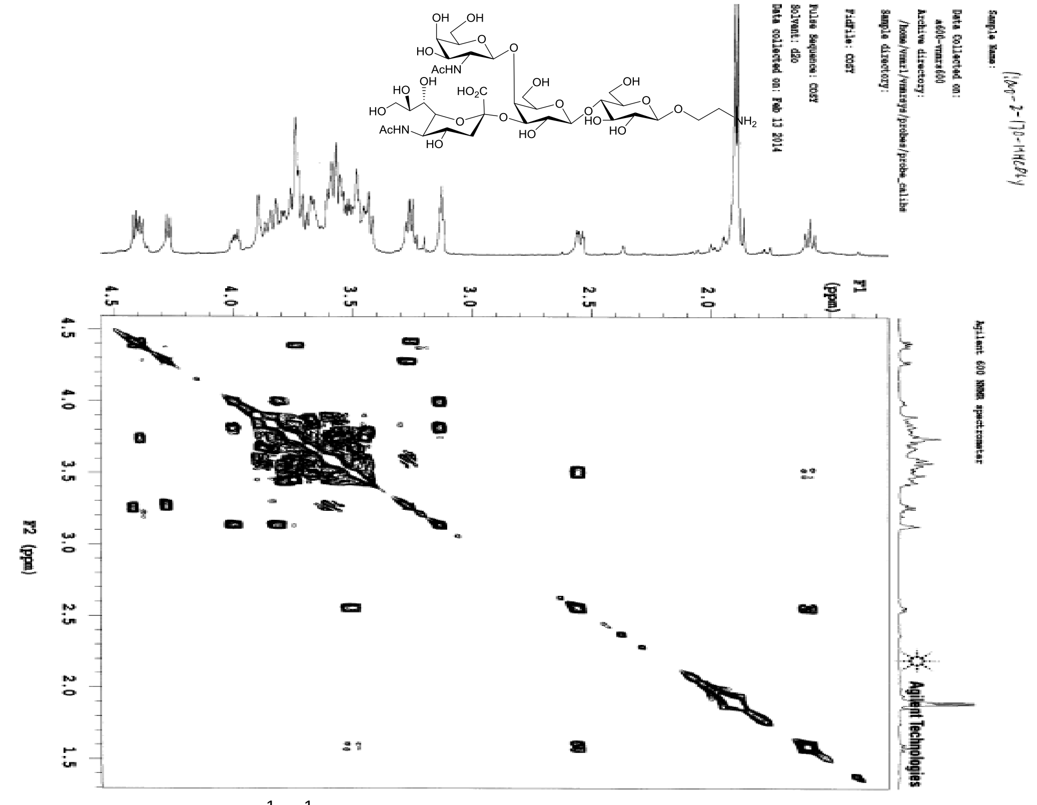

Figure S57. ^{1}H-^{1}H COSY spectrum of compound **1** ($CDCl_3$, 600 MHz)

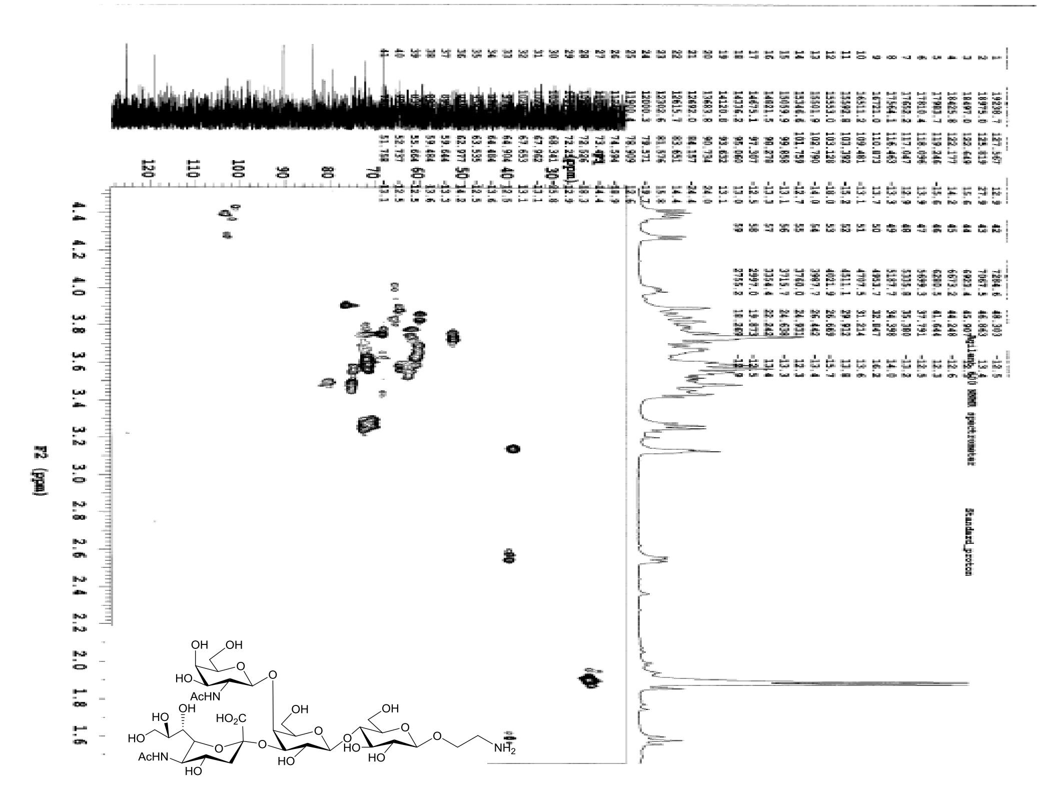

Figure S58. ^{1}H-^{13}C HMQC NMR spectrum of compound **1** ($CDCl_3$, 600/150 MHz)

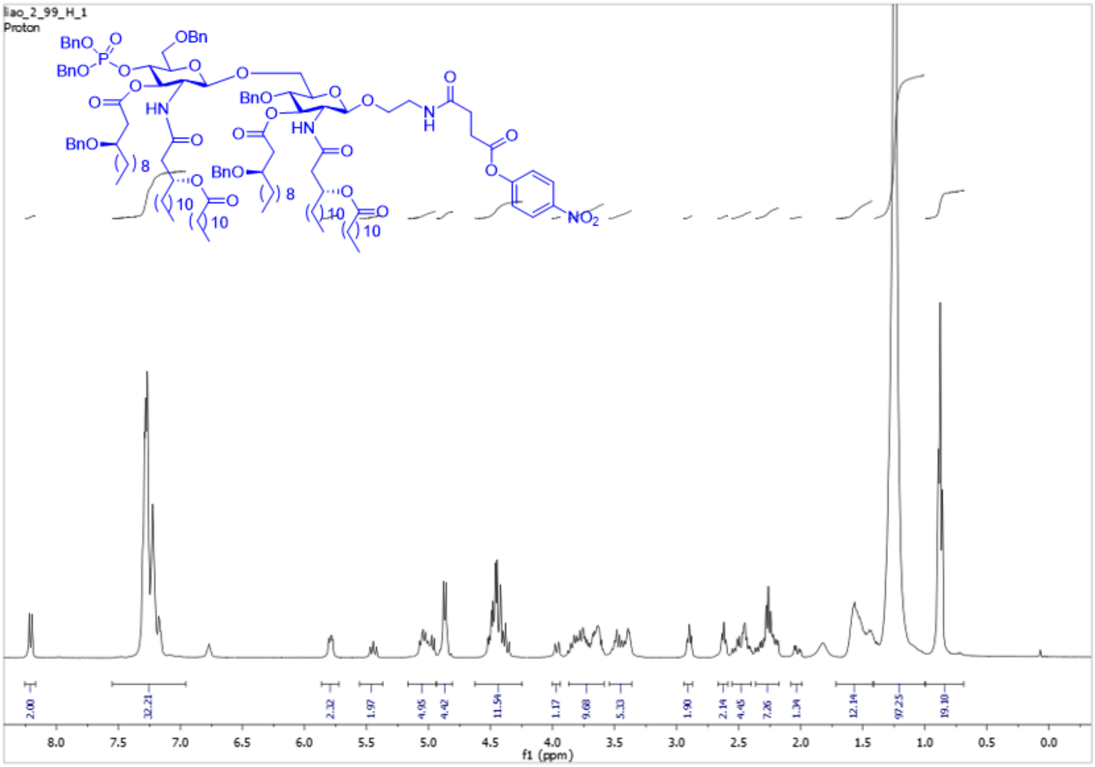

Figure S59. ^{1}H NMR spectrum of compound **19** ($CDCl_3$, 600 MHz)

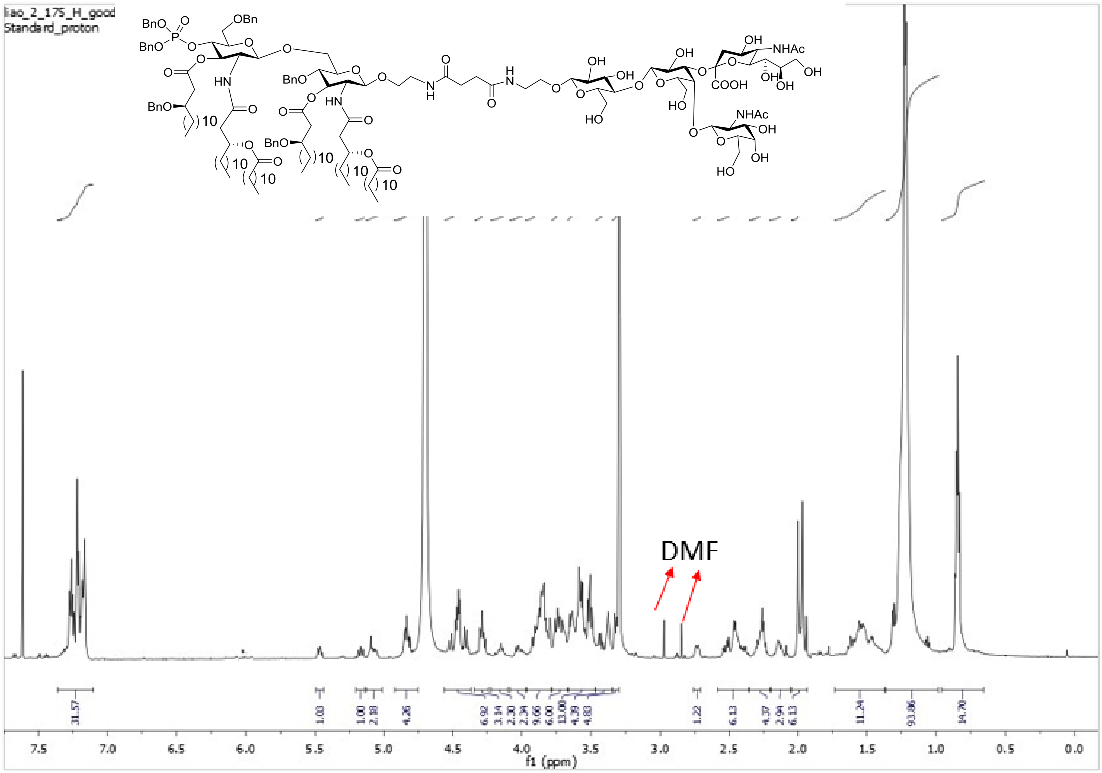

Figure S60. ^{1}H NMR spectrum of compound **20** ($CDCl_3$, 600 MHz)

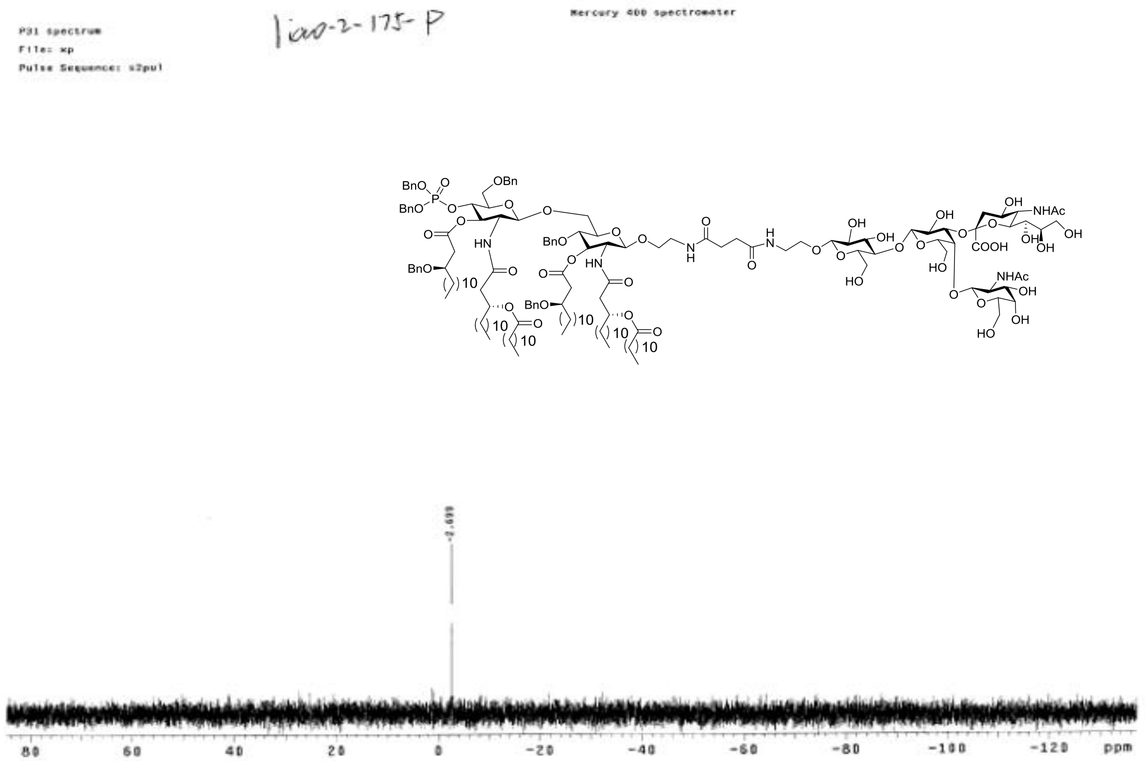

Figure S61. ^{31}P NMR spectrum of compound **20** ($CDCl_3$, 242 MHz)

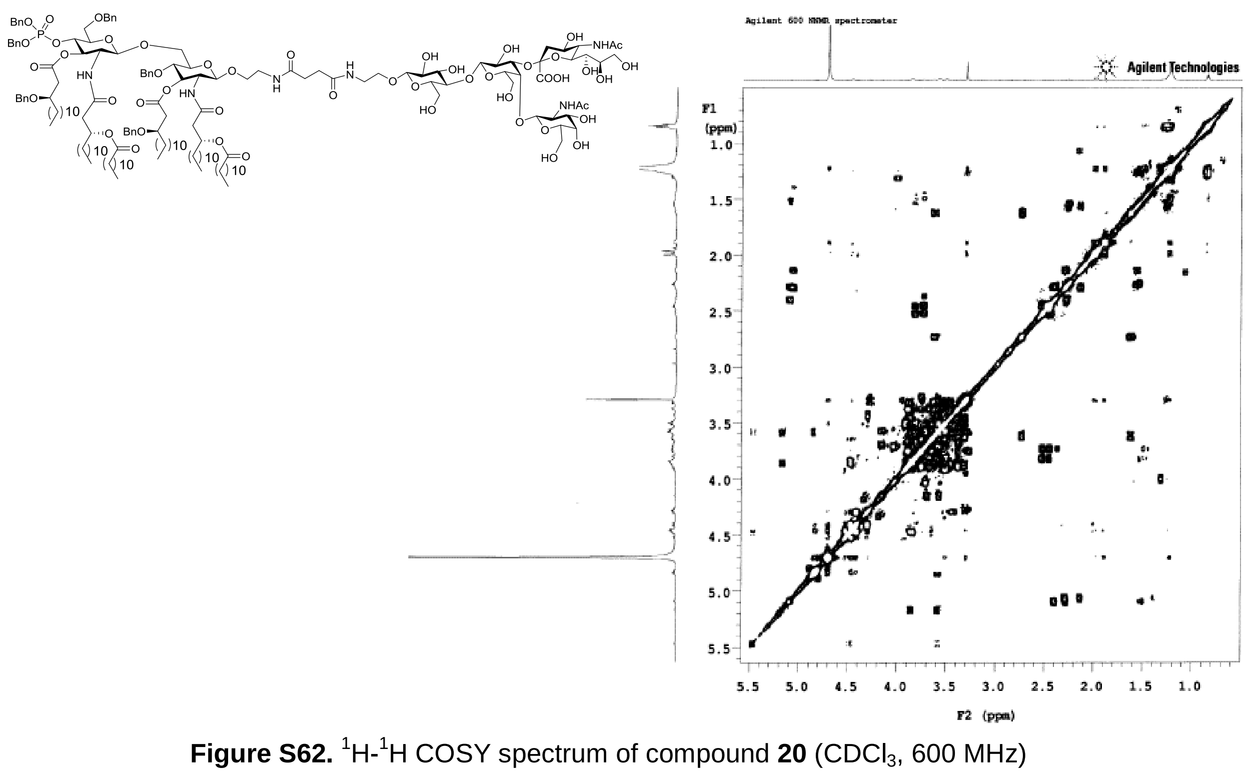

Figure S62. ^{1}H-^{1}H COSY spectrum of compound **20** ($CDCl_3$, 600 MHz)

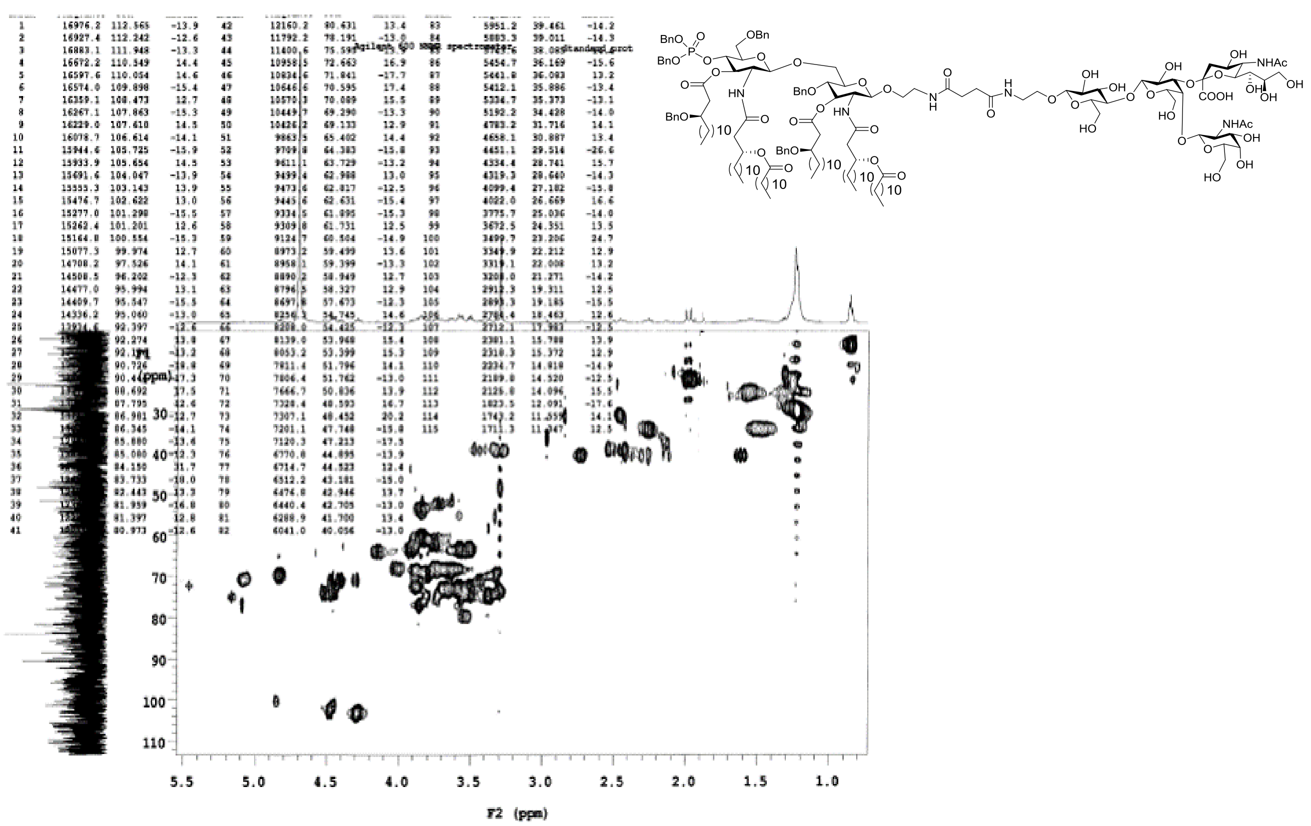

Figure S63. ^{1}H-^{13}C HMQC NMR spectrum of compound **20** ($CDCl_3$, 600/150 MHz)

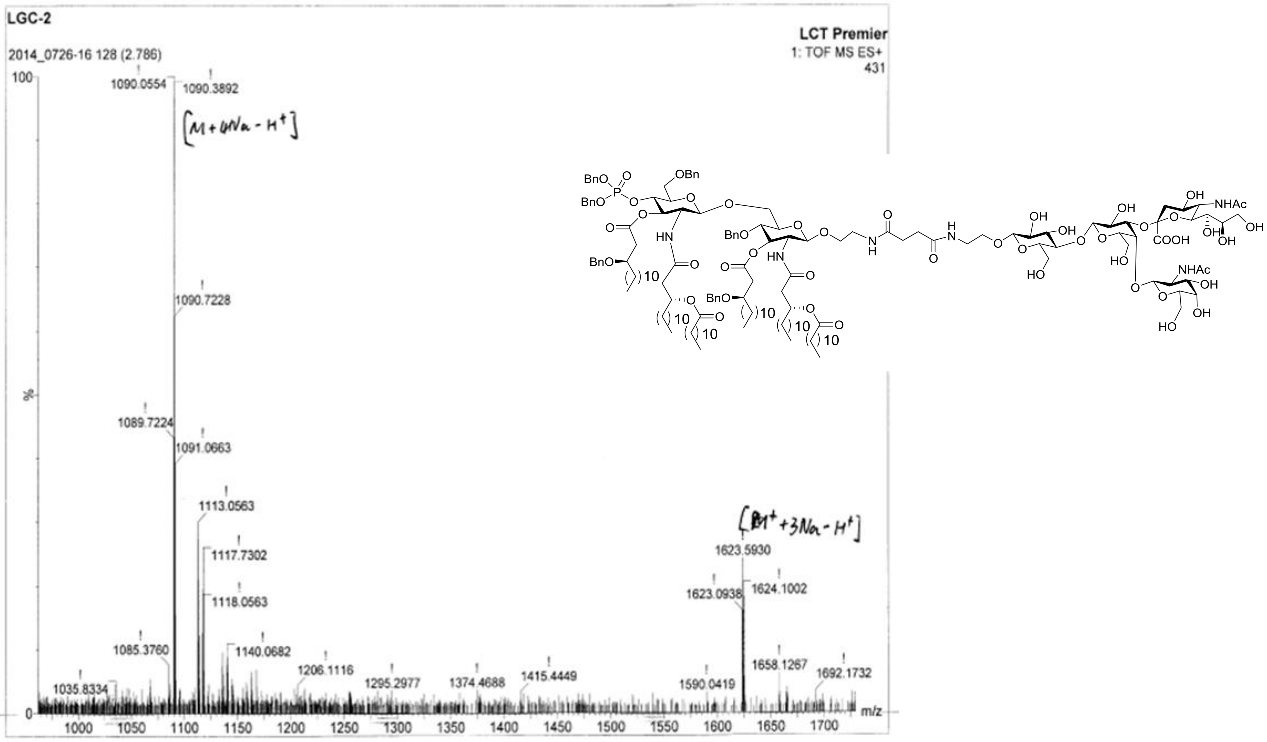

Figure S64. MALDI-TOF MS spectrum of compound **20**

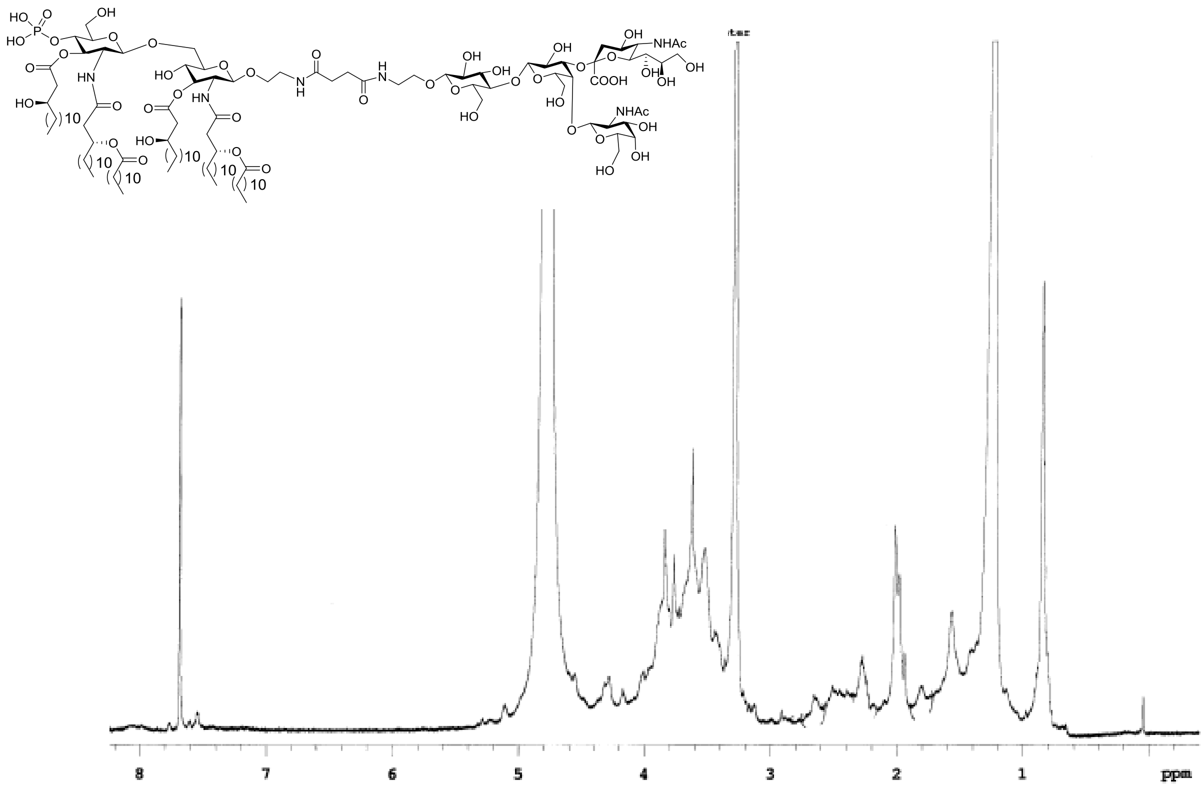

Figure S65. ^{1}H NMR spectrum of compound **2** ($CDCl_3$, 600 MHz)

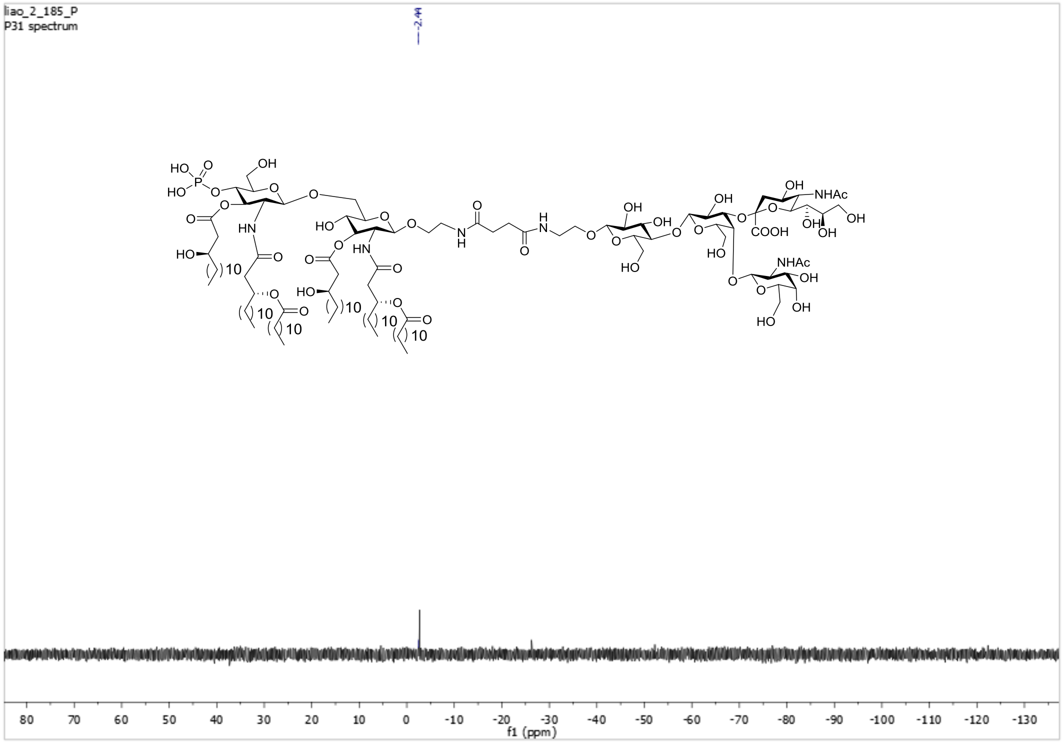

Figure S66. ^{31}P NMR spectrum of compound **2** ($CDCl_3$, 242 MHz)

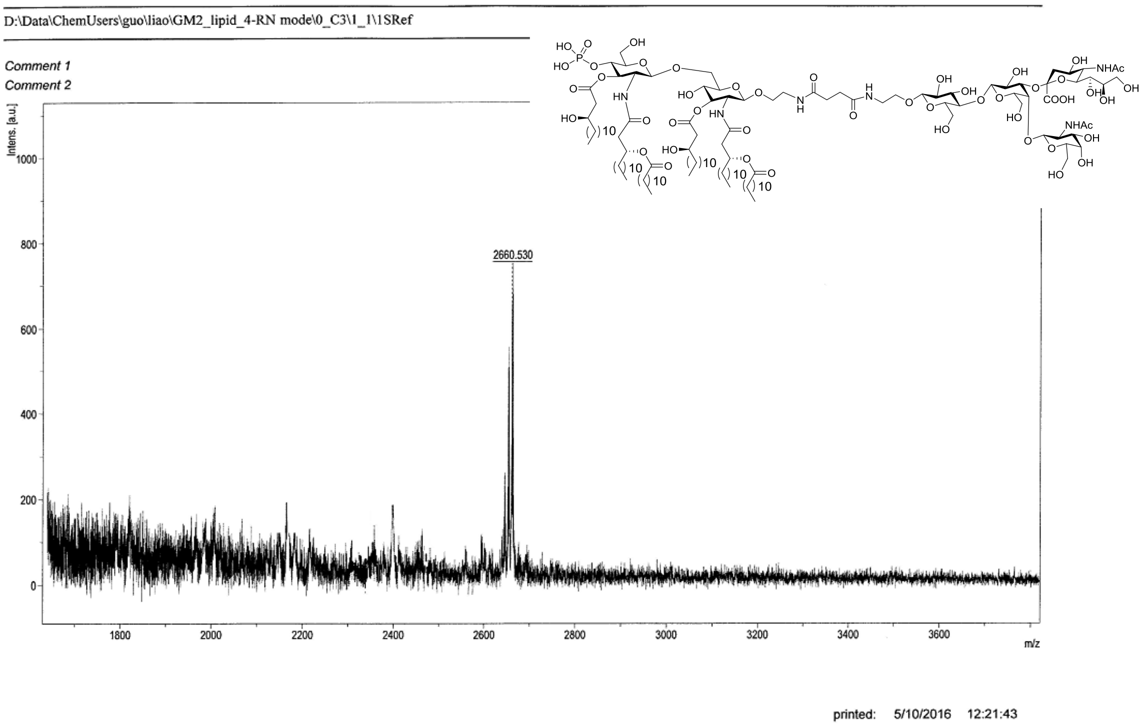

Figure S67. MALDI-TOF MS spectrum of compound **2**

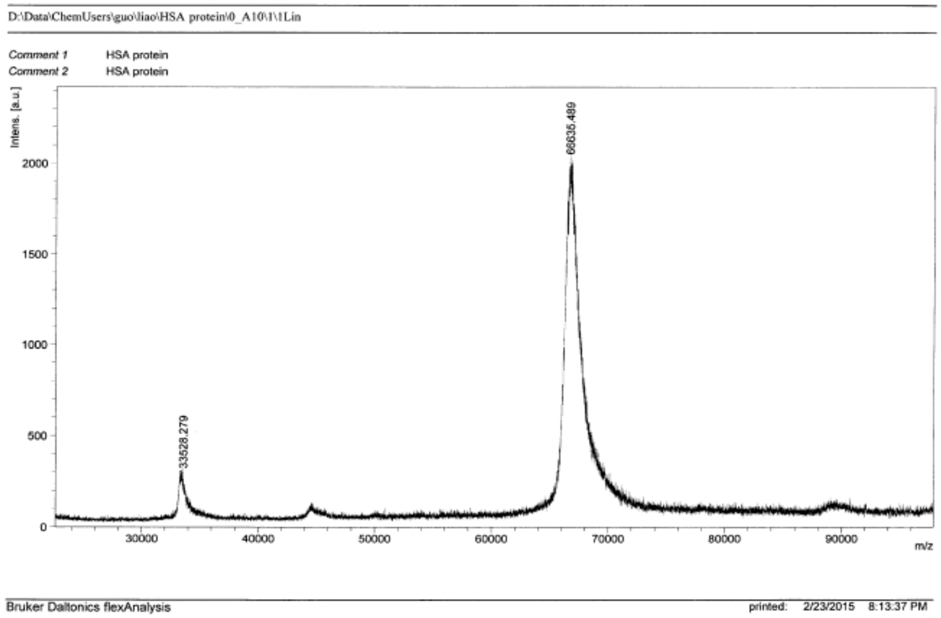

Figure S68. MALDI-TOF MS spectrum of HSA

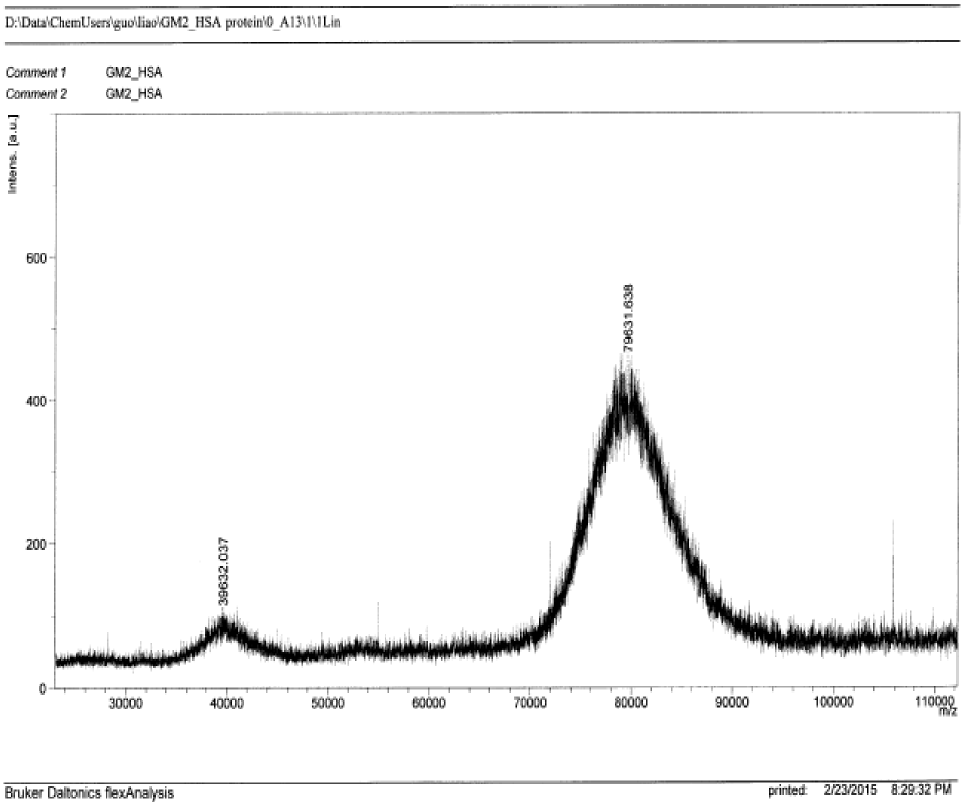

Figure S69. MALDI-TOF MS spectrum of HSA-GM2 conjugate **4**
